# Synthetic Porous Carbons for High-Energy, High-Power Supercapacitors

**DOI:** 10.1021/acs.chemrev.6c00037

**Published:** 2026-07-01

**Authors:** Fan Wang, Nikolaos Samartzis, Tao Wang, Sheng Dai

**Affiliations:** † Department of Chemistry, 4292 The University of Tennessee, Knoxville, Tennessee 37996, United States; ‡ Chemical Sciences Division, 6146 Oak Ridge National Laboratory, Oak Ridge, Tennessee 37830, United States

## Abstract

Porous carbons are premier electrode materials for supercapacitors, yet overcoming the tradeoff between energy and power density remains a fundamental challenge. Guided by the overarching goal of achieving simultaneously high energy and high power, significant progress has been made in the design of advanced carbon materials, accompanied by a deepened mechanistic understanding of electrochemical charge storage at complex interfaces. This Review provides a comprehensive and critical overview of key advances in synthetic porous carbons (SPCs) developed by the broader research community, with an emphasis on materials derived from well-defined molecular and polymeric precursors. Bottom-up synthetic strategies that enable precise control over the pore structure and surface chemistry are highlighted. We summarize fundamental charge storage mechanisms and discuss how hierarchical porosity and heteroatom doping can be rationally engineered to balance energy and power performance. Emerging synthetic methodologies, machine-learning-guided materials design, and advanced in situ and operando characterization techniques are identified as key enablers of predictive and scalable carbon design. These insights outline pathways toward next-generation supercapacitors capable of simultaneous fast charging and high-energy storage.

## Graphical abstract

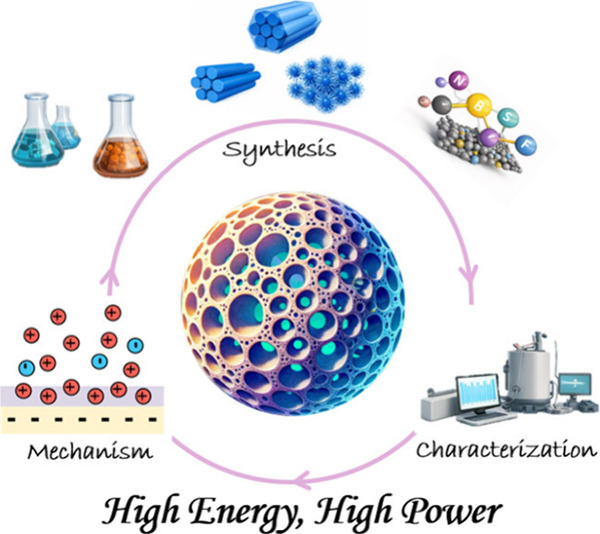

## 1 Introduction

The escalating demand for high-performance energy storage systems, driven by applications from portable electronics to grid-scale stabilization, has intensified research into electrochemical supercapacitors.^1–3^ Recent perspectives have further identified porous materials as a central frontier in next-generation energy technologies, highlighting their critical role in bridging fundamental materials design and practical device performance.^4,5^ These devices offer exceptional power density, rapid charge/discharge capabilities, and long-term cycling stability.^6^ However, their widespread adoption is often constrained by a lower energy density compared to batteries.^7,8^ This limitation is fundamentally rooted in how the electrode structure regulates ion transport and charge storage processes. The electrochemical performance of a carbon-based supercapacitor is governed by a complex interplay between pore architecture, surface chemistry, and ion transport dynamics. In particular, pore size, connectivity, and hierarchical organization determine both the accessibility of electrolyte ions and the kinetics of charge storage processes. Micropores typically serve as the primary sites for charge accumulation through electric double-layer formation, whereas mesopores and larger transport channels facilitate ion diffusion, especially in thick electrodes. Establishing clear structure–property–performance relationships between pore architecture and electrochemical behavior is therefore essential for the rational design of high-energy and high-power carbon electrodes. Within this context, the choice and design of electrode materials become central to addressing this challenge. Porous carbons remain the benchmark electrode material for commercial and next-generation electrochemical double-layer capacitors (EDLCs).^8,9^ Their enduring prominence arises from a compelling combination of properties, including low cost, environmental benignity, excellent electrical conductivity, high specific surface area (SSA), robust chemical and electrochemical stability, and lightweight. While this extensive research laid a critical foundation, these traditional “top-down” methods often yield materials with broad pore size distributions and heterogeneous surface chemistry.^10–12^ This lack of precise control hinders the systematic optimization of materials and obscures the quantitative correlation between pore structure, ion dynamics, and electrochemical response, a prerequisite for simultaneously achieving high energy and high power.^12–14^

To overcome these limitations, the field has rapidly evolved toward SPCs derived from well-defined molecular or polymeric precursors.^15,16^ Recent advances in materials synthesis, mechanistic understanding, and characterization have enabled unprecedented control over pore structure and chemistry.^17–19^ This “bottom-up” paradigm provides an unprecedented level of precision and tunability over the final carbon architecture.^20,21^ More importantly, such precise control enables systematic investigation of how pore size, connectivity, and surface chemistry influence ion transport kinetics and charge storage behavior. Templating methods that utilize either soft scaffolds such as block copolymers or hard templates such as silica nanoparticles allow the fabrication of ordered mesoporous carbons with uniform pore architectures.^22,23^ Similarly, ionothermal synthesis has become a versatile route to generate highly porous carbons by employing eutectic molten salts as both templates and activating agents.^24,25^ The pursuit of ultrahigh surface areas has pushed design limits, with hyperporous carbons exceeding 2630 m^2^ g^–1^ achieved through zeolite templating^26^ or carbonization of hypercross-linked polymers.^27^ These synthetic innovations are complemented by advanced characterization and modeling tools that provide molecular-level insights into ion dynamics within complex pore networks. At the same time, machine-learning-guided discovery^20,28–31^ is accelerating materials design, as exemplified by the prediction and synthesis of oxygen-rich hyperporous carbons with exceptional capacitance identified through artificial neural networks.^32^ Together, these developments highlight the field’s transition toward precision-controlled and knowledge-driven synthesis.^33–37^ In addition, the structures of their carbon products have been further optimized through diverse approaches ranging from conventional pyrolysis and chemical activation to more advanced techniques such as laser irradiation^38^ and flash Joule heating.^39^ These methods allow for deliberate control over pore size and distribution, network topology, surface chemistry (e.g., heteroatom doping),^40,41^ and the degree of graphitization.^17–19,25,40–42^ Such multidimensional tunability provides a platform to correlate structural parameters with ion transport processes and ultimately electrochemical performance. This ability to precisely engineer the carbon framework is the central theme of this Review. It allows for the creation of ideal model systems to probe fundamental ion transport and charge storage mechanisms at the nanoscale. Futhermore, it provides a powerful platform for systematically correlating material structure with electrochemical performance, guiding the “materials-by-design” approach needed to develop next-generation supercapacitors that reconcile high power and high energy.

To maintain a coherent and focused scope, two major classes of carbon-based materials are excluded from this review. First, biomass-derived carbons, while industrially significant, are omitted due to their compositional complexity and the vast, distinct body of literature they encompass.^43–46^ Second, hybrid materials such as carbon/metal oxide and carbon/metal are not discussed, as their charge-storage mechanisms are dominated by Faradaic redox processes rather than the predominantly capacitive behavior characteristic of porous carbons.^47–51^ These boundaries allow for a more rigorous and mechanistic exploration of SPCs optimized for high-energy, high-power supercapacitors. Accordingly, this Review is organized around the structure–transport–performance relationship, with emphasis on how pore architecture governs ion dynamics and electrochemical behavior. In the sections that follow, we first provide a historical perspective on charge-storage mechanisms in carbon-based supercapacitors, followed by an analysis of the fundamental principles and challenges governing fast ion transport and high-rate performance. We then present a detailed discussion of advanced synthetic strategies for tuning pore architecture, surface functionality, and heteroatom incorporation. Finally, we highlight state-of-the-art characterization techniques and emerging data-driven approaches that are transforming the discovery and understanding of high-energy, high-power porous carbons, and conclude with a forward-looking outlook on future opportunities in this rapidly evolving field.

## 2 Energy Storage Mechanism/Theory of Carbon-Based Supercapacitors

The ability of porous carbons to store charge rapidly is governed by the interplay of two fundamental kinetic processes: surface-controlled and diffusion-controlled kinetics. While this classification provides a convenient and widely adopted framework for interpreting electrochemical behavior, it should be understood as an operational description of apparent kinetics rather than a strict separation of the underlying mechanisms. Surface-controlled processes relate to the adsorption and desorption of ions at the electrode–electrolyte interface, forming the electrochemical double layer, and are inherently fast. In contrast, diffusion-controlled processes involve the transport of ions from the bulk electrolyte through the porous network of carbon to access the active surfaces. In practical porous carbon systems, however, these processes are intrinsically coupled and often coexist with additional physicochemical phenomena such as ion desolvation, ion exchange, confinement effects, and pseudocapacitive reactions. The overall rate capability of an electrode is thus determined by how efficiently these two processes are managed, which is intrinsically linked to the material’s pore architecture, surface chemistry, and conductivity.

The understanding of capacitive charge storage began with the establishment of classical electric double-layer (EDL) theories by Helmholtz,^52^ Gouy,^53^ Chapman,^54^ and Stern[Bibr ref55] (1853–1924), which described interfacial charge accumulation either as a compact layer or as a diffuse cloud of counterions (Figure 1). These idealized models were later refined by nonclassical EDL frameworks proposed by Frumkin,^56^ Grahame,^57^ and Bockris^58^ between 1928 and 1963, introducing specific ion adsorption, inner/outer Helmholtz planes, and solvent dipole orientation effects.

**Figure 1:**
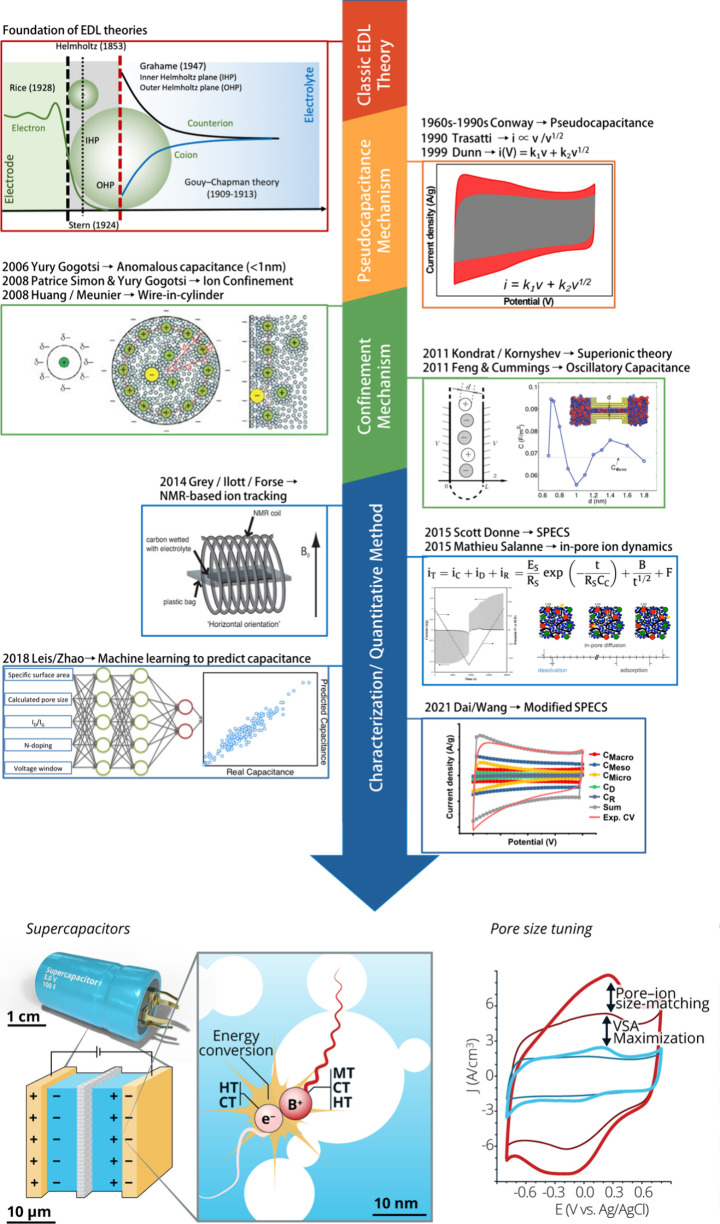
Development timeline of carbon-based supercapacitors mechanisms.^5,67,71,73–80^ Reproduced with permission from refs 5, 67, 71, 73, 74–80 . Copyright 2025 American Association for the Advancement of Science, Copyright 2008 Springer Nature, Copyright 2011 American Chemical Society, Copyright 2022 American Chemical Society, Copyright 2011 IOP Publishing, Copyright 2013 Royal Society of Chemistry, Copyright 2015 American Chemical Society, Copyright 2015 Elsevier, Copyright 2018 Elsevier, Copyright 2021 The Electrochemical Society.

In the 1960s–1990s, Conway revolutionized the field by defining pseudocapacitance as fast, surface-confined Faradaic charge storage, bridging the gap between double-layer and battery-type mechanisms.^59–61^ This was followed by quantitative kinetic models developed by Trasatti (1990),^62,63^ who introduced the i ∝ v /v^1/2^ method to deconvolute surface-controlled and diffusion-controlled processes, and later by Dunn (1999) who formulated the widely adopted i­(V) = k_1_v + k_2_v^1/2^ fitting approach for cyclic voltammetry analyses.^64,65^ Although these models are powerful tools for quantifying apparent kinetic contributions, they inherently project complex, multistep processes onto simplified scaling relationships and do not explicitly resolve nanoscale phenomena such as confinement, solvation structure, or ion–ion correlations within pores.

A paradigm shift occurred in 2006, when Yury Gogotsi reported anomalous capacitance enhancement in subnanometer pores,^66^ challenging the validity of the Gouy–Chapman–Stern picture under extreme confinement. Subsequent collaborative work by Simon and Gogotsi in 2008 established the ion confinement model, attributing capacitance increase to partial desolvation and dense ion packing within carbon nanopores.^67,68^ Parallel theoretical efforts by Huang and Meunier proposed the wire-in-cylinder pore model,^69,70^ while Kondrat and Kornyshev (2011) introduced the superionic state theory,^71,72^ describing confined ions as quasi-metallic charge carriers. Feng and Cummings further predicted oscillatory capacitance–pore size relationships, arising from ion layering transitions.^73^

To experimentally validate these confined charging mechanisms, Scott Donne developed the SPECS method (2015) for separating geometric surface and pore contributions,^78^ whereas Gray, Ilott, and Forse employed operando NMR spectroscopy to directly track ion adsorption and ion-exchange dynamics within pores.^76,81–83^ Complementary to this, Mathieu Salanne (2015) established a hierarchical time scale model for in-pore ion transport through constant-potential molecular dynamics.^77^ In the past decade, emerging data-driven approaches such as those by Leis,^84^ Zhao[Bibr ref79] (2018) have applied machine learning to predict capacitance from pore structures, while Dai and Wang (2021) further refined the quantification of capacitance contributions from distinct pore regimes via modified SPECS protocols.^80^

These advances collectively indicate that charge storage in porous carbons arises from multiple interdependent processes spanning different length and time scales. Within this context, the “surface-controlled vs diffusion-controlled” framework should be interpreted as a macroscopic descriptor of dominant kinetic behavior, while the underlying mechanisms such as ion desolvation, ion exchange, confinement-induced restructuring, and pseudocapacitive reactions, govern how this behavior emerges.

Despite their diverse origins ranging from classical double-layer formation to pseudocapacitive redox and confinement-induced ion packing, all charge storage mechanisms in porous carbons can still be usefully analyzed in terms of surface-dominated and transport-limited responses, particularly in electrochemical measurements. Fast charging is achieved when the dominant contribution arises from surface-confined interactions that require minimal ionic transport, whereas rate limitations emerge when ion migration through the pore network becomes the bottleneck.

Beyond these historical developments, the fundamental electrostatic principles of double-layer formation further emphasize why pore architecture sits at the core of charge storage in carbon-based supercapacitors. The cell capacitance (C ∝ εA/d) (where ϵ is the relative dielectric permittivity, A is the area of the interface, and d is the separation between charges) highlights the dual importance of maximizing ion-accessible surface area while minimizing the effective charge-separation distance, making micropores crucial for creating dense interfacial regions and mesopores essential for sustaining rapid ion transport.^5^ Importantly, the accessibility of this surface area is not purely geometric but depends on ion desolvation energetics, pore size matching, and electrolyte-dependent confinement effects, further coupling interfacial and transport processes. Recent advances have shown that subnanometer ultramicropores—previously considered inaccessible—can substantially enhance capacitance through partial ion desolvation and tightly packed ionic layers under extreme confinement, challenging classical EDL descriptions and redefining what constitutes “accessible” surface area in nanoporous carbons. To simultaneously achieve high energy and high power, SPCs must therefore balance confinement-enhanced capacitance with efficient mass transport by precisely tuning pore size distributions, interconnected pathways, and surface chemistry. To avoid ambiguity in the discussion of charge storage mechanisms, it is important to distinguish between three regimes: (i) electric double-layer capacitance (EDLC), arising from non-Faradaic ion adsorption and representing the primary mechanism in porous carbons; (ii) heteroatom-induced pseudocapacitive contributions, involving fast, surface-confined redox reactions; and (iii) hybrid or battery-type systems, which rely on bulk Faradaic processes and are mechanistically distinct. In this review, we focus on EDLC-dominated charge storage in SPCs, while treating pseudocapacitive effects as complementary, and excluding battery-type mechanisms from the main scope unless otherwise specified. Within this framework, the apparent transition between surface-controlled and diffusion-controlled regimes reflects how effectively ion transport, interfacial charging, and confinement effects are coordinated within the porous architecture, as discussed in the following sections.

### 2.1 Surface-Controlled Kinetics

The primary mechanism for energy storage in EDLCs is the physical adsorption of electrolyte ions onto the conductive surface of the electrode, forming an EDL (Figure 2).^74^ This non-Faradaic process is rapid and highly reversible, enabling fast charge–discharge cycles, often within seconds, which is fundamental to achieving the high-power density required for applications like electric vehicles and consumer electronics.^85^ This surface-controlled process is the essence of supercapacitor performance, involving the electrostatic accumulation of cations and anions at negatively and positively polarized electrodes, respectively.

**Figure 2:**
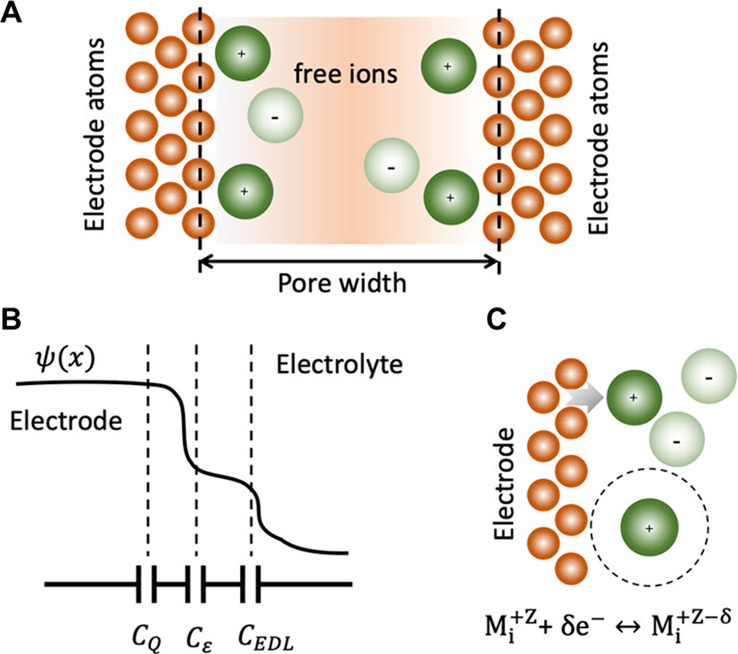
Schematic illustration of an electric double layer. A) Schematic of an electrolyte solution in a porous electrode defined as an open system, i.e., a system with fixed chemical potentials of electrons (μe), solvent molecules (μs), and ionic species (μ ± ). B) Local electrical potential and various contributions to the total capacitance: quantum (CQ), dielectric (Cϵ), and diffuse layer capacitance (CEDL). C) Charge transfer between the electrode atoms (Mi) and adsorbed ions represented by faradaic reactions.^74^ Reproduced with permission from ref 74. Copyright 2022 American Chemical Society.

Classic EDL theories, such as the Gouy–Chapman–Stern model, describe a decay in ionic charge concentration over a characteristic Debye length (typically 1–10 nm) from the electrode surface. However, these traditional models show limitations when applied to the confined environments of nanoporous carbons and high-concentration electrolytes, where factors like ionic correlations and the finite accessible surface area for ions become significant.^86,87^ Advanced mean-field theories and molecular simulations have revealed that the differential capacitance is not constant, but can exhibit “camel” or “bell” shaped curves depending on the electrolyte, highlighting the critical role of the electrolyte’s structure at the electrode interface.

In confined nanopores with dimensions comparable to the ion diameter, charge storage involves more complex mechanisms beyond simple adsorption, including ion swapping, counterion adsorption, or coion desorption.^72,81^ The dominant mechanism is influenced by the applied voltage, electrolyte properties, and kinetic factors, with ion swapping often occurring at low polarization and counterion adsorption dominating at higher voltages. These processes are inherently coupled with local ion desolvation and short-range ion rearrangement and collectively contribute to the observed surface-controlled response when they proceed without long-range transport limitations.

Recent work has further illuminated the crucial role of structural disorder in enhancing capacitance and promoting fast surface-controlled kinetics. *In situ* experimental techniques, including NMR and SAXS, have confirmed that microporous carbons can support extremely fast charging with minimal kinetic lag.^87,89^ More specifically, NMR studies have demonstrated that carbons with smaller graphitic domains (i.e., more structural disorder) exhibit a higher capacitance. This enhancement is attributed to stronger ion–carbon interactions and a higher concentration of topological defects like edge sites, which facilitate more efficient ion packing and charge compensation.^90^ This behavior further indicates that surface-controlled kinetics arise not only from geometric accessibility but also from favorable local interactions that enable rapid ion accommodation within confined environments. This finding underscores that a degree of structural disorder is beneficial for maximizing rapid surface interactions, ensuring that ion diffusion does not become the rate-limiting step in high-rate applications.

### 2.2 Diffusion-Controlled Kinetics

While surface adsorption is inherently fast, the overall rate capability of an EDLC can be limited by the diffusion of ions from the bulk electrolyte to the active surface sites within the electrode’s porous structure. Ion diffusion coefficients within carbon micropores are typically one to two orders of magnitude lower than in bulk electrolytes, potentially creating a kinetic bottleneck that slows charging.^77,85^ Therefore, a primary goal in designing high-rate porous carbons is to engineer shortened and efficient ion-diffusion pathways to minimize these transport limitations.

In addition to geometric constraints such as pore length and tortuosity, diffusion limitations in nanoporous carbons can also arise from coupled interfacial processes, including ion desolvation and ion exchange, which introduce additional kinetic barriers during the ion transport. Molecular simulations have shown that ion transport is highly dependent on the pore geometry, with diffusion being unexpectedly accelerated in slit-shaped pores under certain conditions.^92^ This highlights the critical importance of the pore architecture. A key strategy for enhancing ion transport is the design of hierarchical pore structures, where larger mesopores or macropores act as “ion highways”, rapidly delivering ions deep into the electrode structure, while smaller micropores provide the high surface area for charge storage. This interconnected network effectively reduces the diffusion distance that ions must travel through constricted pathways. Despite the inherent diffusion challenges in micropores, experimental studies on materials such as carbide-derived carbons (CDCs) have demonstrated that fast charging is achievable, with discharge times as low as 20 s. This is attributed to the low in-pore resistivities and efficient transport within their optimized microporous networks.^66^ This demonstrates that when pore structures are well connected and tortuosity is minimized, even predominantly microporous materials can sustain high power output.

It is important to distinguish this diffusion-limited capacitive process from that in pseudocapacitors, which store charge via fast Faradaic redox reactions (Figure 3).^96^ Although pseudocapacitive materials such as metal oxides can offer a higher energy density, they often face more severe diffusion limitations. In contrast, the focus for high-power EDLCs remains on optimizing the porous architecture to ensure that the electrolyte ions have rapid and unobstructed access to the vast internal surface area, thereby keeping the charge-storage process predominantly surface-controlled even at high rates. In practice, the balance between surface-controlled and diffusion-limited behavior depends on how effectively ion transport, interfacial processes, and pore structures are coordinated within the electrode.

**Figure 3:**
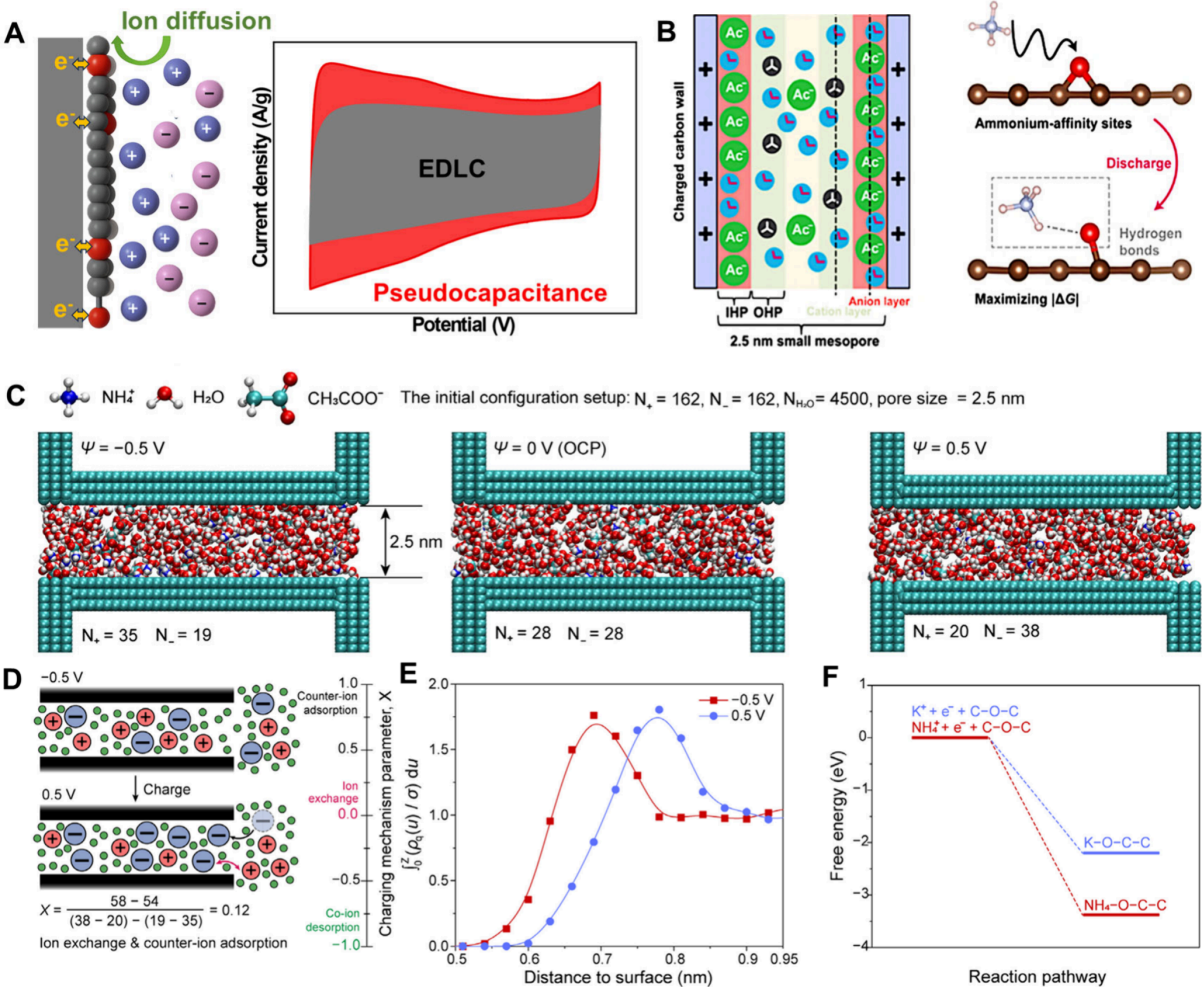
A) Mechanism of capacitance generation on a heteroatom doped carbon surface;^94^ Reproduced with permission from ref 94. Copyright 2023 American Chemical Society. B) Mechanism of capacitance generation in a mesopore of heteroatom doped carbon; C) distributions of H_2_O, NH_4_^+^, and Ac^–^ within the 2.5 nm carbon pore at different polarization potentials; D) schematic illustration of the proposed charge storage mechanism based on the MD simulations; E) integral of the charge density (ρq) of the ionic layer over the distance to the carbon surface (Z) and normalized by the carbon surface charges; F) Gibbs free energy change of the ring–opening reactions of epoxy group with the help of K^+^ and NH_4_^+^.^95^ Reproduced with permission from ref 95. Copyright 2025 Elsevier.

## 3 Material Challenges in High-Energy, High-Power Supercapacitors

The central goal in developing SPCs for advanced supercapacitors is to simultaneously achieve high energy density and high power density. This intrinsic tradeoff, which places supercapacitors between batteries and electrochemical capacitors in the energy–power landscape, is schematically illustrated in Figure 4A. This objective is fundamentally challenging because it requires reconciling conflicting material properties, from nanoporous architecture to macroscale electrode structure. The primary challenges can be grouped into interconnected areas concerning pore-level structural trade-offs, mechanistic conflicts between charge storage types, and practical limitations in fabrication and operation.

**Figure 4:**
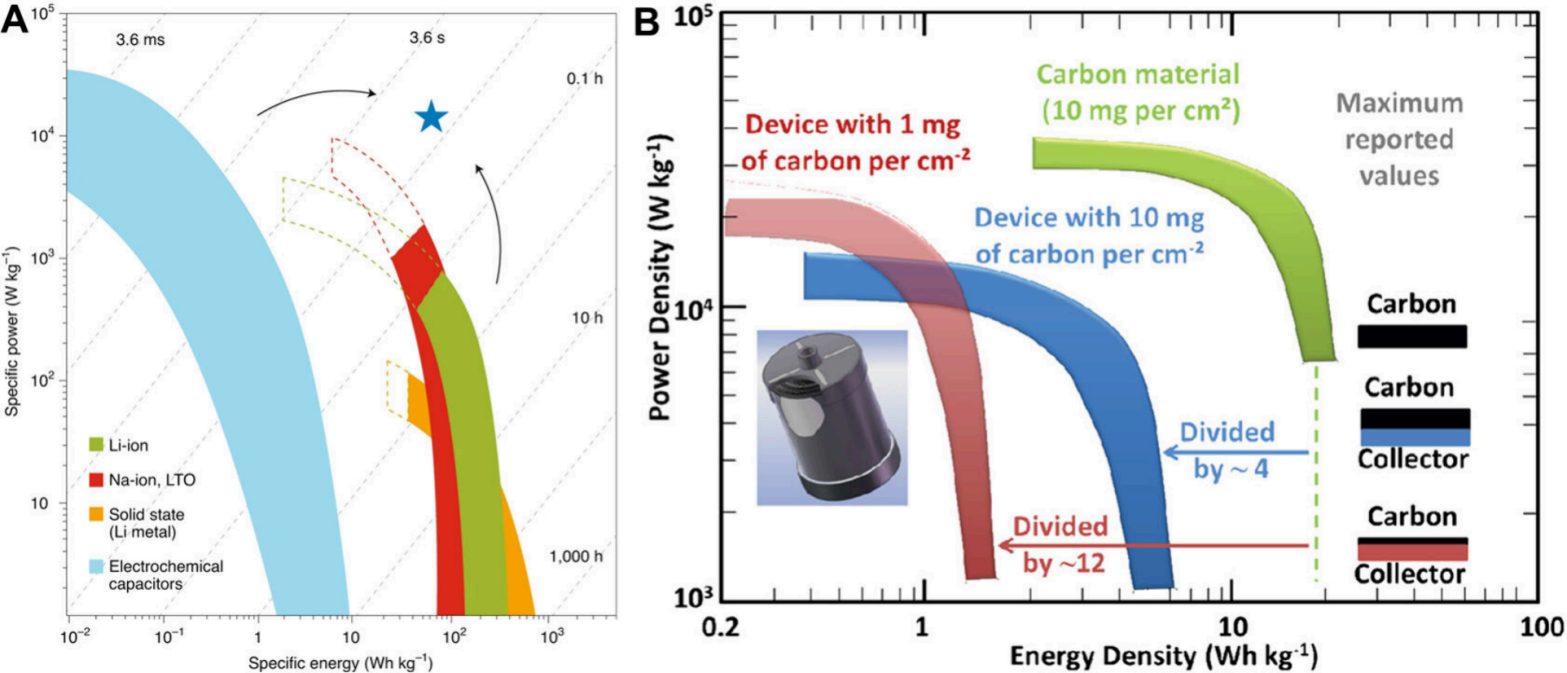
A) The plot shows the trends toward greater specific power for batteries and specific energy for electrochemical capacitors (arrows), blurring the boundaries between the two as the trends approach the star. Dashed lines represent zones where the cyclability of the device is altered in the case of symmetric cycling (same charging and discharging rate at 100% depth of discharge). For Li-ion batteries, Li plating at the negative electrode is mainly responsible for the decrease in cycle life and the limitation of charging rate. All the data for generating A) comes from commercial devices (datasheets or real tests). The data for solid state batteries come from industry roadmaps. The diagonal dotted lines and timescales represent characteristic operation timescales, obtained by dividing the energy by the power. LTO, lithium titanium oxide (Li_4_Ti_5_O_12_).^3^ Reproduced with permission from ref 3. Copyright 2023 Springer Nature. B) Ragone plots. Power vs energy density plots for the same electrochemical capacitors based on a gravimetric (per weight) basis. Calculations were performed using carbon density = 0.8 g cm^–3^, capacitance = 80–120 F g^–1^, 70 μm-thick Al current collectors, cell volume = 370 cm^3^, and cell voltage = 3 V.^97^ Reproduced with permission from ref 97. Copyright 2013 American Chemical Society.

These limitations become even more evident when transitioning from material-level metrics to device-level performance, as illustrated by the gravimetric Ragone plots in Figure 4B. However, it is important to note that such Ragone plots are typically based on gravimetric metrics derived from low-mass-loading electrodes (<2–3 mg cm^–2^), which can significantly overestimate practical device performance. In realistic supercapacitor configurations, performance is governed not only by intrinsic material properties but also by device-level parameters including areal capacitance (F cm^–2^), volumetric capacitance (F cm^–3^), electrode density, and electrolyte utilization. These factors collectively determine the true energy and power output at the system level.

In practical devices, electrodes are typically fabricated with mass loadings exceeding 10 mg cm^–2^ and thicknesses above 100 μm, where ion transport limitations, electrode tortuosity, and electronic resistance become dominant. Under such conditions, the gravimetric capacitance obtained at the material level often decreases substantially, while volumetric performance becomes the more relevant metric. For instance, highly porous carbons with low packing density may exhibit excellent gravimetric capacitance but suffer from poor volumetric energy density due to inefficient space utilization. This mismatch highlights that maximizing specific surface area does not necessarily translate into high energy density unless electrode packing and pore utilization are simultaneously optimized. Additionally, electrolyte quantity and distribution play a critical role in determining device performance. Excess electrolyte, commonly used in laboratory testing, can artificially enhance rate capability and capacitance by minimizing ion depletion effects, whereas practical devices operate under lean electrolyte conditions that impose stricter transport constraints. Similarly, full-cell configurations (e.g., symmetric vs asymmetric cells) introduce additional considerations such as voltage balancing, electrode matching, and internal resistance, which are not captured in half-cell or three-electrode measurements. Therefore, volumetric performance, rather than gravimetric capacitance alone, should be considered the primary metric for evaluating high-energy supercapacitors at the device level.

These device-level constraints further amplify the intrinsic material trade-offs and become critically acute when aiming for practical, commercial-level electrode mass loadings (e.g., >10 mg cm^–2^).^98^ In such thick electrodes, the multiple-fold increase in ion diffusion and electron transfer distances, coupled with a proportional increase in the amount of charge that must be stored and delivered per unit area within a given time, leads to severely hindered kinetics. The intrinsic tradeoff between energy and power begins at the pore level.^67,99–103^ High energy density requires maximizing the specific surface area (SSA) available for ion adsorption, a condition best met by a large volume of micropores (<2 nm). However, these narrow, often tortuous channels impose severe ion-transport resistance. Consequently, microporous carbons, even those with exceptional SSAs (>2000 m^2^ g^–1^), often suffer a 30–50% loss in capacitance when operated at practical charging rates (e.g., >1 A g^–1^).^104–106^ Conversely, high power density demands rapid, unimpeded ion transport, which is facilitated by mesopores (2–50 nm) and macropores (>50 nm) acting as “ion highways.” The compromise is that these larger pores contribute less to the overall SSA, thereby reducing the material’s volumetric and gravimetric energy density.^107^ While hierarchical pore architectures that combine micro- and mesopores are theoretically ideal, precise synthetic control remains a significant obstacle. More critically, not all measured surface area is electrochemically accessible: a substantial fraction can exist as “dead volume,” arising from disconnected, closed, or ultranarrow pores that ions cannot effectively access within practical charging timescales.^20,109^ Recent studies suggest that this limitation is not intrinsic but strongly dependent on pore connectivity and structural integration; for instance, engineering continuous ion-transport pathways and unblocked pore networks has been shown to significantly reduce dead volume and enhance volumetric energy density.^109^ Furthermore, the high SSA that enables high capacitance can also be a source of instability. The large electrode–electrolyte interface promotes parasitic reactions, especially during the first cycle (first-cycle irreversibility), and can lead to continuous electrolyte decomposition, limiting device lifetime. Agglomeration of nanomaterials during electrode fabrication can also reduce the accessible surface area and hinder ion transport, counteracting the benefits of nanostructuring.^110^

Beyond pore architecture and surface chemistry, the degree of graphitization also plays an important role in determining the electrochemical behavior of porous carbons. Increasing graphitic ordering generally improves electrical conductivity and facilitates rapid electron transport throughout the carbon framework, which is beneficial for high-rate charge–discharge processes.^111^ However, excessive graphitization can reduce the density of defects and edge sites and may decrease the accessible surface area, thereby limiting ion adsorption and electrochemical activity. Consequently, achieving an optimal balance between graphitic ordering and structural disorder is essential to simultaneously ensure efficient electron transport and sufficient ion accessibility. Recent studies have shown that strategies such as surface graphitization or defect-engineered graphitic carbons can partially overcome this conductivity–porosity tradeoff while maintaining high electrochemical performance.^111,112^

These structural trade-offs ultimately reflect a deeper mechanistic conflict between the two primary forms of charge storage. Electric double-layer capacitance (EDLC), the nonfaradaic adsorption of ions, is the source of a supercapacitor’s high power and exceptional cycling stability (>90% retention at >10 A g^–1^).^74,113–116^ However, EDLC is fundamentally limited by the physical constraints of ion packing, resulting in modest energy densities (e.g., 20–30 F g^–1^ in organic electrolytes).^117^ To overcome this energy limit, pseudocapacitance is introduced via Faradaic contributions, typically through heteroatom doping (e.g., N, O, S).^15^ Nitrogen doping, for instance, can increase capacitance by 30–50%.^118^ Nevertheless, these redox reactions are kinetically slower and introduce higher charge-transfer resistance. This gain in energy comes at the direct cost of power. For example, N-doped carbons may show higher capacitance but exhibit higher resistance.^119^

These fundamental challenges are further amplified when moving from idealized materials to practical, device-level implementation. To achieve high areal energy density, the key metric for practical systems, electrodes must typically be fabricated with substantial thickness (>100 μm). Such thick architectures markedly elongate and increase the tortuosity of ion-transport pathways, which can lead to significant losses in capacitance and rate performance under high current densities, particularly when electrode thickness reaches the tens-to-hundreds-micrometer regime.^11,120–122^ The magnitude of this performance decay depends strongly on pore architecture, electrolyte transport properties, and electrode design, rather than being an intrinsic limitation of thickness alone. This effect is critical because the performance metrics of thin-film electrodes, such as high gravimetric power density, do not scale linearly with increased electrode thickness. As a result, while a material may exhibit excellent gravimetric energy density at the nanoscale, its volumetric energy density at the device level, which is ultimately more relevant for most applications, can be disappointingly low.^97^ In these thick electrodes, ion depletion at high rates effectively suppresses the slower redox contributions, causing the device to revert to lower-energy, EDLC-dominated behavior.^121,123^ Furthermore, high-rate cycling generates significant localized heating, and temperatures exceeding ∼80 °C can accelerate structural and electrochemical degradation; as a result, devices often exhibit substantial capacity/capacitance fade over long-term cycling.^124–126^ Finally, many promising synthetic routes such as hard templating with silica or CaCO_3_, are difficult to scale commercially because they typically require corrosive etchants (e.g., HF, strong base) or energy-intensive template-removal steps, whereas softer templating approaches, while more benign, often give poorer structural uniformity and less precise pore control.^28,127^ Overcoming these interconnected challenges requires a shift from empirical optimization to integrated, predictive design. Emerging strategies are beginning to address these trade-offs, including the creation of three-dimensional conductive frameworks (e.g., graphene aerogels, CNT sponges, or 3D-printed carbon scaffolds) to reduce tortuosity and improve ion/electron transport,^8,128^ and advanced surface functionalization—for example, covalently grafting redox-active moieties onto carbonaceous scaffolds—to introduce Faradaic capacity while avoiding dissolution or the physical blocking of EDLC-active surface area.^129,130^ Computational and machine learning (ML) models also accelerate discovery by collecting and analyzing data to predict optimal architectures, demonstrating how structural features correlate with capacitance performance, and providing guidance for the design of favorable structures to enhance electrochemical properties.^32,131,132^ Looking ahead, progress will depend on a holistic approach that integrates materials synthesis, structural engineering, and predictive modeling, enabling the rational design of electrodes that simultaneously achieve high energy density, fast charge–discharge capability, and long-term stability.

From the above analysis, several key design principles emerge: (1) pore size matching: maximizing capacitance requires subnanometer pores comparable to ion size; (2) hierarchical porosity: mesopores/macropores are essential to sustain high-rate ion transport; (3) surface chemistry: heteroatom doping enhances the energy density but introduces kinetic penalties; (4) conductivity and network connectivity: critical for minimizing resistive losses in thick electrodes. Importantly, no single design parameter independently determines performance; rather, optimal materials require balancing these competing factors depending on the target application (high energy vs high power).

## 4 Structure Engineering in Synthetic Porous Carbons

Porous carbon materials have been extensively engineered to meet the demands of fast-charging energy storage, especially for applications requiring both high-rate capability and stable cycling. The pore structure, encompassing micropores, mesopores, macropores, and their combinations, plays a pivotal role in ion-transport kinetics and charge-storage mechanisms. Careful design of the pore architecture optimizes ion accessibility, reduces the diffusion resistance, and enhances electrolyte infiltration.

While gravimetric capacitance (F g^–1^) is widely reported in the literature, it often overestimates the practical energy storage capability of porous carbons, particularly under low mass loading conditions. For high-energy applications, volumetric metrics (F cm^–3^ and Wh L^–1^) are more directly relevant, as they incorporate both intrinsic capacitance and electrode packing density. Notably, many highly porous carbons exhibit low bulk density, which limits their volumetric performance despite high gravimetric capacitance. Therefore, a comprehensive evaluation of SPCs must simultaneously consider gravimetric and volumetric metrics to realistically assess their suitability for practical devices.

However, different structural design strategies contribute to electrochemical performance in fundamentally distinct ways, and their roles cannot be understood in isolation. To establish a unified perspective, the structure–mechanism–performance relationships across representative carbon architectures are summarized in Table 1. Rather than serving as a simple summary, this table provides a comparative framework that links pore structure, precursor strategy, electrolyte compatibility, and rate-limiting processes to practical performance metrics.

**Table 1:** Structure–Mechanism–Performance Correlation of Synthetic Carbon Architectures for Supercapacitors

Carbon Type	Precursor Class/Strategy	Energy Density	Power Capability	Rate-Limiting Mechanism	Key Insight	Mechanistic Role	Key References
Microporous (<2 nm)	Precursors: Phenolic resins, pitch, PAN fibers, MOFs (ZIF-8), PAFs/COFs/CMPs, carbides (SiC, TiC). Templates: Hard (zeolites Y, β), soft (F127, C_16_TMA^+^), self-templates (PAFs/COFs). Activation: KOH, ZnCl_2_, CO_2_, steam, Cl_2_	High	Low	Intraparticle diffusion in tortuous micropore networks; ion accessibility limitations	Surface area alone is insufficient; pore–ion size matching is critical	Primary EDLC storage sites; subnm confinement induces partial desolvation and anomalous capacitance	^28,67,66,68,85,87,133^
Mesoporous (2–50 nm)	Precursors: RF resin, phloroglucinol-based resins, dopamine, sucrose, polymers/resins, MOFs. Template: Soft (F127/P123), hard (SiO_2_, CaCO_3_, MgO, NaCl), dual/self-templating, MOF-derived. Carbonization (700–1000 °C), template removal (HF/HCl/water), mild KOH activation	Low to Moderate	High	Transition from intraparticle diffusion to interparticle transport and electrolyte resistance	Mesopores serve as ion buffering domains rather than primary storage sites	Facilitate ion transport and reduce diffusion resistance; enable fast access to micropores	^67,133–138^
Macroporous (>50 nm)	Precursors: Phenolic resins, sucrose, pitch, GO/graphene, CNT/graphene frameworks, gels. Templates: hard (SiO_2_, PS spheres), emulsion (HIPEs), freeze-casting, 3D printing, template-free (spray drying/gas foaming). Carbonization (800–1000 °C), template removal (HF/NaOH or solvent)	Low to moderate	High	Shifts to electrolyte conductivity and contact resistance	Macropores are transport infrastructure, not storage centers	Provide ion reservoirs and long-range transport pathways; reduce concentration gradients	^102,139–143^
Hierarchical carbon	Precursors: Zn-MOFs (IRMOF, ZIF-8), RF gels, PAN fibers, PVA/PANI/GO hydrogels. Templates: Self-templating (MOF pyrolysis, phase separation), dual templating (colloidal crystals + block copolymers), space-confined polymerization; KOH/CO_2_ activation or direct pyrolysis	High	High	Shifts to device-level limitations (separator, ionic conductivity, contacts)	Effective hierarchy requires connectivity, not just broad pore size distribution	Ensures ion supply to micropores under high-rate/high-loading conditions	^20,144–149^
Hollow carbon	Precursors: PDA, phenolic resins, polypyrrole, o-PD/glycine, 3-AP/F copolymers, PAA/PEI vesicles, melamine–F resins. Templates: Hard (SiO_2_, PS, CaCO_3_), emulsion/Pickering templating, self-, and dual templating (SiO_2_ + block copolymers), spray pyrolysis/Ostwald ripening; tunable shell thickness and cavity size; carbonization (700–1000 °C), template removal, optional KOH activation	Moderate	High	Shell diffusion and interparticle transport dominate	Hollow design enhances kinetics, not intrinsic storage density	Shortens ion diffusion length; cavity acts as local electrolyte reservoir	^150–155^
Heteroatom-doped carbon (N, O, S, P, B)	Precursors: PANI, PPy, melamine-based polymers, ionic liquids. Doping: In situ or postdoping (N, P, B, S, F), template-assisted pyrolysis (SiO_2_, ZnO, molten salts). Optional CO_2_/steam activation	High (EDL + Pseudo)	Moderate	Can shift to redox kinetics or side reactions at high potential	Doping tunes electronic structure and interfacial chemistry beyond adding redox sites	Introduces pseudocapacitance; modulates EDL structure, ion affinity, and conductivity	^15,31,41,95,156,157^
Carbonaceous networks	Precursors: CCl_4_, SiC, g-C_3_N_4_, C_6_Cl_6_, conjugated monomers, CNT/graphene precursors. Liquid alloy synthesis/dealloying (Na/K–CCl_4_, SiC/Ge), mechanochemical synthesis (ball milling), π-conjugated framework polymerization (CMPs, COFs, GDY), CVD growth, electrospinning, and sol–gel processing	Moderate-High	High	Electronic percolation and macroscopic ion transport	Networks provide conductivity rather than maximizing capacitance	Enable continuous electron pathways and reduced transport tortuosity; act as scaffolds	^135,158–162^

As shown in Table 1, microporous carbons primarily enhance the energy density through ion confinement and electric double-layer formation, whereas mesoporous and hollow structures facilitate ion transport by alleviating diffusion limitations. Macroporous frameworks function as ion-buffering reservoirs and long-range transport channels, particularly under high mass loading. Heteroatom doping introduces additional pseudocapacitive contributions but may compromise the rate capability due to slower redox kinetics. Meanwhile, graphitized carbons and synthetic carbonaceous networks improve electrical conductivity and enable efficient electron transport, while hierarchical architectures integrate multiple structural features to balance the energy and power performance.

These comparisons highlight that no single structural parameter independently determines electrochemical performance. Instead, optimal performance emerges from the coordinated interplay between pore architecture, surface chemistry, and electrolyte properties. Importantly, establishing such structure–property–performance relationships requires a high degree of structural predictability and controllability at the precursor level.

In this context, synthetic molecular and polymeric precursors offer a distinct advantage. Their well-defined chemical composition, tunable functional group distribution, and programmable polymerization or cross-linking pathways enable a more deterministic evolution of carbon frameworks during carbonization. This level of control allows pore architecture, heteroatom incorporation, and framework connectivity to be rationally designed and systematically correlated to electrochemical behavior.

By contrast, even highly processed biomass-derived precursors typically retain inherent compositional heterogeneity and undergo complex, less-predictable decomposition and rearrangement pathways during pyrolysis. As a result, it remains challenging to precisely control pore formation or to establish generalizable design rules linking precursor chemistry to final carbon structure and performance. Therefore, this Review focuses on SPCs as model systems to uncover mechanistically grounded correlations between carbon architecture and electrochemical performance, enabling rational material design.

Building on the framework established in Table 1, achieving high-energy, high-power supercapacitors requires the rational integration of multiple design principles, including pore size matching, hierarchical transport pathways, surface chemistry modulation, and conductive-network construction. The following section discusses these structural engineering strategies in detail with particular emphasis on their mechanistic roles and inherent trade-offs.

### 4.1 Pore Structure Engineering

The pore architecture of SPCs plays a pivotal role in determining their electrochemical performance, particularly under fast-charging conditions, where achieving a delicate balance between energy density and power density is essential. Rational pore design enables simultaneous enhancement of ion transport kinetics and energy density by optimizing the accessible surface area, ion diffusion pathways, and electrolyte wettability.^147,163–165^ Importantly, the pore-ion relationship extends beyond mere geometric accessibility. Recent studies reveal that in ionic mixture electrolytes, selective ion adsorption can occur based on the pore size. For instance, in a mixture of EMIM^+^ and TMA^+^ cations, smaller TMA^+^ ions can be selectively admitted into mesopores, where they pack densely with EMIM^+^, enhancing capacitance, while being largely excluded from micropores to preserve fast transport kinetics.^166^

More fundamentally, extensive experimental evidence demonstrates that capacitance does not scale linearly with specific surface area, but instead exhibits a strong and nonmonotonic dependence on pore size. In particular, systematic studies using well-defined model carbons reveal a pronounced increase in specific capacitance normalized by surface area when pore sizes approach the dimensions of electrolyte ions (Figure 5).^67^ This anomalous behavior, which contradicts classical solvated-ion adsorption models, arises from partial or complete ion desolvation under extreme nanoconfinement, effectively reducing the ion–electrode separation and enhancing charge storage efficiency.

**Figure 5:**
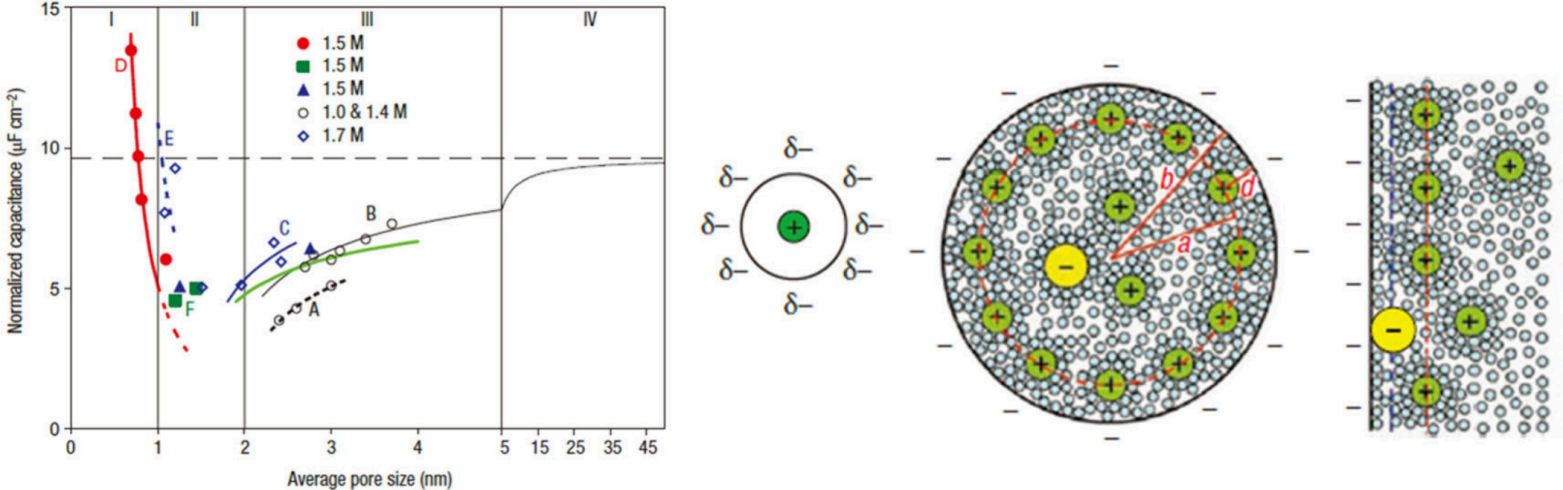
Specific capacitance normalized by SSA as a function of pore size for different carbon samples. All samples were tested in the same electrolyte (NEt_4_^+^, BF_4_^–^ in acetonitrile; concentrations are shown in the key). Symbols show experimental data for CDCs, templated mesoporous carbons and activated carbons, and lines show model fits. A huge normalized capacitance increase is observed for microporous carbons with the smallest pore size in zone I, which would not be expected in the traditional view. The partial or complete loss of the solvation shell explains this anomalous behavior. As schematics show, zones I and II can be modeled as an electric wire-in-cylinder capacitor, an electric double-cylinder capacitor should be considered for zone III, and the commonly used planar electric double layer capacitor can be considered for larger pores, when the curvature/size effect becomes negligible (zone IV). A, B: templated mesoporous carbons; C: activated mesoporous carbon; D, F: microporous CDC; E: microporous activated carbon.^67^ Reproduced with permission from ref 67. Copyright 2008 Springer Nature.

Critically, pore size must match electrolyte ion dimensions to maximize volumetric energy density. A narrow pore-size distribution centered around the ion size enables optimal pore volume utilization and enhances gravimetric capacitance.^167^ As highlighted by Figure 5, distinct pore-size regimes can be described by different electrostatic models, evolving from planar electric double-layer behavior in large pores to confinement-dominated charge storage in subnanometer micropores. Under high-rate operation (>5 A g^–1^), traditional carbon materials with randomly distributed or poorly controlled porosity often suffer from pronounced capacitance losses, primarily due to sluggish ion transport and limited accessibility of internal surface area. However, the extent of this degradation is not universal and depends strongly on pore connectivity, electrolyte properties, and the degree of structural optimization.^164^ Therefore, achieving a rational integration of micropores, mesopores, and macropores, together with appropriate electrolyte selection, is essential to ensure efficient ion transport and full utilization of active surface area, thereby maintaining high-rate performance without compromising energy density.^146,168^

Synthetic polymer-derived carbons offer a highly tunable platform for designing multiscale pore networks through chemical modification, templating strategies, and controlled thermal treatments. Micropores (<2 nm) are indispensable for maximizing electric double-layer capacitance (EDLC) by providing abundant ion adsorption sites.^66,169^ Subnanometer pores (<1 nm), in particular, have been shown to deliver disproportionately high capacitance per unit surface area due to ion desolvation effects, as quantitatively evidenced by the pore-size-dependent trends in Figure 5.^67,167^ Mesopores (2–50 nm) function as rapid ion transport channels, dramatically shortening diffusion lengths and facilitating electrolyte access to the microporous domains.^170^ Meanwhile, hollow structures and interconnected macropores (>50 nm) can further reduce ion transport resistance, promote electrolyte infiltration, and alleviate polarization, particularly in thick, high-mass-loading electrodes.^122,171^

Among the wide range of pore architectures accessible in SPCs, several representative and functionally important structural motifs—including microporous, mesoporous, and macroporous carbons defined by pore size, hierarchically porous carbons integrating multiple pore regimes, as well as hollow and hyperporous carbons arising from structural engineering and activation strategies—have garnered particular interest due to their tunable architectures and promising capabilities (Figure 6). Engineering the interplay among these pore regimes in a synergistic manner remains one of the foremost challenges and opportunities for optimizing SPCs tailored to fast-charging applications.

**Figure 6:**
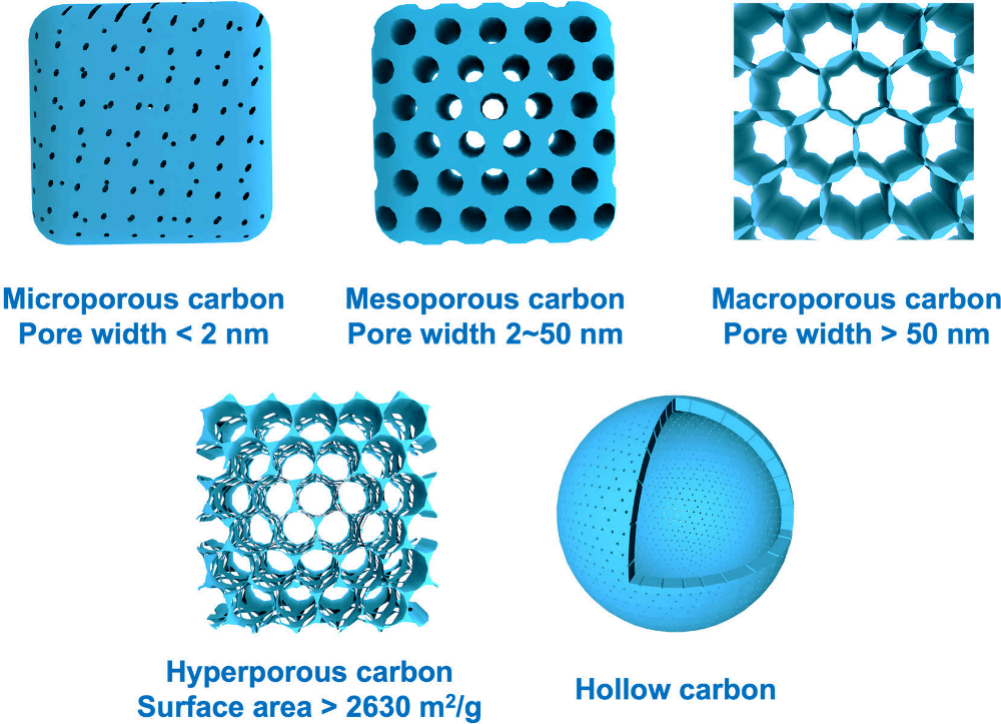
Illustration of typical porous carbon materials with different pore structures.

In the following subsections, we systematically review the design and functional roles of these major pore architectures in SPCs, including microporous, mesoporous, macroporous, hierarchically porous, hollow, and hyperporous carbons. We discuss their respective structural characteristics, advantages, limitations, and emerging approaches for precise pore engineering. Through this comprehensive analysis, we aim to provide insights into how advanced pore architecture designs can address the intrinsic energy–power trade-offs and enable the next generation of SPCs for high-performance, fast-charging energy storage devices.

#### 4.1.1 Microporous Carbon

Microporous carbons, characterized by pore widths smaller than 2 nm, are foundational materials for high-performance SPCs aimed at high energy storage. Structurally, these materials offer high specific surface areas, typically exceeding several hundred m^2^ g^–1^, with interconnected nanoscale pore networks that provide abundant ion adsorption sites critical for maximizing EDLC. The short diffusion lengths within microporous structures facilitate efficient charge storage, particularly beneficial for moderate-rate applications. The preparation of microporous carbons encompasses several major approaches, each imparting distinct structural characteristics.

##### 4.1.1.1 Hard-Template Strategy for Microporous Carbon Synthesis

The hard-template method is an efficient and precise approach for the fabrication of microporous carbon materials with well-defined micropores. Its core principle involves using structurally regular and thermally stable inorganic templates—such as zeolites, silicon carbide (SiC) or titanium carbide (TiC)—as spatial confinement frameworks to guide the deposition and carbonization of carbon precursors, thereby obtaining carbon frameworks that replicate the morphology of the template. This method is particularly well-suited for replicating the internal ordered microporous architecture of templates, enabling the production of carbon materials with high specific surface areas and controllable pore size distributions.

Building on this templating concept, the earliest and most representative example involves transforming carbides themselves into porous carbons. Carbide-derived carbons (CDCs) constitute a distinct class of microporous carbon materials synthesized through high-temperature chlorination of metal carbides such as SiC or TiC.^178–180^ During this process, selective removal of metal cations (e.g., Si^4+^ or Ti^4+^) as volatile metal chlorides leads to the formation of a carbon skeleton, which rearranges into a microporous structure. By tuning the carbide precursor and processing parameters, the micropore size can be precisely controlled within 0.6–1.1 nm. CDCs typically possess open porosities of 50–80% and specific surface areas up to 2000 m^2^ g^–1^. However, the collapse of the original carbide lattice after metal extraction often results in amorphous and structurally disordered carbon frameworks.^178,181^

To enhance the structural ordering and functionality of microporous carbon materials, recent efforts have focused on utilizing crystalline templates. Zeolites, in particular, have emerged as ideal scaffolds due to their intrinsically ordered microporous architectures and diverse framework types. Zeolites not only offer precise spatial confinement for carbon precursors but also influence adsorption and carbonization behavior through their surface functionalities, electrostatic properties, and pore environments. Notably, Y-type, β-type, and EMC-2 zeolites possess well-defined micropore networks that can be faithfully replicated in the resulting carbon structures. Furthermore, the physicochemical properties of zeolites—such as hydrophilicity/hydrophobicity, acidity/basicity, and ion-exchange capacity—can be tailored via post-treatment techniques, including ion exchange or silanization, which aid in the selective introduction and spatial distribution of carbon precursors within the pore channels.^182–184^ Zeolites also exhibit excellent thermal stability, maintaining their structural integrity during carbonization at elevated temperatures (700–1000 °C), thereby preserving the fidelity of the templated pore architecture.

Traditional zeolite-templated synthesis often adopts a “two-step” strategy: small molecular precursors (e.g., furfuryl alcohol, acetonitrile) are first impregnated into the zeolite pores, followed by high-temperature treatment and/or chemical vapor deposition (CVD) to further supply carbon sources and induce carbonization. For instance, Kyotani et al. introduced furfuryl alcohol into Y-type zeolite pores and then applied CVD to obtain ordered microporous carbon with a specific surface area up to 1910 m^2^ g^–1^, faithfully retaining the long-range order of the zeolite template.^185^ Ma et al. first formed a composite with furfuryl alcohol in Y-type zeolite, followed by thermal treatment at 700 °C and secondary CVD with propylene at 900 °C (Figure 7A), achieving microporous carbon with an exceptional surface area of 3600 m^2^ g^–1^ and a highly ordered structure confirmed by TEM.^172^ To improve carbon deposition efficiency and the density of the final product, Hou et al. developed a hot-press-assisted CVD strategy in a Y-zeolite system, adjusting the pressure during CVD to increase the packing density of microporous carbon to 0.9 g·cm^–3^ and finely tune the pore size within 0.7–1.0 nm (Figure 7B). The choice of carbon source also significantly impacts the resulting carbon structure.^173^ Thomas et al. prepared microporous carbon using liquid impregnation of furfuryl alcohol followed by CVD of propylene or acetonitrile in octahedral zeolite templates (Figure 7C). They found that propylene yielded more ideal pore structures and that in situ heat treatment was crucial for enhancing microporous ordering.^174^ Fabrice O. M. et al. synthesized microporous carbon using EMC-2 zeolite and retained the zeolite’s hexagonal order, achieving a highly ordered porous carbon structure.^26^

**Figure 7:**
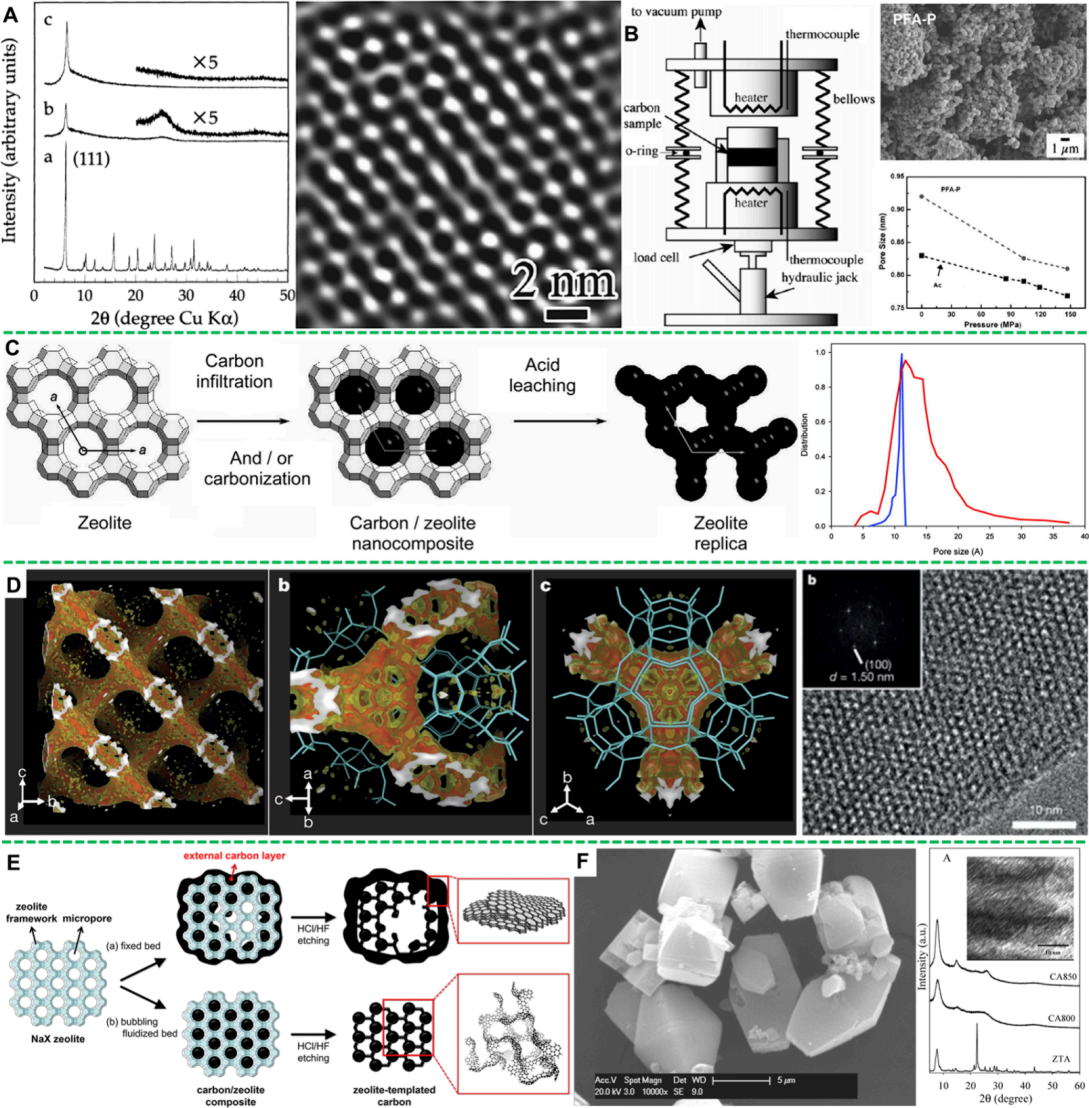
A) XRD and HRTEM image of microporous carbon prepared using zeolite Y as the template.^172^ Reproduced with permission from ref 172. Copyright 2001 American Chemical Society. B) Apparatus for the hot-pressing of carbon powder, SEM image, and N_2_ adsorption isotherms of microporous carbon prepared using zeolite Y as the template.^173^ Reproduced with permission from ref 173. Copyright 2007 Elsevier. C) The synthesis process of zeolite template and pore size.^174^ Reproduced with permission from ref 174. Copyright 2007 American Chemical Society. D) Lanthanum-catalyzed synthesis of microporous 3D graphene-like carbons in a zeolite template.^175^ Reproduced with permission from ref 175. Copyright 2016 Springer Nature. E) Scalable synthesis of zeolite-templated ordered microporous carbons without external carbon deposition.^176^ Reproduced with permission from ref 176. Copyright 2020 Elsevier. F) High surface area zeolite-like carbon materials.^177^ Reproduced with permission from ref 177. Copyright 2007 American Chemical Society.

Despite the advances in structural replication, traditional high-temperature CVD often leads to nonselective carbon deposition on the external surfaces of the template, resulting in disordered coke or graphitic carbon that hinders micropore accessibility and mass transport. To overcome this issue, Ryoo et al. innovatively introduced La^3+^ ions into NaY zeolites via partial ion exchange to enhance pore acidity and lower the carbon deposition temperature (Figure 7D). Under identical conditions, the carbon deposition rate within La-Y zeolite was 20 times that of HY-type zeolite, greatly improving selective internal deposition while avoiding external accumulation and yielding a structured graphene-like carbon framework.^175^ Similarly, Zhao et al. applied silylation pretreatment to Y-type zeolite to improve precursor permeability and achieved high nitrogen doping levels (up to 5.2 at.%) through acetonitrile CVD while maintaining structural order.^186^ In addition to modifying templates, tuning synthetic conditions can also suppress external carbon deposition. Choi et al. realized large-scale synthesis (>80 g) of zeolite-templated carbon in a bubbling fluidized bed (Figure 7E), avoiding external carbon formation and significantly enhancing electrolyte diffusion compared to conventional fixed-bed systems. This method shortened synthesis time and yielded microporous carbon with well-defined order, large surface area (∼3000 m^2^ g^–1^), and high micropore volume (∼1.1 cm^3^·g^–1^), exhibiting excellent electric double-layer capacitance performance.^176^

To further simplify the synthesis process of microporous carbon, researchers have also explored more straightforward “one-step” strategies, such as direct CVD or direct impregnation, to replace the traditional multistep routes. Direct CVD typically involves simultaneous exposure of the carbon source and the template, yielding microporous carbon with high surface areas. Mokaya et al. used β-type zeolite as a hard template and acetonitrile as the sole carbon source to fabricate highly ordered microporous carbon via a one-step CVD process without prior impregnation (Figure 7F). The resulting material exhibited a surface area of 3200 m^2^ g^–1^ and a pore volume of 2.41 cm^3^ g^–1^, highlighting the efficiency of direct CVD in micropore formation and property enhancement.^177^ To further increase carbon deposition efficiency inside the pores, Ryoo et al. employed a cofeeding system of acetonitrile and water vapor to regulate gas diffusion and reaction behavior during CVD, enabling more uniform and deeper pore filling.^187^ In parallel, the direct impregnation method has garnered significant attention due to its operational simplicity and minimal equipment requirements. This method typically integrates precursor infiltration and in situ carbonization into a one-pot process. Su et al. utilized furfuryl alcohol as the precursor and NH_4_Y zeolite as the template, preparing microporous carbon via solution infiltration followed by direct thermal treatment. The process relies on the thermal decomposition of small-molecule precursors under spatial confinement of the template to form microporous networks. Experimental results showed that increasing the carbonization temperature further optimized the pore structure, with surface area and pore volume reaching 3683 m^2^ g^–1^ and 2.0 cm^3^ g^–1^, respectively, at 1100 °C.^188^ Notably, ammonium ions in the template acted not only as structural stabilizers but also as nitrogen sources during carbonization, enabling effective nitrogen doping and endowing the carbon framework with enhanced electronic properties and functionalization potential.

To overcome the limitations of small-molecule carbon precursors and broaden the functional applications of microporous carbon, ionic liquids have also been explored as carbon sources. These materials offer advantages such as low vapor pressure, carbonizability, and tunable composition, and some can directly undergo high-temperature carbonization while incorporating heteroatoms. For example, Kyotani et al. used NaY zeolite as a template and a boron- and nitrogen-containing ionic liquid (EMIT) as the precursor to prepare B,N-codoped ordered microporous carbon, which exhibited high surface area and enhanced electrochemical properties, promoting its application potential in energy storage and catalysis.^189^

Although the hard-templating strategy enables the synthesis of microporous carbon materials with exceptionally high specific surface areas, the template removal process typically relies on toxic hydrofluoric acid (HF), which is time-consuming and hazardous. To circumvent the use of HF, Rao et al. proposed an NH_4_F-assisted approach, where the SBA-15 template was successfully removed by high-temperature calcination under an argon atmosphere.^190^ However, this method still requires harsh thermal treatment and may result in fluorine residues or gas emissions, complicating the overall process and limiting its scalability for practical applications.

More broadly, despite the precise structural control offered by hard-templating approaches, their practical implementation is often hindered by multistep processing, high energy consumption, and the use of costly or hazardous reagents (e.g., zeolite templates, CVD precursors, or corrosive etchants such as HF). These factors not only increase production cost but also reduce yield and scalability, raising concerns regarding their economic feasibility for large-scale manufacturing and practical electrode fabrication. In particular, the combination of expensive precursors, low throughput, and energy-intensive processing makes these precision synthesis routes difficult to justify for commercial-grade electrodes requiring high mass loading and cost efficiency. In contrast, commercial activated carbons—typically derived from biomass or low-cost precursors via chemical or physical activation—provide a far more economical and scalable alternative, albeit with less precise control over pore architecture.

Therefore, while templating strategies offer superior control at the material level, their translation to industrial applications remains constrained by cost–performance trade-offs and process complexity. In light of these limitations, increasing attention has been directed toward soft-templating strategies, which aim to balance structural tunability with synthetic simplicity and scalability.

##### 4.1.1.2 Resin Derived Microporous Carbon

Thermosetting resins such as phenolic resin have emerged as an effective precursor to generate microporous carbons with avoidance of corrosive etchants. These methods rely on the self-assembly of polymers, surfactants, or metal ions to guide pore formation during carbonization, thus eliminating toxic steps such as HF etching and offering a more sustainable route for microporous carbon synthesis. For instance, Huo et al. developed a cooperative templating method using the cationic surfactant C_16_TMA^+^, which, through controlled self-assembly with phenolic resin precursors, allowed for uniform coating on various functional core materials. This strategy yielded core–shell or hollow carbon spheres with wormlike micropores (∼0.6 nm), uniform particle size distribution, and tunable shell thickness and pore diameter.^194^

Building on this ability to control nanosphere morphology, efforts have been made to construct multifunctional microporous carbons. Matyjaszewski et al. introduced a “hypercrosslinking plus surface-initiated radical polymerization” strategy to fabricate “hairy” polymer nanospheres comprising a microporous core and a hydrophilic brush-like shell. The resulting structure maintained its nanoscale morphology while being convertible into microporous carbon with enhanced water dispersibility and environmental responsiveness.^195^ Similarly, Li et al. employed the nonionic triblock copolymer F-127 as a soft template and developed an improved Stöber method (Figure 8A) to synthesize nanospheres as small as 50 nm. After CO_2_ or KOH activation, the materials exhibited abundant micropores and high surface areas (up to 3259 m^2^ g^–1^ with CO_2_ activation), forming a hierarchical micro/mesoporous structure that facilitated ion transport and endowed the carbon with excellent supercapacitive performance.^191^

**Figure 8:**
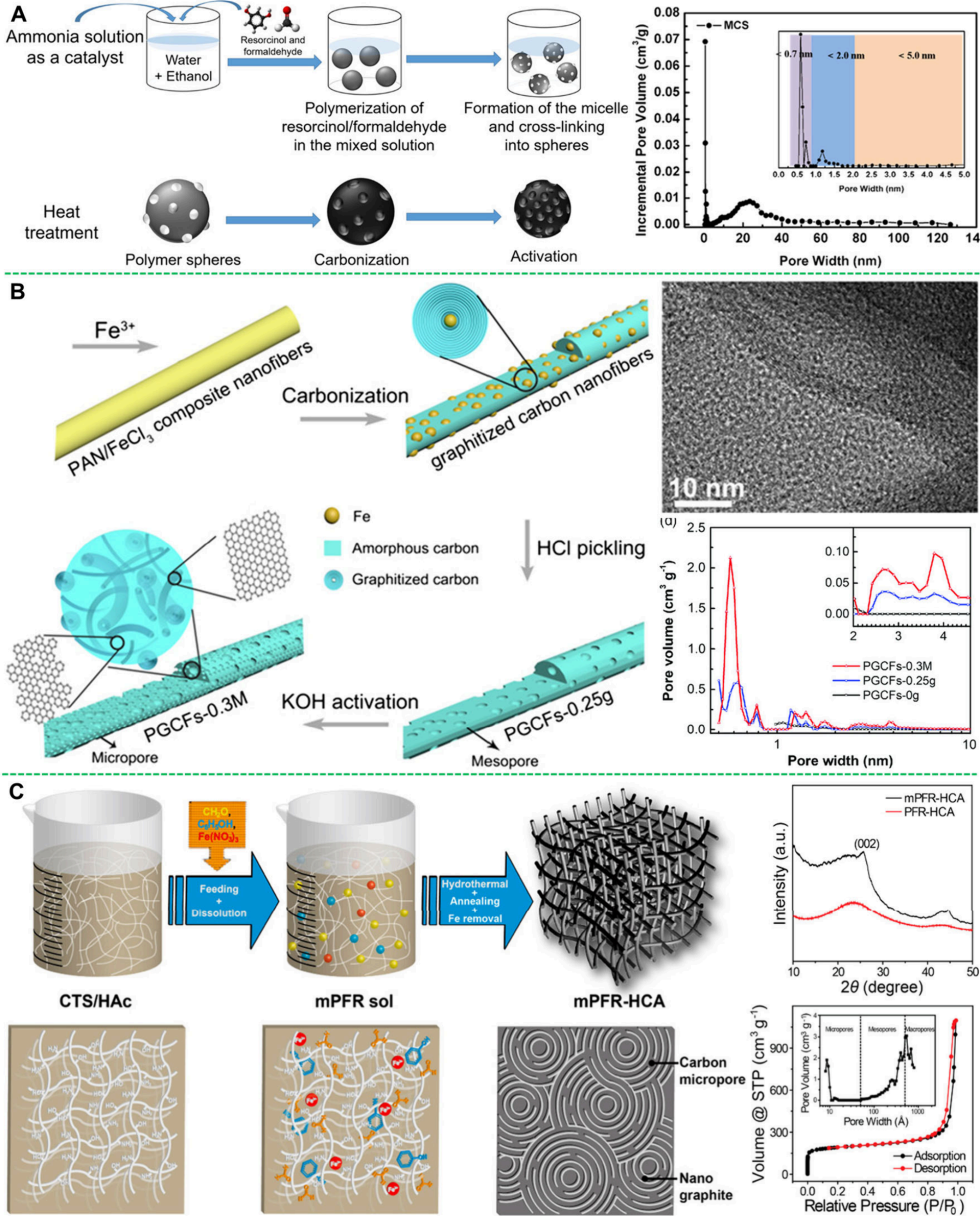
A) Preparation and activation of microporous carbon nanospheres.^191^ Reproduced with permission from ref 191. Copyright 2020 American Chemical Society. B) The synthesis of porous graphitic carbon fibers (PGCFs).^192^ Reproduced with permission from ref 192. Copyright 2020 Wiley. C) Ion-catalyzed synthesis of microporous hard carbon embedded with expanded nanographite.^193^ Reproduced with permission from ref 193. Copyright 2016 American Chemical Society.

As the demand for precise control over microporous structures increases, researchers have advanced from traditional surfactant- or block-copolymer-directed assembly to “soft-template + catalytic synergy” strategies guided by metal ions. A representative case is the Fe^3+^-chelation-assisted method, where metal ions coordinate with carbon precursors to regulate both graphitization and pore development at the molecular level. Pang et al. introduced FeCl_3_ as a catalyst in electrospun PAN/Fe^3+^ composite fibers, yielding microporous carbon fibers (PGCFs) with a graphitic framework and combined micro/mesoporosity after carbonization (Figure 8B). The Fe^3+^ ions catalyzed graphitization at reduced temperatures (∼800 °C), while subsequent KOH activation introduced micropores and retained mesopores etched by Fe nanoparticles, thus achieving synergistic enhancement of electronic conductivity and ion diffusion. This resulted in remarkable rate performance, maintaining 165 F·g^–1^ even at 300 A·g^–1^.^192^ However, despite the excellent gravimetric rate capability, such highly porous and electrospun fiber-based architectures typically exhibit relatively low packing density, which may limit their volumetric capacitance under practical conditions.

In a similar fashion, Yu et al. employed an Fe^3+^-chelation soft-templating strategy (Figure 8C), where Fe^3+^ was uniformly incorporated into a phenolic resin matrix. During carbonization, Fe nanoparticles formed in situ and catalyzed the generation of expanded graphite domains (with interlayer spacing ∼0.36–0.4 nm) and uniform micropores (∼0.8 nm), producing a hard carbon aerogel (mPFR-HCA) with high surface area (664 m^2^·g^–1^) and elevated hydrogen content. The material delivered excellent rate capability and stability in lithium/sodium ion batteries, achieving an initial lithium storage capacity of 2391 mAh·g^–1^ and retaining 1180 mAh·g^–1^ after 200 cycles.^193^ Its volumetric energy density significantly outperformed traditional hard carbon, demonstrating the unique advantages of soft-templating for fine-tuning porous carbon for energy storage.

Despite these advances, challenges remain. The compatibility and self-assembly behavior between the template and carbon precursor can be sensitive to variables such as the solvent environment and temperature, leading to inconsistencies in pore structure and reproducibility. Additionally, certain soft templates still require high-temperature decomposition or auxiliary atmospheres, increasing the process complexity. Issues such as low carbon yields and limited structural stability also hinder practical deployment.

To further simplify the synthesis and enhance structural robustness and reproducibility, researchers have recently turned to a more straightforward and efficient approach: the self-templating strategy. This method does not rely on externally introduced templates; instead, it exploits intrinsic structural features or compositional gradients within the precursor to induce the in situ pore formation. Offering a simplified process, high carbon yield, and strong controllability, the self-templating strategy has rapidly gained traction as a promising avenue for microporous carbon research.

##### 4.1.1.3 Self-Templating Strategy for Microporous Carbon Materials

The self-templating strategy leverages precursors with intrinsic microporous structures that undergo carbonization during pyrolysis, thereby preserving or evolving into porous carbon frameworks. Common types of precursors include porous aromatic frameworks (PAFs), metal–organic frameworks (MOFs), microporous organic polymers (MOPs), covalent organic frameworks (COFs), conjugated microporous polymers (CMPs), and certain small organic molecules.

##### 4.1.1.3.1 Microporous Carbon Derived from Porous Aromatic Frameworks (PAFs)

PAFs are three-dimensional porous materials constructed by covalently linking rigid aromatic units, characterized by high cross-linking density and excellent thermal stability. The abundant C–C bonds in their structure provide ideal carbon sources. Zhang et al. proposed a cocarbonization strategy involving PAFs and furfuryl alcohol, where sulfonated PAF-1 served as catalytic sites to guide the polymerization of furfuryl alcohol within the pores (Figure 9A). The resulting microporous carbon exhibited high surface area and uniform pore size distribution. Comparative studies revealed that furfuryl alcohol introduction not only enhanced the specific surface area but also optimized pore architecture.^196^ Ben et al. further demonstrated the significant impact of carbonization temperature on the structure; a surface area of 1191 m^2^·g^–1^ was achieved at 450 °C, whereas higher temperatures led to pore collapse and surface area reduction, highlighting the importance of process control.^197^

**Figure 9:**
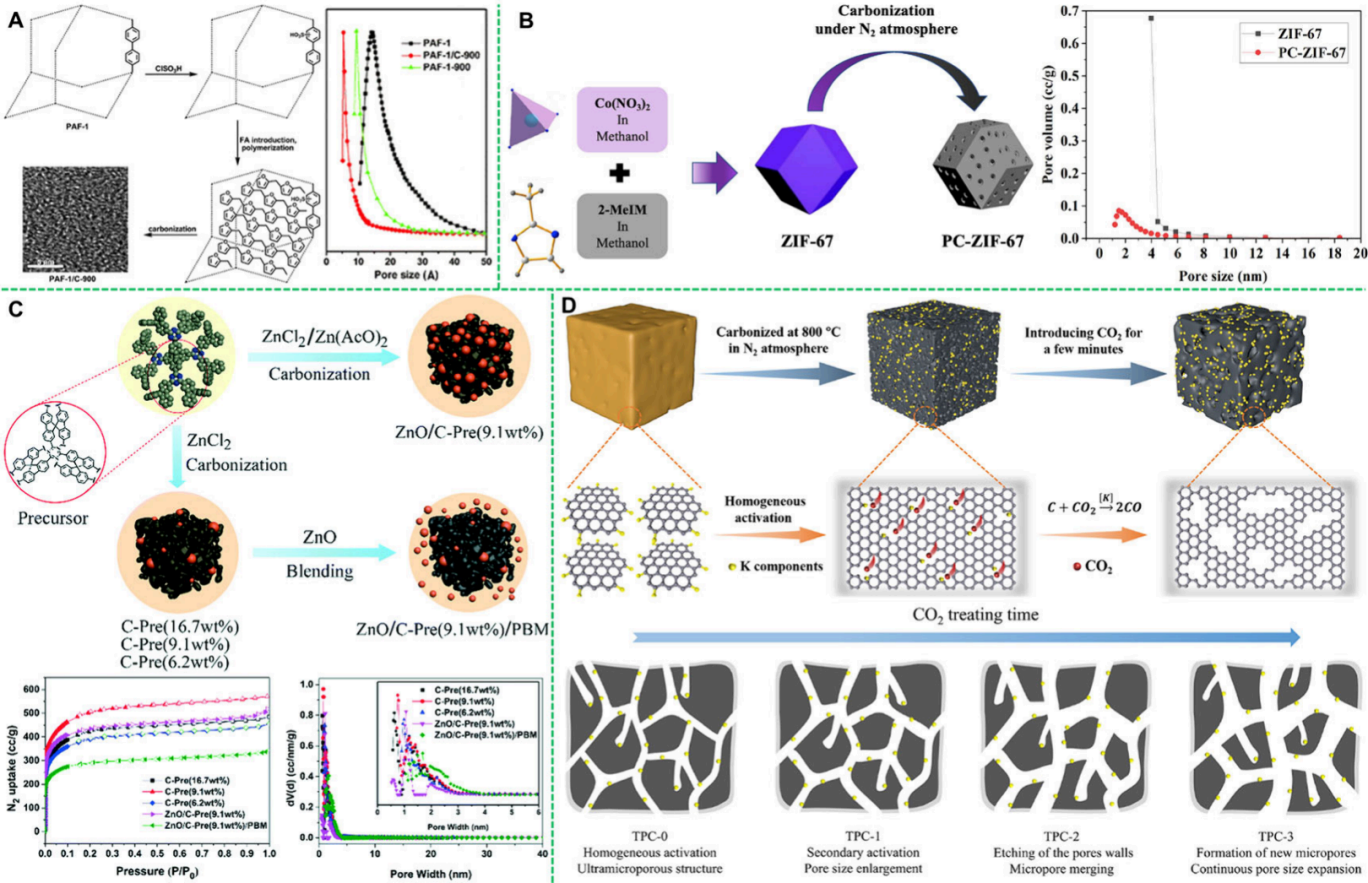
A) Microporous carbon material synthesized via thermolysis of a porous aromatic framework embedded with an extra carbon source.^196^ Reproduced with permission from ref 196. Copyright 2013 Royal Society of Chemistry. B) Microporous carbon derived from zeolitic imidazolate framework-67.^198^ Reproduced with permission from ref 198. Copyright 2023 Elsevier. C) Porous carbons from microporous organic polymers.^199^ Reproduced with permission from ref 199. Copyright 2019 Royal Society of Chemistry. D) Ultrafast pore tailoring of carbon by brief CO_2_ postactivation.^200^ Reproduced with permission from ref 200. Copyright 2022 Elsevier.

##### 4.1.1.3.2 Microporous Carbon Derived from Metal–Organic Frameworks (MOFs)

MOFs are crystalline materials composed of metal nodes and organic linkers, featuring high surface area, regular porosity, and versatile compositional tunability—ideal for self-templating precursors. Carbonization typically proceeds under inert atmospheres, yielding carbon materials that retain metal distribution and microporous structure. For instance, direct pyrolysis of ZIF-67 produces truncated cubic microporous carbon with a surface area up to 532 m^2^·g^–1^ (Figure 9B).^198^ Additionally, Khan et al. demonstrated that cocarbonization of Zn-based MOFs with furfuryl alcohol improved yield.^201^ It was found that increasing the carbonization temperature enhanced the surface area but also induced a transition from primarily micropores to a hierarchical porous structure comprising micro-, meso-, and macropores.

Beyond structural evolution, MOF-derived carbons often exhibit pronounced in situ graphitization behavior, which strongly depends on the nature of the metal nodes. Transition metals such as Fe, Co, and Ni can catalyze the transformation of amorphous carbon into graphitic domains during pyrolysis via a dissolution–precipitation mechanism, thereby enhancing electrical conductivity. In contrast, Zn-based MOFs (e.g., ZIF-8) typically yield predominantly amorphous carbons due to Zn evaporation at elevated temperatures, resulting in limited graphitization.

Another critical factor is the efficiency of the metal removal after carbonization. Residual metal species or nanoparticles are often retained within the carbon matrix, depending on the volatility of the metal and post-treatment conditions. Effective removal typically relies on strategies such as acid etching or oxidative treatments; however, strongly encapsulated or chemically bonded metal species can be difficult to eliminate completely, leading to an incomplete removal efficiency. While these species can be beneficial for catalytic applications, they may introduce drawbacks in electrochemical energy storage systems. For instance, residual metals can promote parasitic redox reactions, acting as redox-active centers or electron-conducting pathways that increase leakage current, leading to an increased self-discharge behavior. Moreover, metal-induced local structural heterogeneity may compromise long-term stability during repeated cycling.

Therefore, careful regulation of metal species—either through complete removal (e.g., acid etching) or controlled stabilization—is essential to balance conductivity enhancement and electrochemical stability in MOF-derived carbons.

##### 4.1.1.3.3 Microporous Carbon Derived from Microporous Organic Polymers (MOPs)

MOPs are covalent network polymers with highly controllable architectures, offering high surface area, regular pore channels, and low framework density. Rational design of monomers (e.g., triazine units, aromatic rings) allows not only the formation of microporous carbon but also the integration of functional metal sites. Gu et al. reported the thermal cotreatment of metal salts with triazine-based MOPs (Figure 9C), resulting in Zn-doped microporous carbon, where N-metal interactions endowed the material with potential catalytic activity.^199^ Additionally, hypercrosslinked polymers synthesized via Friedel–Crafts reactions^199,202,203^ or Schiff-base condensation^204,205^ also serve as effective carbon sources.

##### 4.1.1.3.4 Microporous Carbon Derived from Covalent Organic Frameworks (COFs)

COFs are porous materials formed via covalent bonding of organic units into 2D or 3D frameworks. Their well-defined framework provides a structural basis for ordered carbon skeletons upon carbonization, theoretically favoring graphitization and pore retention. Shiraki et al. prepared N-doped microporous carbon by direct carbonization of imide-linked COFs, where higher crystallinity facilitated graphitic carbon formation. However, structural collapse during pyrolysis often leads to surface area reduction.^206^ To address this, Jiang et al. developed a “template polymerization” approach by coating imine-type COFs on polyphosphazene spheres before carbonization.^207^ This method enhanced morphological stability and functional retention of the resulting microporous carbon, and proved applicable to various COF types, offering a generalizable route toward structurally controllable microporous carbons.

##### 4.1.1.3.5 Microporous Carbon Derived from Conjugated Microporous Polymers (CMPs)

CMPs, as self-templated organic frameworks, combine tunable microporosity with conductive skeletons. Ahmed et al. synthesized CMPs via Suzuki coupling of benzodithiophene-dione (DTDO) and bisfluorenyl (BF) units, achieving pore size control in the range of 1.8–2.14 nm and introducing pseudocapacitive sites. The resultant carbon delivered a high specific capacitance of 288.8 F·g^–1^ and excellent cycling stability in supercapacitors.^208^ This molecular-level structural control illustrates the distinct advantages of self-templating strategies for “structure-function integration.” However, despite the high gravimetric capacitance, CMP-derived carbons typically possess relatively low packing density due to their highly porous and rigid network structures, which may limit their volumetric capacitance in practical devices. In particular, the presence of abundant micropores and open frameworks can lead to inefficient space utilization if not properly densified. Therefore, while molecular-level structural control offers clear advantages for tuning pore architecture and functionality, its effectiveness for high-energy applications must be critically evaluated in terms of volumetric performance (e.g., F·cm^–3^ or Wh·L^–1^), rather than gravimetric metrics alone.

##### 4.1.1.3.6 Microporous Carbon from Pyrolysis of Carbon-Rich Small Molecules

Besides polymeric precursors, some carbon-rich small molecules can be directly pyrolyzed into microporous carbon. For example, Lu et al. achieved microporous carbon with a surface area of 1126 m^2^·g^–1^ via one-step carbonization of sodium ascorbate.^209^ Li et al. combined KOH activation with CO_2_ etching, accomplishing precise pore tuning (from 0.5 to 1.14 nm) within just 2 min (Figure 9D).^200^ The resulting carbon exhibited both high density (0.68 g·cm^–3^) and high surface area (2147 m^2^·g^–1^), significantly boosting supercapacitor performance. Moreover, Enis et al. employed thermal condensation of sulfur-containing oligomers to obtain S-doped microporous carbon with tunable sodium storage behavior.^210^

The self-templating approach offers a facile route to microporous carbon materials. By tailoring the precursor framework and controlling the carbonization kinetics, spontaneous pore evolution, morphology retention, and heteroatom doping can be simultaneously achieved. This provides a powerful platform for synthesizing microporous carbons with a high surface area, high doping levels, and good conductivity. However, challenges such as a broad pore size distribution, structural disorder, and limited precision in pore tuning remain. Future efforts should focus on advanced precursor design, in-depth control of carbonization dynamics, and synergistic component guidance to push forward their practical applications in energy storage and electrocatalysis.

##### 4.1.1.4 Micropores for Fast-Charging

Microporous structures (<2 nm) have been widely recognized as a powerful structural motif for improving the electrochemical performance of carbon-based electrodes, particularly in enhancing both energy and power densities of supercapacitors. The incorporation of micropores increases the specific surface area and provides abundant electroactive sites, thereby facilitating charge accumulation through electric double-layer formation, surface adsorption, and pseudocapacitive reactions. These attributes enable microporous carbons to deliver high specific capacitances, rapid charge/discharge behavior, and excellent cycling stability. For instance, zeolite-templated microporous carbons exhibit specific capacitances up to 300–500 F g^–1^ in aqueous H_2_SO_4_ electrolyte due to the presence of interconnected micropore channels that promote efficient ion adsorption.^211–213^ Chemical activation further optimizes surface area and pore accessibility. However, the benefits of microporosity are primarily reflected in gravimetric metrics, and their translation into volumetric energy density can be limited by factors such as pore accessibility, structural packing, and ion transport constraints in confined spaces. Li et al. tuned micropore sizes from 0.5 to 1.14 nm via CO_2_ activation (Figure 10), increasing the fraction of ion-accessible pores from 39% to 72%. The resulting carbon maintained a high volumetric capacitance of 70 F cm^–3^ even at a high current density of 20 A g^–1^, attributed to reduced diffusion resistance and improved pore connectivity.^200^ Nevertheless, excessive activation can distort micropore cages and lower accessibility, thus compromising both specific capacitance and power density. Therefore, fine control over micropore fraction, size distribution, and total pore volume is essential for balancing energy storage capacity with rapid ion transport.

**Figure 10:**
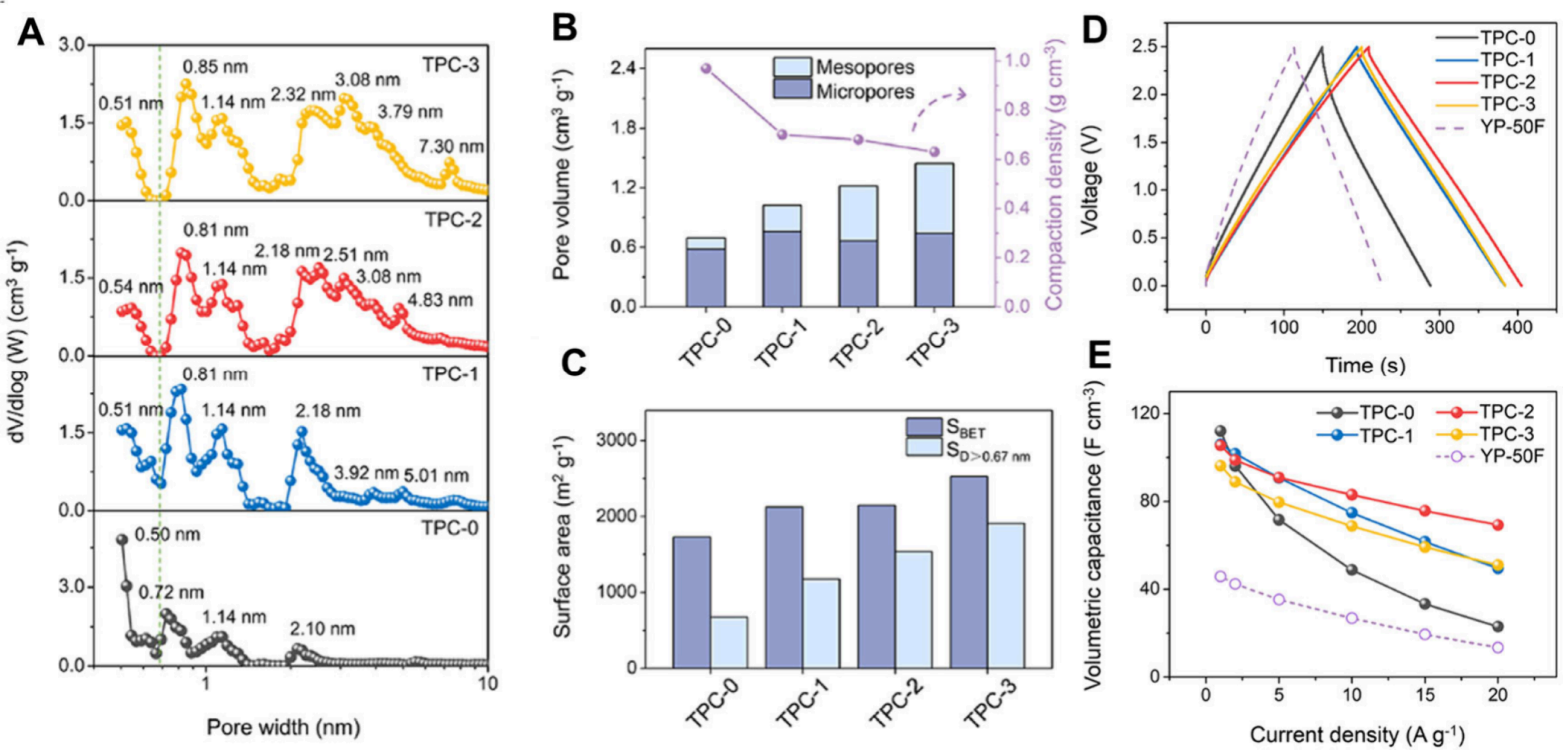
Structure and performance of tailored porous carbons: A) pore size distributions; B) porous structure evolution and compaction densities; C) surface areas (SBET) and potential surface areas (SD > 0.67 nm). Galvanostatic charge–discharge curves at D) 1 A g^–1^, E) volumetric capacitance.^200^ Reproduced with permission from ref 200. Copyright 2022 Elsevier.

Micropore size plays a decisive role in determining ion dynamics and capacitive behavior. When the pore diameter is comparable to the desolvated ion radius, fast ion transport channels are established with minimal diffusion resistance. Subnanometer pores (<1 nm) typically enhance gravimetric capacitance due to strong charge confinement, but overly narrow pores may hinder ion accessibility and significantly reduce material packing density. In contrast, slightly larger micropores (1–2 nm) achieve an optimal balance between charge density and diffusion kinetics.^214,215^ A positive correlation has been observed between the reciprocal of micropore diameter and gravimetric capacitance in carbide-derived carbons (CDCs).^66,67^ TiC-derived microporous carbon with ∼0.7 nm pores exhibited 160–190 F g^–1^ in H_2_SO_4_ at 5 mV s^–1^ within −0.5 to 0.5 V, and still maintained 140 F g^–1^ in acetonitrile electrolyte under high current densities.^66,216^ This behavior arises from partial or complete desolvation of ions (solvated radii ∼1.3 nm for cations, ∼1.16 nm for anions), which enables their penetration into size-confined micropores and efficient adsorption/desorption during charge–discharge. Crucially, while these high-porosity CDCs show impressive gravimetric metrics, their volumetric capacitance (F cm^–3^) must be scrutinized, as the tradeoff between specific surface area and skeletal density determines the ultimate energy density (Wh L^–1^) in compact devices.

Nitrogen doping offers an additional means to enhance charge storage performance. N-doped microporous carbons exhibit specific capacitances up to 340 F g^–1^ in aqueous H_2_SO_4_ under a 1.2 V cell voltage at 1 A g^–1^, owing to improved surface wettability and additional redox-active sites.^217^ However, this effect is less pronounced in organic electrolytes (e.g., TEA-BF_4_ /acetonitrile), where capacitance decreases to 50–120 F g^–1^ (at similar current densities and ∼2 V windows), highlighting the strong dependence of capacitive behavior on ion solvation and pore accessibility.^218^ The linear correlation between specific capacitance and accessible surface area observed in zeolite-templated carbons (ZTCs) further demonstrates that only effectively accessible micropores contribute to charge storage. Their practical application is often limited by excessive void space. Therefore, optimizing the volumetric capacitance (F cm^–3^) by balancing pore accessibility with electrode density is essential for maximizing the overall energy output in compact supercapacitors.

To overcome diffusion limitations intrinsic to purely microporous systems, hierarchical porous structures combining micro-, meso-, and macropores have been developed. In such architecture, micropores act as charge storage reservoirs, while meso- and macropores serve as mass transport highways. This structural synergy enables concurrent enhancement of capacitance and rate performance. For example, a graphitized micro–mesoporous carbon framework sustained 165 F g^–1^ at an ultrahigh current density of 300 A g^–1^, demonstrating outstanding rate capability.^192^ Therefore, rational engineering of micropore size and hierarchical connectivity—while maintaining sufficient packing density to ensure high volumetric performance (F/cm^3^ and Wh/L)—represents an effective approach for achieving both high energy and high power in compact supercapacitors.

Beyond supercapacitors, microporous carbons also play a crucial role in improving the electrochemical performance of battery electrodes. Micropores act as active sites for ion storage and as nanoscale reservoirs to buffer volume changes during cycling. For instance, Yu et al. synthesized Fe^3+^-catalyzed microporous phenolic resin–derived hard carbon (mPFR-HCA) with uniform ∼0.8 nm pores and expanded graphitic domains (0.36–0.4 nm). The integrated microporous–graphitic structure enhanced electrical conductivity and ion diffusion, enabling stable operation even at 5 A g^–1^ and delivering exceptional fast-charging capability. The abundant Li/Na storage sites resulted in an initial lithium capacity of 2391 mAh g^–1^—far exceeding that of conventional hard carbon.^219^

Moreover, microporous carbons derived from conjugated microporous polymers (CMPs) or metal–organic frameworks (MOFs) combine high porosity with structural regularity, yielding improved specific capacities and stable cycling. Micropores can also serve as confinement spaces for active species: for example, restricting sulfur monolayers within ∼0.7 nm channels enhance conductivity and suppresses polysulfide shuttling and irreversible Li_2_S deposition, achieving >80% capacity retention after 500 cycles.^220^ Similarly, a high-micropore-volume carbon (0.55 cm^3^ g^–1^, SC-1000) synthesized by high-temperature pyrolysis enables reversible Na^+^ filling and improved plateau capacity. Increasing pyrolysis temperature shifts the storage mechanism from surface-controlled to diffusion-controlled, while the stable microporous network promotes uniform sodium nucleation and reversible sodium metal deposition (>380 mAh g^–1^) with enhanced safety and cycle life.^210^

Micropores play a decisive role in establishing the capacity–rate synergy of porous carbons. Their abundant surface sites ensure high charge storage capability, while the short ion diffusion distance inside subnanometer pores enables rapid adsorption kinetics. However, excessive ultramicropores (<0.7 nm) may hinder ion accessibility, especially in solvated electrolytes. Achieving a balanced distribution of accessible micropores, coupled with mesoporous channels for ion buffering, is therefore essential to simultaneously sustain high capacity and fast charging performance.

Despite these advantages, the intrinsic tradeoff between the energy density and ion transport kinetics in microporous systems cannot be overlooked. Microporous carbons (pore size of <2 nm) are widely recognized as the primary contributors to energy density due to their high specific surface area and strong ion confinement effects. When the pore size approaches that of electrolyte ions, partial desolvation can reduce the charge separation distance, thereby enhancing the capacitance. However, this energy advantage is inherently accompanied by sluggish ion transport within ultramicropores, which becomes the rate-limiting step at high current densities and leads to a substantial capacitance decay. This limitation is particularly pronounced in thick electrodes or high-mass-loading systems that are relevant to practical applications.

Critically, microporosity alone is insufficient to achieve high-performance energy storage. Experimental comparisons consistently show that micropore-dominated materials exhibit superior capacitance at low rates but poor rate capability, highlighting a fundamental energy–power tradeoff. Therefore, micropores should be primarily regarded as charge storage sites rather than transport pathways. Their optimal utilization relies on precise pore size matching with electrolyte ions and, more importantly, on integration with meso- or macroporous transport networks.

##### 4.1.1.5 Challenges and Structural Optimization of Micropores

Despite the notable advantages of microporous structures in enhancing the performance of energy-storage devices, several critical technical challenges remain unresolved. From a materials perspective, the main limitations include the following:

First, regarding pore structure, ultramicropores (<0.7 nm) or closed micropores often hinder electrolyte infiltration, resulting in a “dead pore” effect. This impedes ion transport and leads to inaccessible capacity, especially under high-rate charge–discharge conditions where ions cannot efficiently access deep pores, drastically reducing the utilization of surface area. Second, in terms of performance trade-offs, materials with a purely microporous structure often face a conflict between porosity and packing density. Extremely high specific surface areas (>3000 m^2^/g) are typically accompanied by low bulk density, which in turn limits the achievable volumetric energy density. Despite their high gravimetric capacitance, microporous carbons often suffer from reduced volumetric performance when excessive porosity leads to low packing density, highlighting the need for density optimization. More critically, excessive microporosity can compromise mechanical integrity, increasing the risk of structural collapse during prolonged cycling and thereby shortening device lifespan.

From an electrochemical standpoint, restricted ion transport within narrow micropores leads to increased polarization and internal resistance, which adversely affect power performance. Moreover, purely microporous carbons often possess limited electrical conductivity due to insufficient graphitic ordering, requiring the addition of conductive additives that complicate electrode fabrication and reduce the overall energy density.

To address these issues, multidimensional structural optimization strategies have been proposed. First, pore size gradient engineering—through the construction of hierarchical micro–mesoporous architectures—allows for functional separation between < 2 nm micropores (for charge storage) and 2–50 nm mesopores (for ion transport), achieving a balance between capacity and rate performance. Second, nanostructuring strategies (e.g., carbon spheres with a 52 nm diameter) significantly shorten ion diffusion pathways and can reduce Warburg impedance by up to 40%. Third, chemical compositing approaches, such as atomic doping or the introduction of graphitic domains, improve electrical conductivity while preserving porosity. Fourth, synergistic optimization of porosity and packing density can be achieved through molecular-level precursor engineering or compression molding—for instance, CO_2_-activated mesoporous carbon spheres (MCS) maintain a high specific surface area of 3259 m^2^/g along with a packing density of 0.68 g/cm^3^.

Looking forward, future research on microporous carbons could be pursued along three directions: (1) precise regulation of subnanometer pores (0.5–1 nm) and elucidation of their ion sieving and transport mechanisms in electrolytes at the atomic scale; (2) development of self-supporting microporous electrode systems that eliminate the need for conductive additives, thereby simplifying electrode composition and enhancing overall energy density; and (3) integration of artificial intelligence and big data techniques for inverse design of pore structures and performance prediction, enabling quantitative structure–property correlation models. These advances will be crucial for leveraging microporous carbon materials in next-generation energy storage applications.

#### 4.1.2 Mesoporous Carbon

Mesoporous carbons, defined by pore sizes in the range of 2–50 nm, constitute a pivotal class of porous carbon materials for fast-charging energy storage systems.^138^ As schematically summarized in Figure 11, the formation of ordered mesoporous carbons is generally governed by template and assembly directed strategies, in which the assembly of pore-directing agents and carbon precursors determines the resulting pore size, connectivity, and structural order.^135^

**Figure 11:**
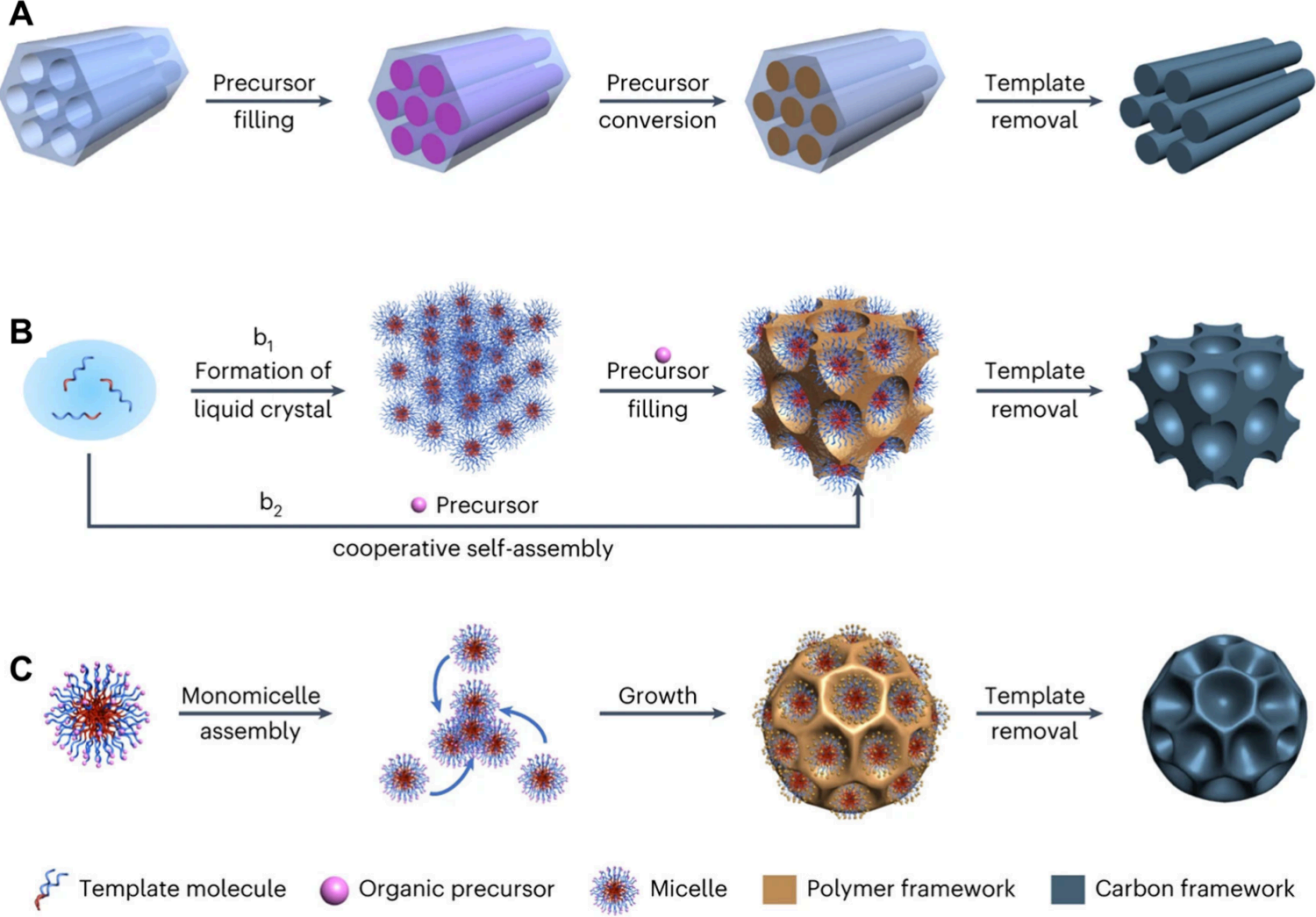
Principal formation mechanisms for the synthesis of mesoporous carbonaceous nanomaterials. A) Hard-templating method. This method implies the use of inorganic materials as a rigid template for constructing ordered mesoporous materials. B) Soft-templating methods use either ‘true’ liquid-crystal or cooperative self-assembly templating processes. The soft-templating method aims for the coassembly of surfactants and/or amphiphilic block copolymers and guest species into ordered mesoporous materials. C) The monomicellar assembly method. This method adopts a single micelle as an assembling unit for constructing ordered mesoporous materials.^135^ Reproduced with permission from ref 135. Copyright 2023 Springer Nature.

From an electrochemical perspective, interconnected mesoporous networks provide continuous ion transport pathways, effectively alleviating diffusion limitations inherent to purely microporous architectures. The enhanced electrolyte wettability and reduced transport resistance afforded by mesoporous channels are particularly beneficial under high charging rates. Moreover, mesoporous carbons offer large specific surface areas and pore volumes, enabling accelerated interfacial charge storage kinetics and allowing the fabrication of thick electrodes without pronounced power loss, thereby bridging the energy–power density tradeoff. However, although mesoporous carbons facilitate ion transport and rate capability, their lower packing density often results in inferior volumetric capacitance compared to denser microporous systems.

In addition to supercapacitors, mesoporous carbons have been widely employed as robust hosts for alloy-type anodes (e.g., Si-based materials) and as confinement matrices for suppressing polysulfide shuttling in Li–S batteries. Notably, hierarchical architectures that integrate micropores within mesopore walls further enhance energy density while preserving rapid ion transport, highlighting the importance of rational pore architecture design for high-rate electrochemical applications.

In the following subsections, we discuss representative synthetic approaches for mesoporous carbons, with emphasis on their structure–property relationships, advantages and limitations, and their relevance to fast-charging energy storage systems.

##### 4.1.2.1 Hard-Template Method

The hard-template method remains one of the most effective approaches for synthesizing mesoporous carbon materials. This strategy typically involves three key steps: template selection, carbon precursor infiltration, and template removal. Among them, the choice of template critically determines the resulting pore architecture and surface morphology. Since the pioneering work by Ryoo and Hyeon in 1999,^221–223^ which demonstrated the use of silica resins as hard templates to fabricate mesoporous carbons, template-directed synthesis has garnered significant research interest. As illustrated in Figure 12A, they coated phenolic resin onto colloidal silica, followed by high-temperature carbonization and template removal with HF, yielding mesoporous carbon with a surface area of up to 1512 m^2^·g^–1^ and a pore size of approximately 12 nm.^221^ Subsequently, they used various silica sources—including silica gel and colloidal silica—with controllable particle sizes to finely tune the mesopore diameters. To achieve more ordered structures, researchers frequently employ ordered mesoporous silica templates such as SBA-15, in combination with polymers or small organic molecules as carbon sources, to prepare structurally regular mesoporous carbons (e.g., CMK-3) that often exhibit a 2D hexagonal symmetry (p6 mm) (Figure 12B).^151^ In recent years, commonly used inorganic hard templates have included a wide range of mesoporous silica families, such as SBA series (SBA-1, SBA-15, SBA-16),^224–227^ MCM series (MCM-41, MCM-48),^227–229^ KIT series (KIT-5, KIT-6),^230,231^ MCF, and FDU series (e.g., FDU-1, FDU-12).^175,232,233^ These templates feature well-defined, tunable pore structures, enabling precise control over pore size, morphology, and specific surface area of the resulting carbon materials.

**Figure 12:**
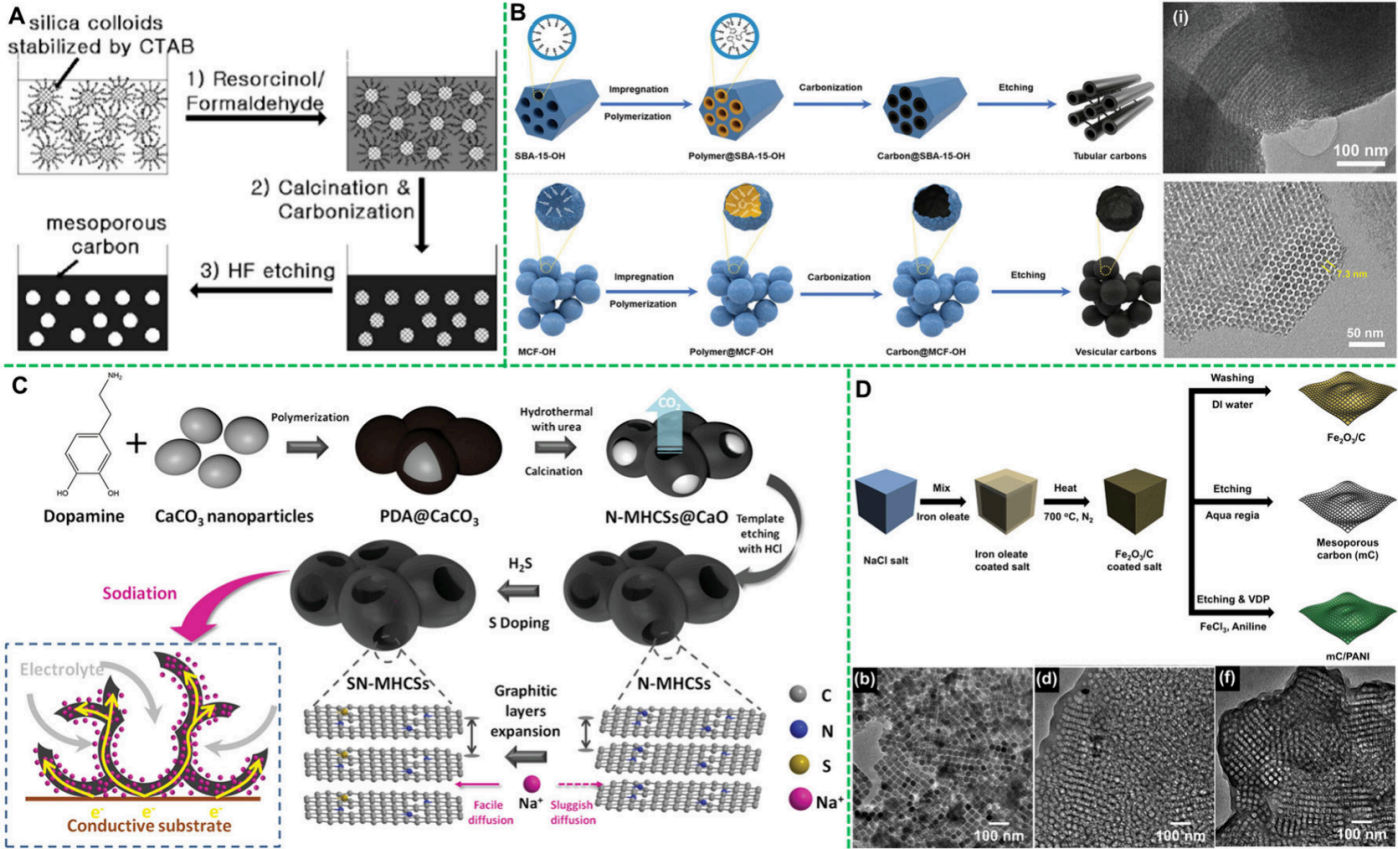
A) Simple silica-particle template synthesis of mesoporous carbons.^221^ Reproduced with permission from ref 221. Copyright 1999 Royal Society of Chemistry. B) Schematic representation of the silanol-assisted surface-casting strategy with different mesoporous silica templates.^151^ Reproduced with permission from ref 151. Copyright 2025 Wiley. C) synthesis of mesoporous hollow carbon spheres using dopamine as a carbon source, and nano-CaCO_3_ as a template.^150^ Reproduced with permission from ref 150. Copyright 2019 Wiley. D) Schematic of polyaniline-coated mesoporous carbon nanosheets: Fe_2_O_3_/C precursors are pyrolyzed in a NaCl template, washed and acid-etched to form mesoporous carbon, then coated with polyaniline via vapor-phase polymerization.^137^ Reproduced with permission from ref 137. Copyright 2023 Wiley.

Although this “nanocasting” technique offers excellent structural control, the use of HF for template removal raises significant concerns regarding corrosion and environmental impact. To address this, alternative template systems based on metal oxides (e.g., CaCO_3_, Fe_2_O_3_, MgO, CoP)^137,150,234,235^ or even inorganic salts have been developed, enabling template removal under milder acidic conditions. For instance, Dong and co-workers employed a self-assembled Fe_3_O_4_ superlattice as a sacrificial template to construct a mesoporous graphene framework (MGF) with face-centered cubic symmetry and interconnected mesoporous architecture.^234^ The resulting material exhibited high specific surface area, excellent structural integrity, and electrical conductivity, delivering promising rate capability and cycling stability as a lithium-ion battery anode. Using templates such as CaCO_3_ (Figure 12C) or MgO in combination with carbon sources like dopamine or glucosamine, researchers have synthesized mesoporous carbon with average pore sizes around 8.8 nm and nitrogen-doped porous carbons featuring both mesopores and hierarchical porosity.^150,235^ It was also found that varying the carbon precursor can lead to in situ heteroatom doping, thereby enhancing electrochemical performance. Inorganic salts such as sodium chloride (NaCl) can also serve as sacrificial templates. Yoon et al. thermally decomposed an iron–oleate complex within a NaCl matrix to obtain carbon frameworks, and subsequently removed embedded cubic Fe_2_O_3_ nanocrystals to form mesoporous carbon (Figure 12D). They further improved conductivity by in situ deposition of polyaniline via vapor polymerization. The resulting materials exhibited excellent supercapacitor performance, with a capacitance of 107.8 F·g^–1^ at 0.5 A·g^–1^, along with high energy density (9.59 Wh·kg^–1^) and power density (200.1 W·kg^–1^), as well as fast charge transfer behavior.^137^

Overall, the hard-template method offers remarkable advantages in structural control, enabling the synthesis of highly ordered mesoporous carbons with precisely tunable pore sizes, geometries, and interconnectivities. These materials exhibit high surface areas and hierarchical architectures that maintain rapid ion transport, even under high mass loadings. However, the hard-templating route often involves multiple complex steps, large quantities of template material, and environmental concerns related to template removal, thereby presenting challenges for large-scale and sustainable production. Consequently, the development of greener, more scalable, and simplified synthesis strategies remains a critical priority for future research.

##### 4.1.2.2 Soft-Templating Method

The soft-templating method primarily relies on self-assembly between carbon precursors and templates to form ordered organic–organic composites. The evolution pathways of such systems can be generally categorized into two representative strategies: (1) Prepolymer-assisted self-assembly: In this approach, precondensed phenolic resins are mixed with block copolymers in a solvent to form ordered composite phases via evaporation-induced self-assembly (EISA), followed by carbonization to yield mesoporous carbon films. A typical example involves the coassembly of phenolic resins and Pluronic F127 under acidic conditions, producing hexagonal or cubic mesostructures. EISA serves as a key step to guide the formation of ordered architectures, where the evaporation rate of the solvent is finely tuned to facilitate cooperative assembly between the carbon source and template. This strategy offers advantages such as good structural stability and controllable synthesis. (2) In-situ polymerization with microphase separation: In this strategy, carbon-source monomers (e.g., phenol and formaldehyde) undergo polymerization in the presence of a block copolymer template. The polymerization and phase separation processes proceed simultaneously, enabling the in situ formation of mesoporous structures. This typically involves direct polymerization under high-temperature or strongly acidic/alkaline conditions. The formation of ordered mesostructures can be further enhanced by incorporating the EISA mechanism. This approach is particularly favorable for enlarging pore sizes and fine-tuning the porosity.

Among these methods, EISA has emerged as a crucial mechanism for fabricating ordered mesoporous carbon materials. In 2004, Dai and co-workers pioneered the synthesis of ordered mesoporous carbon films with hexagonal structures by employing a noncommercial diblock copolymer, polystyrene-block-poly­(4-vinylpyridine) (PS-b-P4VP), as the soft template and resorcinol–formaldehyde as the carbon source (Figure 13A). Preassembly into mesostructures was achieved through spin coating and solvent annealing, followed by in situ polymerization under formaldehyde vapor and subsequent carbonization in nitrogen, yielding mesoporous carbon with a pore size of ∼34 nm. This work sparked significant interest in soft-templated mesoporous carbon synthesis.^236^ Subsequently, Tanaka and colleagues developed a similar synthetic route to prepare COU-1 mesoporous carbon films with pore sizes of 10–15 nm by replacing the noncommercial PS-b-P4VP with the commercially available triblock copolymer Pluronic F127 (EO_106_PO_70_EO_106_).^239^ Ethyl orthocarboxylate (EOA) was later introduced as an auxiliary carbon source to enhance the dispersion of the carbon precursor and the integrity of the pore architecture. Nevertheless, challenges such as pore collapse and structural shrinkage during carbonization still posed limitations to stability.^240^

**Figure 13:**
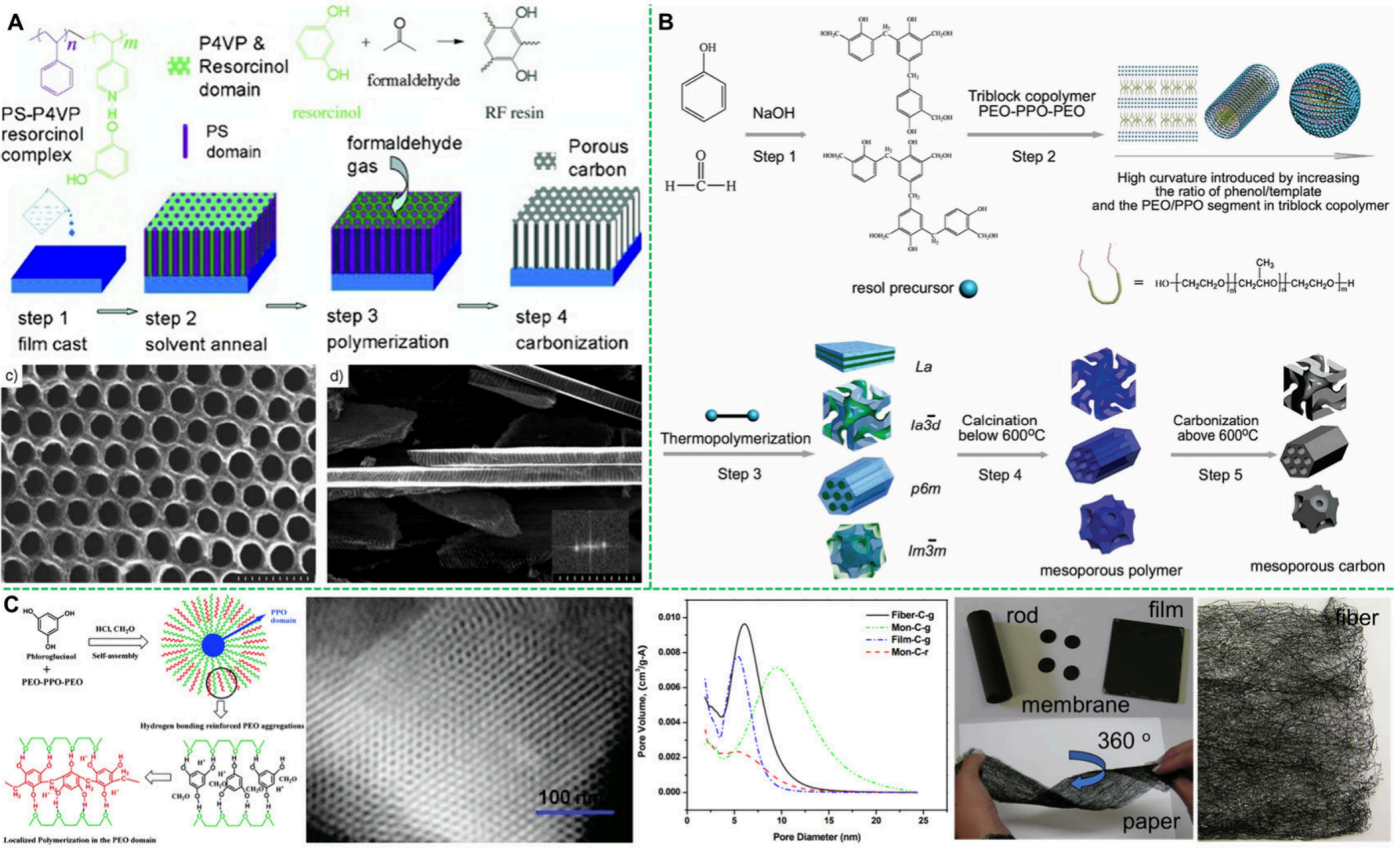
A) Schematic representation of a stepwise self-assembly approach for preparing large-scale, highly ordered nanoporous carbon films.^236^ Reproduced with permission from ref 236. Copyright 2004 Wiley. B) Scheme for the preparations of the ordered mesoporous polymer resins and carbon frameworks.^237^ Reproduced with permission from ref 237. Copyright 2006 American Chemical Society. C) Synthesis of mesoporous carbons by two-phase self-assembly.^238^ Reproduced with permission from ref 238. Copyright 2006 American Chemical Society.

To improve the versatility and processability of soft-template strategies, Zhao and Dai’s groups carried out extensive studies using commercially available, low-cost triblock copolymers (e.g., F127, P123, F108) and phenolic resins as carbon sources.^135,163,227^ Their efforts significantly advanced the practical application of the EISA approach in mesoporous carbon synthesis. In their strategy, phenolic resins and Pluronic copolymers are codissolved in ethanol under basic conditions (Figure 13B).^237^ Hydrogen bonding between the hydroxyl groups of phenolic resins and the PEO segments of the block copolymers drives organic–organic self-assembly during solvent evaporation, forming ordered mesostructures. Under acidic conditions, phloroglucinol–formaldehyde, resorcinol–formaldehyde, or phloroglucinol–glyoxal resins are polymerized in the presence of Pluronic copolymers (Figure 13C).^238^ The hydroxyl-rich, partially negatively charged phenolic intermediates interact strongly with the protonated PEO segments through electrostatic attraction and hydrogen bonding, resulting in stable and highly ordered organic–organic composites. These intermediates can be directly cast or spin-coated on various substrates from THF–ethanol mixtures, followed by in situ polymerization, microphase separation, and EISA to construct ordered mesoporous structures. Upon drying, the polymeric templates are removed by thermal decomposition or acid extraction, yielding ordered mesoporous carbon frameworks. Studies show that the pore structure can be tailored by adjusting the copolymer/resin ratio, the type of copolymer, and the solution pH.^227,238,245^ Typically, prepolymer-based strategies yield mesoporous carbons with pore sizes in the range of 2–5 nm, whereas in situ polymerization with microphase separation enables the formation of larger pores (>6 nm). The two strategies are complementary in terms of pore size control, thereby expanding the structural options of ordered mesoporous carbons for applications in energy storage, adsorption, and beyond.

The soft-templating method not only enables precise control over the pore size and ordering of mesoporous carbons but also allows for the rational design of diverse particle morphologies and hierarchical pore structures by tuning the assembly conditions. Traditionally, this method has been employed to synthesize symmetric spherical particles with uniform mesoporous architectures. However, recent advances have significantly expanded the structural complexity achievable via soft-templating. Lou and co-workers introduced an emulsion-directed interfacial anisotropic assembly strategy to construct bowl-shaped mesoporous carbon particles with radially oriented open channels (Figure 14A).^241^ By modulating the interfacial interactions between F127 micelles swollen with 1,3,5-trimethylbenzene (TMB) and dopamine precursors within emulsion droplets, they achieved unprecedented asymmetric morphologies that retained open pore structures after carbonization. This study represents a conceptual breakthrough, demonstrating for the first time that soft-templating can overcome the intrinsic symmetry of micelle packing to achieve anisotropic mesostructures. Building upon this, they further developed a dual soft-templating strategy employing mixed Pluronic P123/F127 systems with TMB to fabricate walnut-shaped carbon particles with interconnected large mesopores.^246^ By precisely tuning the template ratios and ethanol concentrations, they successfully induced a transition from hexagonally packed cylindrical pores to bicontinuous mesophases, enabling adjustable mass transport pathways and enhanced accessibility—attributes particularly advantageous for electrocatalytic applications such as oxygen reduction reactions (ORR). Peng and Zhao et al. demonstrated that simply tuning the stirring rate allows control over micelle size, and proposed a programmable shear-induced dynamic assembly strategy for synthesizing mesoporous carbon nanospheres with radially graded channels and core–shell structures (Figure 14B).^242^ The resulting materials exhibited outstanding sodium-ion storage performance, including high capacity and ultralong cycling stability. Through compositional tuning to modulate micelle curvature and spatial organization, Peng et al. further advanced the field by developing helical self-assembly of layered micelles under shear flow, yielding multilayered hollow carbon nanospheres with chiral architectures (Figure 14C).^243^ These structures exhibit a combination of high surface area, nitrogen doping, and uniform mesopores (∼2.5 nm), embodying both aesthetic appeal and functional performance. Notably, they demonstrated outstanding potassium-ion storage capacity and proposed a generalizable design principle based on “hydrophilic/hydrophobic ratio-controlled micelle curvature.

**Figure 14:**
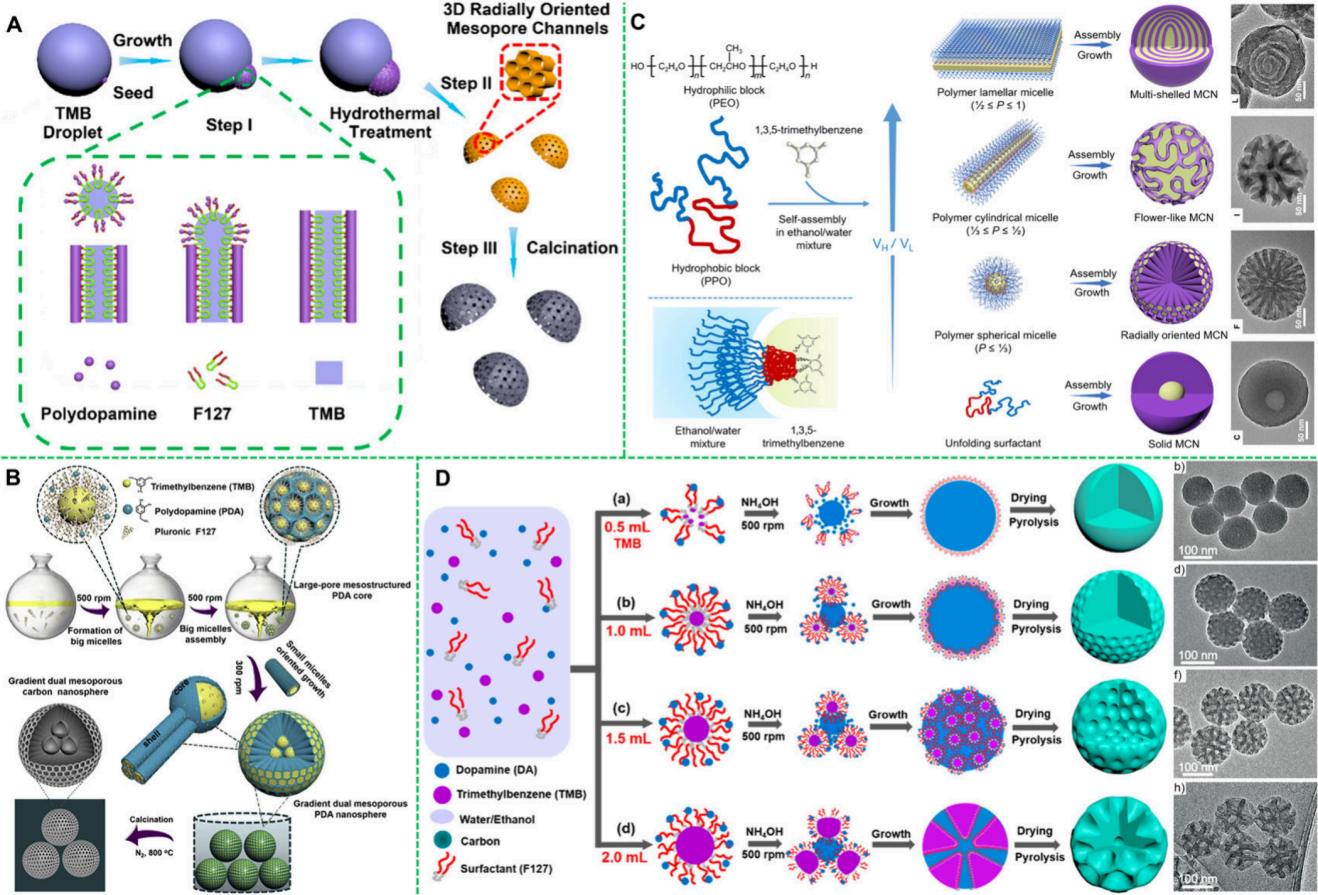
A) Schematic illustration of the formation process of asymmetric bowl-like mesoporous particles via emulsion-induced interface anisotropic assembly.^241^ Reproduced with permission from ref 241. Copyright 2016 American Chemical Society. B) Schematic of the programmable synthesis of radially gradient-structured mesoporous carbon nanospheres.^242^ Reproduced with permission from ref 242. Copyright 2021 Elsevier. C) Schematic illustration of the spiral self-assembly of lamellar micelles into multishelled hollow nanospheres with unique chiral architecture.^243^ Reproduced with permission from ref 243. Copyright 2021 American Association for the Advancement of Science. D) Schematic illustration of the formation process for the N-doped mesoporous carbon nanospheres with various morphologies and mesostructures prepared by the versatile nanoemulsion assembly approach.^244^ Reproduced with permission from ref 244. Copyright 2019 American Chemical Society.

Expanding on these insights, the same group introduced a TMB-mediated interfacial interaction mechanism to synthesize mesoporous nitrogen-doped carbon spheres with various complex morphologies—golf ball-like, multicavity, and dendritic structures (Figure 14D).^244^ The pore sizes were precisely tuned between 5 and 37 nm, significantly enhancing their ORR catalytic performance. In summary, these studies mark a significant advancement in soft-templating chemistry—from static micelle-directed assembly to dynamic, interface-guided morphogenesis. By integrating block copolymers, emulsifiers, swelling agents (e.g., TMB), and biomass-derived precursors into cooperative templating systems, researchers have established generalizable strategies for constructing asymmetric, hierarchical, and multimodal mesoporous carbon materials.^13,247,248^ These innovations not only offer aesthetic and structural benefits but also deliver practical advantages in energy storage and electrocatalysis, as open and oriented pore systems facilitate ion diffusion and accessibility to active sites.

##### 4.1.2.3 Dual-Templating Method

While hard- and soft-templating methods have emerged as the two primary approaches for constructing ordered mesoporous carbons, each exhibits inherent limitations. Hard-templating allows for precise control over pore architecture and size; however, it often suffers from limited template diversity, labor-intensive procedures, and risks of structural collapse or contamination during template removal. In contrast, soft-templating offers advantages such as ease of processing and compatibility with large-area film deposition, but it frequently faces challenges in scaling up to macroscopic dimensions, increasing film thickness, and maintaining structural integrity. To address these issues, researchers have increasingly explored dual-templating strategies, which integrate the benefits of both approaches to achieve materials with microstructural order and macroscale structural tunability. This method typically introduces a second functional template or structural support—such as 3D foam scaffolds or 2D layered materials—thereby preserving control over mesopore formation while simultaneously imparting additional functionalities and structural advantages.

A representative limitation of conventional soft templating is its reliance on the self-assembly of triblock copolymers and carbon precursors on planar substrates. When attempting to extend the film thickness beyond several hundred micrometers, issues such as structural disorder and pore blockage often arise, severely limiting large-scale applicability. To overcome this, Xue and co-workers proposed a modified EISA strategy using a polyurethane (PU) foam scaffold instead of planar substrates (Figure 15A). The hydrophilic PU foam, with its 3D interconnected macroporous framework, significantly increases the number of assembly interfaces and the available spatial domains for structure formation. In this system, ethanol solutions containing the carbon precursor (e.g., resol-type phenolic resin) and F127 infiltrate the foam network via a capillary action. During subsequent evaporation-induced self-assembly, ordered mesostructures formed within the 3D matrix. Upon carbonization, both the PU scaffold and the soft template are effectively removed, resulting in a 3D macroporous/mesoporous carbon framework with a hierarchical porosity.^249^ This method successfully overcomes the spatial limitations of traditional EISA and offers a scalable pathway to producing ordered mesoporous carbons.

**Figure 15:**
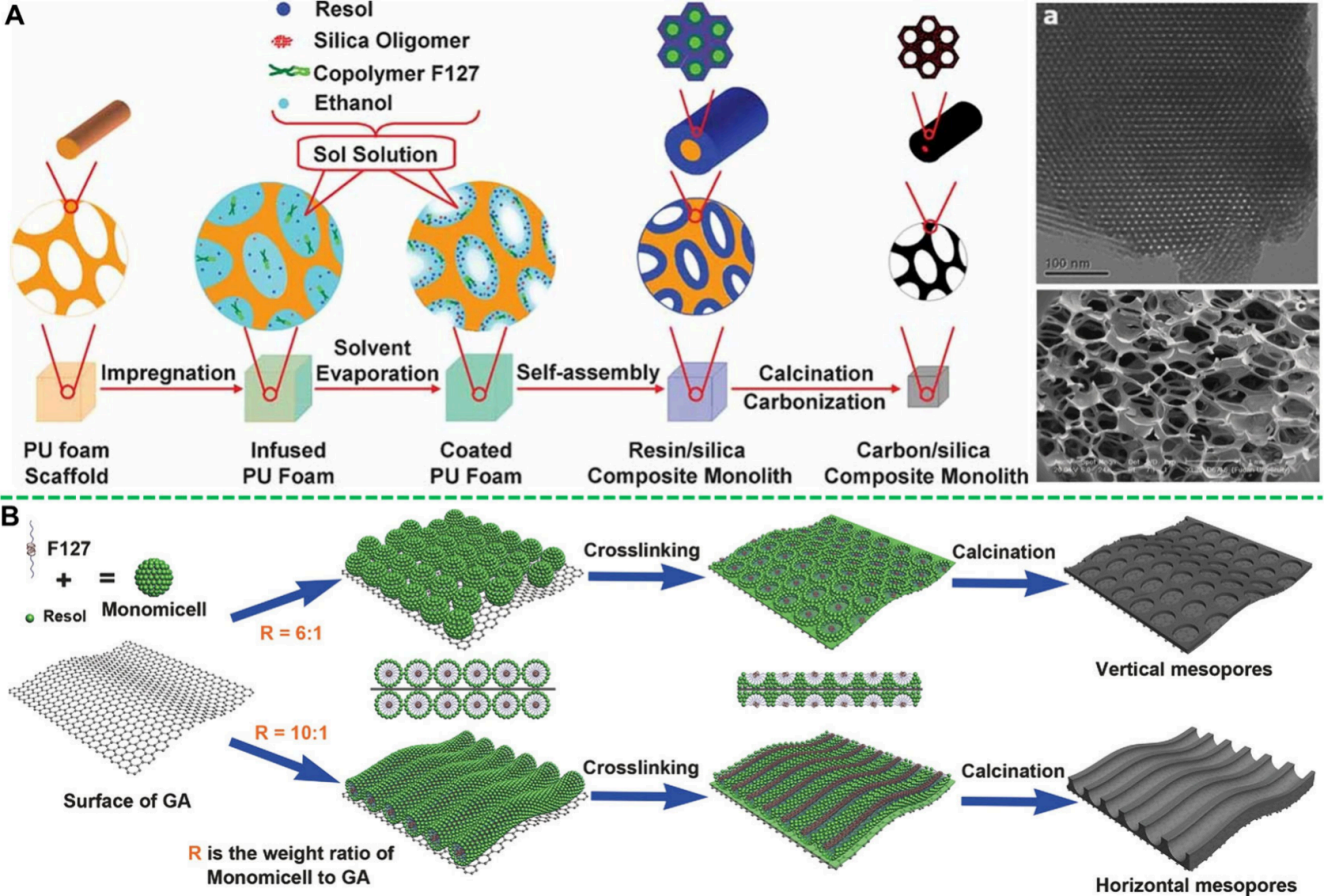
A) The scheme for the formation of hierarchical porous composite monoliths templated by PU foamsvia a solvent-evaporation-induced coating and self-assembly method.^249^ Reproduced with permission from ref 249. Copyright 2008 Wiley. B) Schematic illustration of the formation process for ordered mesoporous carbon (OMC) on Graphene aerogel (GA).^250^ Reproduced with permission from ref 250. Copyright 2015 Wiley.

Alternatively, assembling ordered mesoporous carbons onto functional two-dimensional substrates represents another effective dual-templating approach. For example, Liu and colleagues developed an interfacial assembly method in which F127-induced micelles coassemble on the surface of a graphene aerogel, forming a mesoporous carbon layer with an average pore size of ∼9 nm (Figure 15B). By adjusting the micelle-to-graphene ratio, a morphological evolution from spherical to cylindrical pores was achieved, demonstrating fine-tuned control over the microstructure.^250^ In addition to graphene, other 2D materials such as graphene oxide, MXenes, metal oxides, layered double hydroxides (LDHs), and carbon nanotubes (CNTs) have been widely incorporated to further functionalize mesoporous carbon composites.^251–253^ In a notable example, Zhang et al. utilized a phytate-assisted molecular engineering strategy to assemble N, P-codoped mesoporous carbon onto Ti_3_C_2_T_x_ MXene nanosheets. Under the cooperative action of P123 and melamine-formaldehyde (MF) resin, this approach significantly enhanced hydrogen bonding interactions and enabled precise control of the interfacial self-assembly process.^254^ In summary, dual-templating strategies, which combine structural guiding agents with functional scaffolding substrates, enable the synergistic formation of well-ordered pore architectures, flexible assembly behavior, and controlled macroscopic morphologies. This approach provides a versatile platform for the development of next-generation high-performance mesoporous carbon materials, offering tunable structural features and enhanced functionalities for energy storage, catalysis, and beyond.

##### 4.1.2.4 Monomicellar Assembly Strategy

Distinct from conventional hard- and soft-templating approaches, the monomicellar assembly strategy represents an emerging and highly controllable route for the synthesis of ordered mesoporous carbons, in which a single micelle acts as the fundamental structure-directing unit for one mesopore. In this approach, amphiphilic block copolymers or surfactants coassemble with carbon precursors to form discrete and stable monomicelles through hydrogen bonding, electrostatic interactions, and hydrophobic effects. Each monomicelle functions as an independent “building block”, and their subsequent organization dictates the mesoscopic architecture of the resulting carbon framework

A key advantage of the monomicellar assembly method lies in its decoupling of micelle formation and mesoscale assembly, enabling precise control over pore size, shape, and spatial arrangement. Unlike classical soft-templating routes that rely on collective micellar mesophases, monomicelles can be individually stabilized and manipulated prior to assembly, thereby suppressing uncontrolled aggregation and phase separation that often lead to disordered worm-like mesostructures. By tuning parameters such as block copolymer composition, swelling agents, solvent polarity, and stirring conditions, monomicellar dimensions can be continuously adjusted, allowing the pore sizes of mesoporous carbons to be tuned over a wide range (typically ∼5–40 nm) with narrow distributions

The monomicellar assembly strategy also offers exceptional structural versatility, enabling the construction of mesoporous carbons with diverse morphologies, including radially oriented mesoporous nanospheres, multishelled hollow structures, flower-like architectures, and hierarchical multilevel pore systems. For example, a low-concentration monomicellar close-packing strategy has enabled the fabrication of two-dimensional mesoporous carbon (or graphene) nanosheets with vertically aligned mesopores, forming an orthogonal architecture that facilitates rapid ion transport and strain accommodation (Figure 16).^255^ Moreover, anisotropic assembly or interfacial confinement of monomicelles permits the fabrication of complex heterostructures such as core–shell, Janus, and sandwich-like mesoporous carbons, which are difficult to achieve via traditional templating methods. These features make monomicellar assembly particularly attractive for designing mesoporous carbons with tailored ion transport pathways and spatially programmed functionality

**Figure 16:**
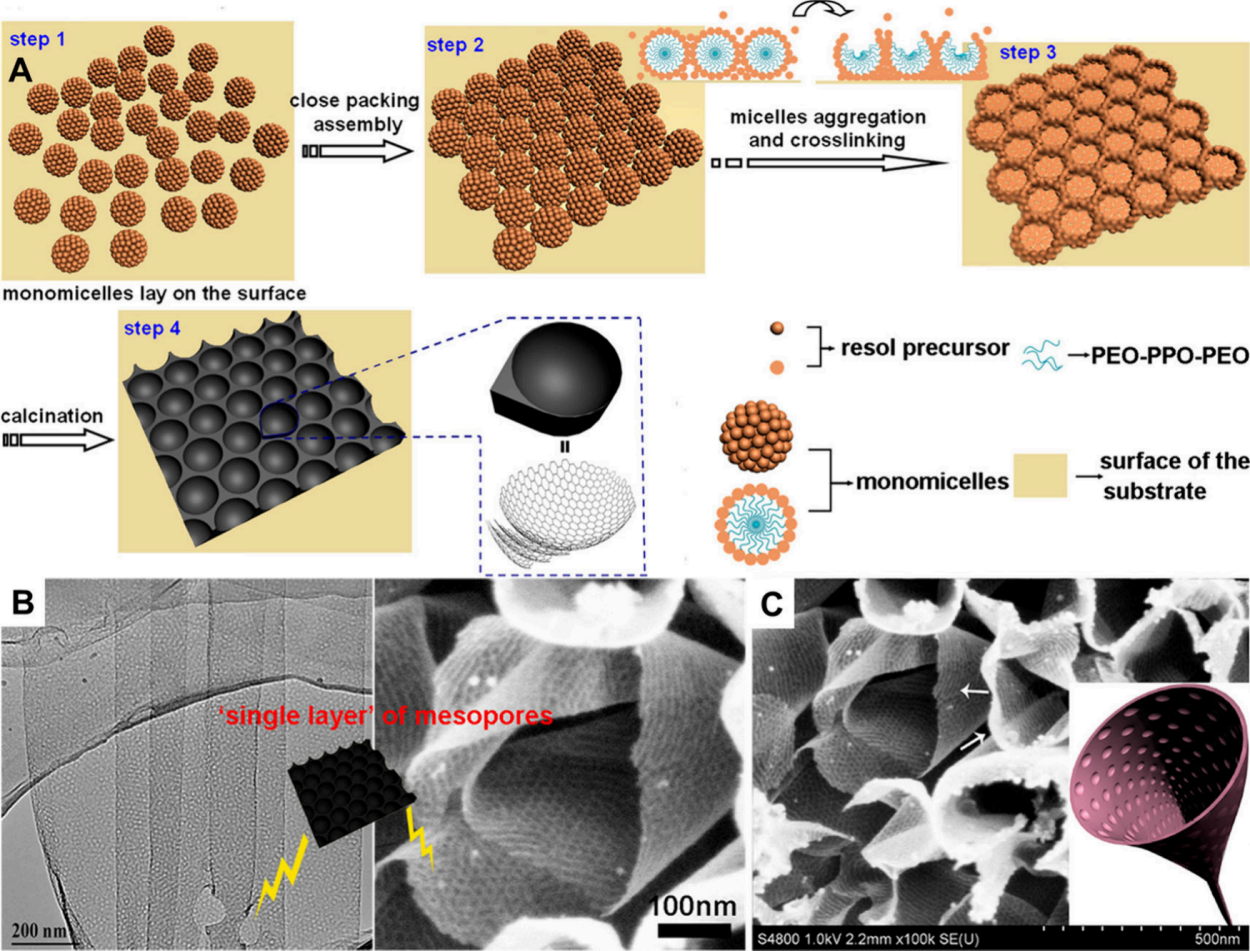
A) Schematic illustration for synthesizing 2D mesoporous carbon nanosheets via the interfacial assembly of F127/resol monomicelles. B) TEM and C) SEM images of the mesoporous carbon nanosheets.^255^ Reproduced with permission from ref 255. Copyright 2013 American Chemical Society.

From an electrochemical perspective, mesoporous carbons derived from monomicellar assemblies exhibit several attributes favorable for fast-charging energy storage. The uniformly distributed and interconnected mesopores facilitate rapid electrolyte infiltration and reduce ion diffusion resistance, while the nanoscale carbon frameworks shorten the electron transport pathways. In addition, the use of nitrogen-rich precursors such as dopamine enables in situ heteroatom doping, further enhancing the surface wettability, electronic conductivity, and pseudocapacitive contributions. As a result, monomicellar-derived mesoporous carbons have demonstrated promising performance in high-rate supercapacitors, sodium-ion batteries, and electrocatalytic reactions, where fast mass and charge transport are critical.

Despite these advantages, several challenges remain for the practical implementation of the monomicellar assembly strategy. The requirement for low micelle concentrations to maintain monodispersity limits production efficiency, and the sensitivity of monomicelle stability to stirring, temperature, and solvent composition pose challenges for large-scale synthesis. In addition, extending this method to incorporate highly reactive inorganic precursors or metallic species remains to be nontrivial. Nevertheless, continued advances in molecular-level assembly control and process optimization are expected to further expand the applicability of monomicellar assembly for rationally designed mesoporous carbons in fast-charging energy-storage systems.

##### 4.1.2.5 Mesopore-Enabled Selective Ion Charging for Energy–Power Synergy

Subnanometer micropores are widely acknowledged as the primary charge-storage domains in electric double-layer capacitors (EDLCs), owing to strong ion confinement, suppressed overscreening, and enhanced surface-specific capacitance.^164,169^ Theoretical models developed by Kondrat and Kornyshev predicted that, under extreme confinement, ionic liquids can enter a “superionic” state, where counterions pack densely and contribute disproportionately to energy density.^71,113^ However, the same confinement that benefits energy storage inevitably leads to sluggish ion transport, particularly under high current densities, resulting in pronounced power limitations.

Experimental studies by Simon and co-workers subsequently demonstrated that the charging mechanism in porous carbons is highly pore-size dependent.^68,77,83^ In micropores, charge compensation proceeds predominantly through ion exchange or coion desorption, processes that are energetically favorable but kinetically slow due to high in-pore ion density and strong ion–ion correlations. In contrast, mesopores exhibit charging behavior closer to counterion adsorption, accompanied by lower resistance and faster kinetics. These findings established that mesopores are essential for sustaining high-rate performance, even though their intrinsic capacitance per surface area is typically lower than that of micropores.

Mesoporous carbons (2–50 nm) therefore play a fundamentally distinct role from microporous structures by primarily facilitating rapid ion transport rather than maximizing charge storage. Their larger pore sizes significantly reduce diffusion resistance, enabling efficient electrolyte penetration and excellent rate capability, particularly under high charging conditions. However, this kinetic advantage comes at the expense of reduced surface area and weaker ion confinement, resulting in lower intrinsic capacitance compared to microporous carbons. Consequently, purely mesoporous architectures are generally insufficient for achieving high energy density. A key insight emerging from comparative studies is that mesopores function predominantly as ion-buffering reservoirs and transport highways, rather than active charge storage sites. Their primary role is to enhance the accessibility and utilization of adjacent microporous domains, where charge storage mainly occurs. Therefore, mesoporosity should not be regarded as an independent design strategy, but rather as a complementary structural feature. The synergistic integration of mesopores and micropores is essential for overcoming the energy–power tradeoff, forming the basis of high-performance hierarchical porous carbons.

Molecular simulations by Merlet, Salanne, and co-workers provided microscopic insight into this dichotomy.^87,258^ Their MD and cDFT studies revealed that ions confined in micropores experience strong electrostatic correlations and reduced mobility, whereas mesopores maintain ion distributions and diffusion coefficients closer to bulklike behavior. Importantly, mesopores were shown to function as ion reservoirs or buffers, dynamically supplying counterions to adjacent microporous domains during charging while mitigating the concentration polarization. These results imply that mesopores actively regulate charging dynamics rather than serve merely as passive diffusion channels.

Building on these conceptual advances, Wang et al. provided direct experimental evidence for mesopore-enabled selective ion charging using an ionic mixture electrolyte in carbons with tailored pore architectures.^166^ By combining electrochemical measurements, solid-state NMR, and molecular simulations, they demonstrated that cations with weaker ion–ion interactions selectively enter micropores, whereas larger or more strongly interacting cations are excluded from micropores but readily accommodated within mesopores (Figure 17A). This selective partitioning enables dense ion packing in mesopores while preserving rapid ion transport in micropores, thereby decoupling charge accommodation from ion diffusion. Crucially, such selective charging behavior allows mesopores to serve as dynamic ion-buffering regions under nonequilibrium conditions. During fast charging, ions first accumulate in mesopores with minimal desolvation or steric penalties, establishing local charge compensation and suppressing diffusion overpotentials. A subset of counterions is then selectively transferred into energetically favorable micropores, where high charge density is stabilized. Simultaneously, mesopores suppress inefficient coion participation and excessive ion exchange, reducing entropy losses and voltage hysteresis at high rates.

**Figure 17:**
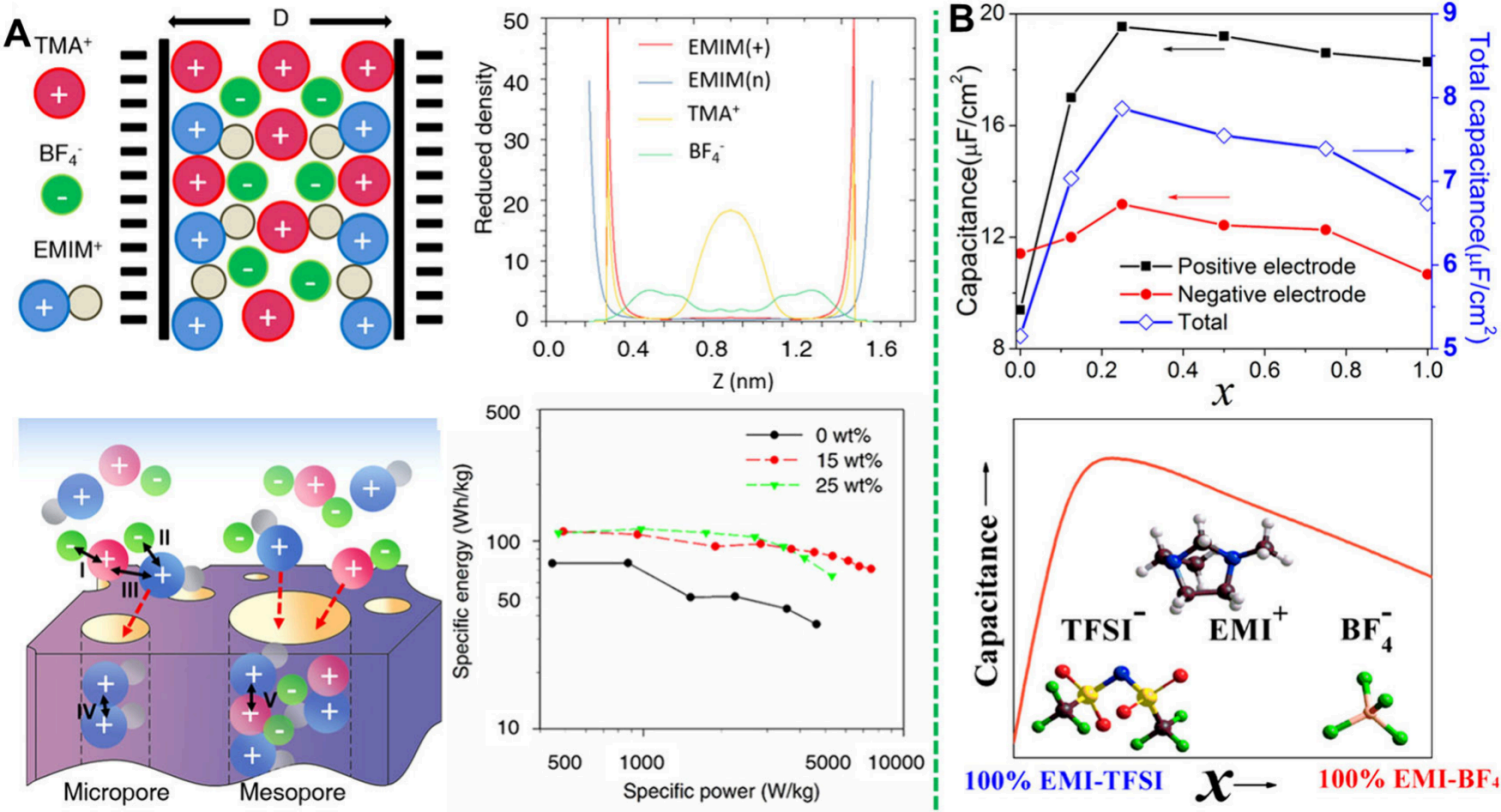
A) Schematic and classical density functional theory (cDFT)-derived ion distributions illustrating interaction-induced selective charging in an ionic mixture electrolyte confined within a charged slit pore. TMA^+^, BF_4_^–^, and EMIM^+^ ions are represented using coarse-grained models based on molecular volume. Black arrows denote dominant ionic interactions (I–V), while red arrows indicate ion diffusion pathways, highlighting preferential counterion packing and selective ion transport in confined geometries.^166^ Reproduced with permission from ref 166. Copyright 2017 American Chemical Society. B) Number density profiles of counterions adjacent to positively and negatively charged electrodes for EMI-TFSI, EMI-BF_4_, and their mixtures, revealing a volcano-shaped dependence of capacitance on electrolyte composition arising from enhanced counterion accumulation and coion depletion at the electrode–electrolyte interface.^256^ Reproduced with permission from ref 256. Copyright 2016 American Chemical Society.

It should be noted that the use of ionic mixtures to enhance capacitive performance is not restricted to pore-confinement-driven selectivity. In a complementary strategy demonstrated by Lian, Wu, Gogotsi, and co-workers using nonporous onion-like carbon electrodes, capacitance enhancement was achieved through electrolyte-induced restructuring of the electric double layer itself. In this case, a binary ionic liquid mixture sharing a common cation but comprising anions of different sizes enabled preferential adsorption of the smaller anion at the electrode surface (Figure 17B), effectively reducing the EDL thickness, suppressing overscreening, and maximizing interfacial charge density—even in the absence of microporous size sieving.^256^ Such electrolyte-driven interfacial regulation provides a conceptual basis for more complex multicomponent systems, including emerging high-entropy electrolytes,^259–262^ where diverse ionic species can further tailor EDL structure and charge distribution.

From an energy–power perspective, this hierarchical charging process offers a clear pathway to synergy. Micropores maximize energy density by stabilizing densely packed counterions, as predicted by theory and confirmed experimentally, while mesopores sustain power delivery by buffering ion transport and regulating selective ion access. Together with interfacial ion-packing optimization enabled by electrolyte composition, these studies highlight ionic mixture design—particularly its extension to multicomponent systems such as high-entropy electrolytes—as a powerful and versatile lever for decoupling energy storage from ion-transport limitations.^263–273^ The cooperative interplay between micro- and mesopores thus overcomes the classical tradeoff between energy density and power density that plagues purely microporous electrodes.

Mesopores therefore emerge as active regulators of selective ion charging rather than inert transport voids. Rational tuning of mesopore size, connectivity, and volume fraction—particularly in concert with optimized micropore distributions—provides an effective means to decouple charge storage from ion-transport limitations under fast-charging conditions. When combined with electrolyte design strategies that exploit ionic size asymmetry and composition-induced EDL restructuring, this pore-level regulation enables simultaneous optimization of energy density and power capability. This synergistic framework is particularly compatible with high-entropy electrolytes, where compositional complexity enables simultaneous regulation of interfacial ion packing, desolvation processes, transport kinetics, and selective ion access to hierarchical pore networks, thereby offering a powerful route to overcome the traditional energy–power tradeoff in carbon based supercapacitors.

#### 4.1.3 Macroporous Carbon

Macroporous carbons, typically defined by pore sizes exceeding 50 nm, have received comparatively less attention in supercapacitor research because of their intrinsically low surface area and limited direct contribution to charge storage. Nevertheless, growing evidence suggests that macroporosity plays a critical enabling role in facilitating electrolyte transport and maintaining ion accessibility under practical operating conditions, particularly in thick electrodes and at high-rate applications. In contrast to micropores, which dominate electric double-layer formation, and mesopores, which govern ion diffusion, macropores primarily function as ion-buffering reservoirs and long-range transport pathways that reduce the tortuosity and mitigate electrolyte depletion. As such, their importance is most effectively realized within hierarchical pore architectures rather than as isolated structural features.

##### 4.1.3.1 Synthetic Strategies

The construction of macroporous carbons generally follows two primary strategies: template replication and precursor-directed spontaneous pore formation. The former offers superior control over pore size, ordering, and connectivity, enabling the formation of well-defined and periodic architectures, whereas the latter is more advantageous for process simplification and scalable production. Among these approaches, hard-templating represents one of the most established and widely adopted routes for constructing macroporous carbons. Early pioneering work by A. A. Zakhidov demonstrated the replication of colloidal crystal templates (e.g., SiO_2_ opals) into three-dimensionally ordered macroporous (3DOM) carbons (Figure 18A), establishing a general route for inverse opal structures with tunable pore diameters (typically 100 nm–1 μm).^139^ Subsequent studies expanded this approach using monodisperse silica or polymer spheres as sacrificial templates, where carbon precursors infiltrate interstitial voids followed by polymerization/carbonization and template removal.^140–142^ This strategy enables highly ordered macroporous frameworks with interconnected transport pathways, which are particularly advantageous for mass transport in thick electrodes. A representative example is the use of polystyrene colloidal spheres as sacrificial templates to construct three-dimensional macroporous chemically modified graphene (e-CMG) films, which were subsequently decorated with MnO_2_ to form composite electrodes (Figure 18B).^102^ In this system, the interconnected macroporous graphene scaffold provided rapid ion transport pathways while preserving high electronic conductivity, and the conformal MnO_2_ coating further increased charge-storage capacity. As a result, the MnO_2_/e-CMG electrode delivered a high specific capacitance together with exceptional rate capability, and the assembled asymmetric supercapacitor achieved both high energy density and high power density, highlighting the value of ordered macroporous frameworks for simultaneously improving energy–power characteristics. However, despite its structural precision, hard templating remains limited by multistep processing, template removal (often involving hazardous etchants such as HF), and scalability constraints.^274,275^

**Figure 18:**
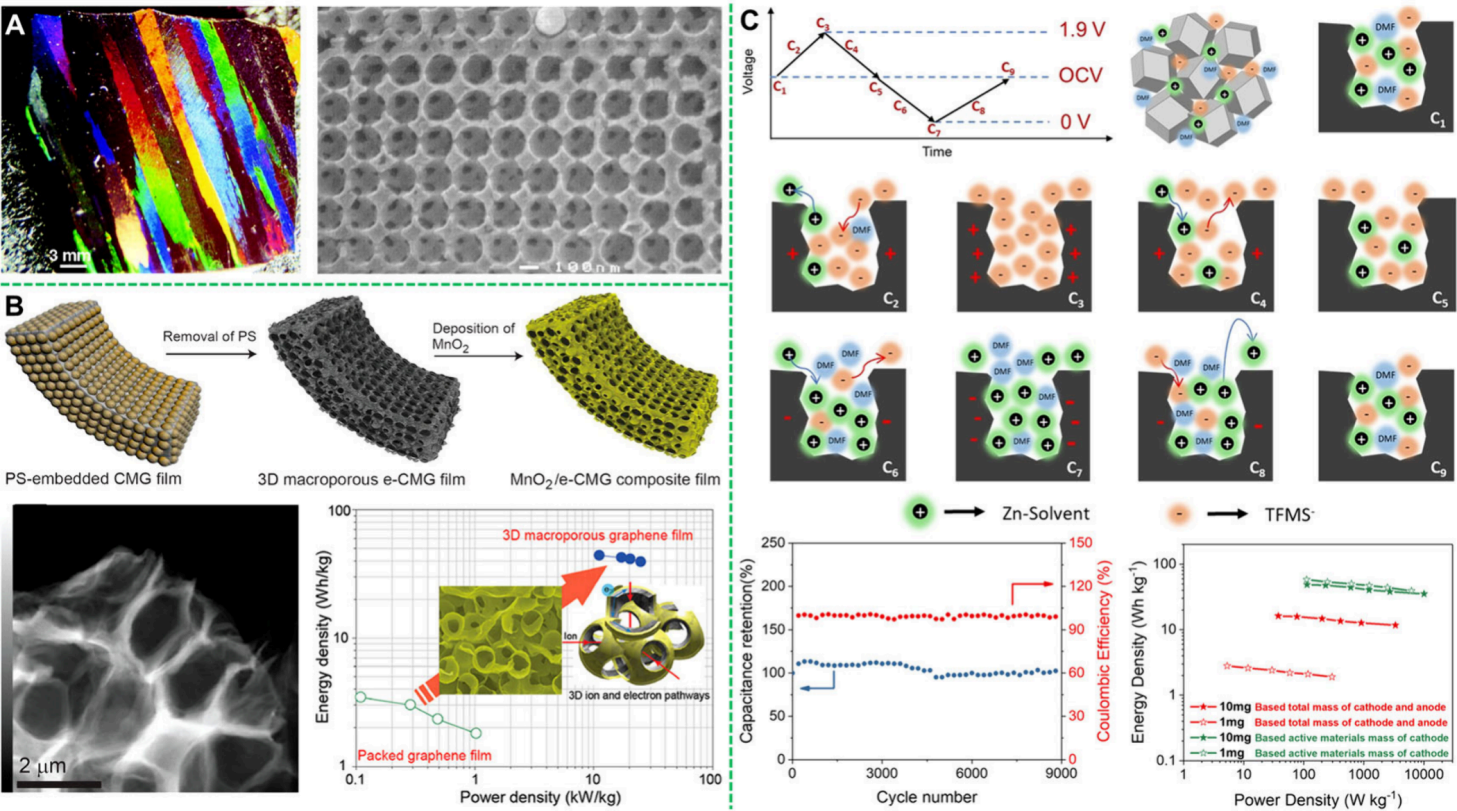
A) Graphitic carbon inverse opal derived from SiO_2_ opal templating: photograph and SEM image of the ordered macroporous framework.^139^ Reproduced with permission from ref 139. Copyright 1998 American Association for the Advancement of Science. B) Schematic illustration of PS-templated fabrication and MnO_2_ integration; HAADF-STEM image showing conformal coating within the interconnected macroporous graphene framework, enabling efficient ion transport.^102^ Reproduced with permission from ref 102. Copyright 2012 American Chemical Society. C) Macropore-based charge storage and electrochemical performance at high mass loading: ion adsorption/desorption schematic; cycling stability and Ragone plot.^143^ Reproduced with permission from ref 143. Copyright 2021 American Chemical Society.

To overcome these limitations, soft-templating and emulsion-based approaches have been developed as scalable alternatives for macropore generation. In these systems, macroporosity is introduced through phase-separated domains, such as oil–water emulsions or polymer-rich/poor phase segregation, which act as dynamic templates during carbonization.^276–279^ For instance, high internal phase emulsions (HIPEs) have been widely employed to produce interconnected macroporous carbons with tunable void fractions and pore sizes determined by droplet packing and stabilization conditions.^280^ These approaches eliminate the need for postsynthetic template removal and allow continuous processing, making them attractive for large-scale production. Nevertheless, compared to hard templating, soft-template-derived macropores typically exhibit broader size distributions and less structural ordering, which can compromise precise structure–property correlations.

Beyond templating routes, aerosol-assisted, foaming, and self-assembly strategies provide additional pathways to construct macroporous carbons with hierarchical architectures. Techniques such as spray drying, gas foaming, and template-free phase separation generate macropores through bubble formation, solvent evaporation, or decomposition of sacrificial components during pyrolysis.^281–288^ These methods often produce disordered yet highly interconnected macroporous networks, which can be readily integrated with micro/mesoporous domains to form hierarchical structures. Importantly, such approaches are inherently scalable and compatible with industrial processing but typically sacrifice structural uniformity and precise pore control. Therefore, a key design challenge lies in balancing scalability with structural precision, particularly when integrating macropores with smaller pore regimes to optimize the ion transport and charge storage in practical electrochemical devices.

##### 4.1.3.2 Macropores as Transport-Enabling and Charge-Active Domains in Supercapacitors

From a functional perspective, macropores play a fundamentally enabling role in governing ion transport and rate performance in carbon-based supercapacitors, particularly under practical conditions involving thick electrodes and high mass loading. Unlike micropores that primarily serve as charge storage sites through electric double-layer formation, macropores function as ion-buffering reservoirs and low-resistance transport highways, significantly reducing diffusion limitations across the electrode thickness. Early demonstrations of three-dimensional macroporous graphene frameworks revealed that introducing interconnected macropores markedly enhances both rate capability and capacitance retention by facilitating efficient ion penetration and uniform utilization of active materials throughout the electrode (Figure 18B).^102^ Building on these observations, macropores can be more generally understood as ion-buffering reservoirs and low-resistance transport highways that significantly reduce diffusion limitations across the electrode thickness. Notably, recent operando studies further suggest that, under realistic conditions involving bulky solvated ions or high mass loading, charge storage can predominantly occur within macropores rather than micropores, owing to their ability to accommodate solvated ion complexes and sustain rapid ion exchange dynamics (Figure 18C).^143^ In realistic devices (>10 mg cm^–2^), ion transport is no longer confined to nanoscale diffusion but becomes increasingly dominated by mesoscale and macroscale pathways, where insufficient macroporosity leads to severe ion depletion, concentration gradients, and utilization loss of internal surface area.^289^ Consequently, the introduction of interconnected macropores can effectively shorten ion diffusion distances, homogenize electrolyte distribution, and mitigate transport bottlenecks, thereby preserving rate capability at high current densities.

Mechanistically, the role of macropores can be understood as a transition from diffusion-limited to transport-enabled charge storage, where hierarchical pore architectures decouple the ion storage and ion transport functions. In such systems, micropores provide high capacitance via ion confinement and partial desolvation, mesopores facilitate ion exchange and intermediate diffusion, and macropores serve as electrolyte highways that sustain rapid ion flux under dynamic charging conditions. This multiscale synergy becomes particularly critical in thick electrodes, where the absence of macropores results in nonuniform charge distribution and delayed ion replenishment in the inner regions. Importantly, the benefit of macropores is not simply increasing pore volume but optimizing connectivity and percolation pathways to ensure continuous ion-accessible networks throughout the electrode. Excessive or poorly integrated macroporosity, however, can reduce the volumetric capacitance and compromise mechanical integrity, highlighting a fundamental tradeoff between transport enhancement and packing density.

These insights lead to a set of practical design rules for integrating macroporosity into high-performance carbon electrodes. First, macropores should be interconnected and hierarchically coupled with micro/mesopores rather than isolated voids, ensuring efficient ion transport across multiple length scales. Second, the volume fraction of macropores must be carefully optimized to balance ion accessibility with the volumetric energy density, particularly for compact device configurations. Third, the spatial distribution of macropores should be engineered to match ion transport gradients, for example, through gradient porosity or directional channels that facilitate electrolyte penetration in thick electrodes. Finally, scalable synthesis strategies must be considered, as highly ordered macroporous structures (e.g., inverse opals) may not translate effectively to industrial electrode fabrication. Collectively, these principles emphasize that macropores are not merely structural features but critical functional components that enable the transition from laboratory-scale performance to practical energy storage devices.

#### 4.1.4 Hierarchically Porous Carbon

Hierarchically porous carbons, defined by the integration of micro-, meso-, and macropores within a single framework, have emerged as an effective strategy to overcome the intrinsic limitations of single-pore systems in electrochemical energy storage. These architectures combine the high charge storage capability of micropores with the rapid ion transport enabled by meso- and macropores, thereby improving both energy and power performance. However, while hierarchical porosity improves ion transport, excessive macroporosity may significantly reduce electrode density, thereby compromising the volumetric energy density.

By coupling multiple pore regimes, hierarchical structures establish continuous ion transport pathways and improve the accessibility and utilization of the internal surface area. Importantly, their advantage does not arise simply from increased porosity but from the coordinated interplay between transport and storage across different length scales. Therefore, the rational design of hierarchically porous carbons lies in controlling both the pore size distribution and pore connectivity to balance capacitance and rate capability. In the following section, we summarize the representative synthetic strategies for constructing hierarchically porous carbon materials.

##### 4.1.4.1 Template-Assisted Strategies

Template-assisted synthesis remains one of the most powerful and structurally precise approaches for constructing hierarchically porous carbons with well-defined architectures spanning multiple length scales. In these systems, sacrificial templates act as spatial scaffolds that dictate the pore size, geometry, and connectivity. Typically, rigid templates such as silica colloids, ordered mesoporous silica, polymer spheres, or inorganic salts define meso- and macroporous frameworks, whereas microporosity is introduced through precursor chemistry, carbonization-induced shrinkage, or postsynthetic activation.^290,293–297^ This decoupled control over different pore regimes enables a level of structural precision that is difficult to achieve via self-templating or activation-based routes.

A key advancement in this field is the development of cooperative (dual) templating strategies, where hard templates are integrated with soft structure-directing agents to simultaneously regulate multiple pore scales. A representative example was reported by Zhao and co-workers, who combined silica colloidal crystals with amphiphilic triblock copolymers (PEO–PPO–PEO, e.g., F127) to construct hierarchically ordered macro/mesoporous carbons (Figure 19A). In this system, evaporation-induced self-assembly occurs within the interstitial voids of the colloidal crystal, while the rigid silica framework suppresses structural shrinkage during carbonization. As a result, well-defined macropores (typically 230–430 nm) are interconnected with ordered mesoporous channels (∼11 nm), forming a continuous hierarchical network. Such cooperative templating provides precise spatial control over pore architecture while maintaining structural integrity across multiple length scales.^290^ To overcome the limitations associated with silica nanocasting, including hazardous HF etching and process complexity, alternative silica-free dual-templating strategies have been developed. For example, Andreas Stein and co-workers employed polymer colloidal crystals (e.g., PMMA) as macropore templates in combination with block copolymer surfactants to direct mesostructure formation.^294^ This approach eliminates the need for silica removal, simplifies processing, and enables independent tuning of mesostructural ordering, while improving the mechanical robustness of the resulting carbon monoliths.

**Figure 19:**
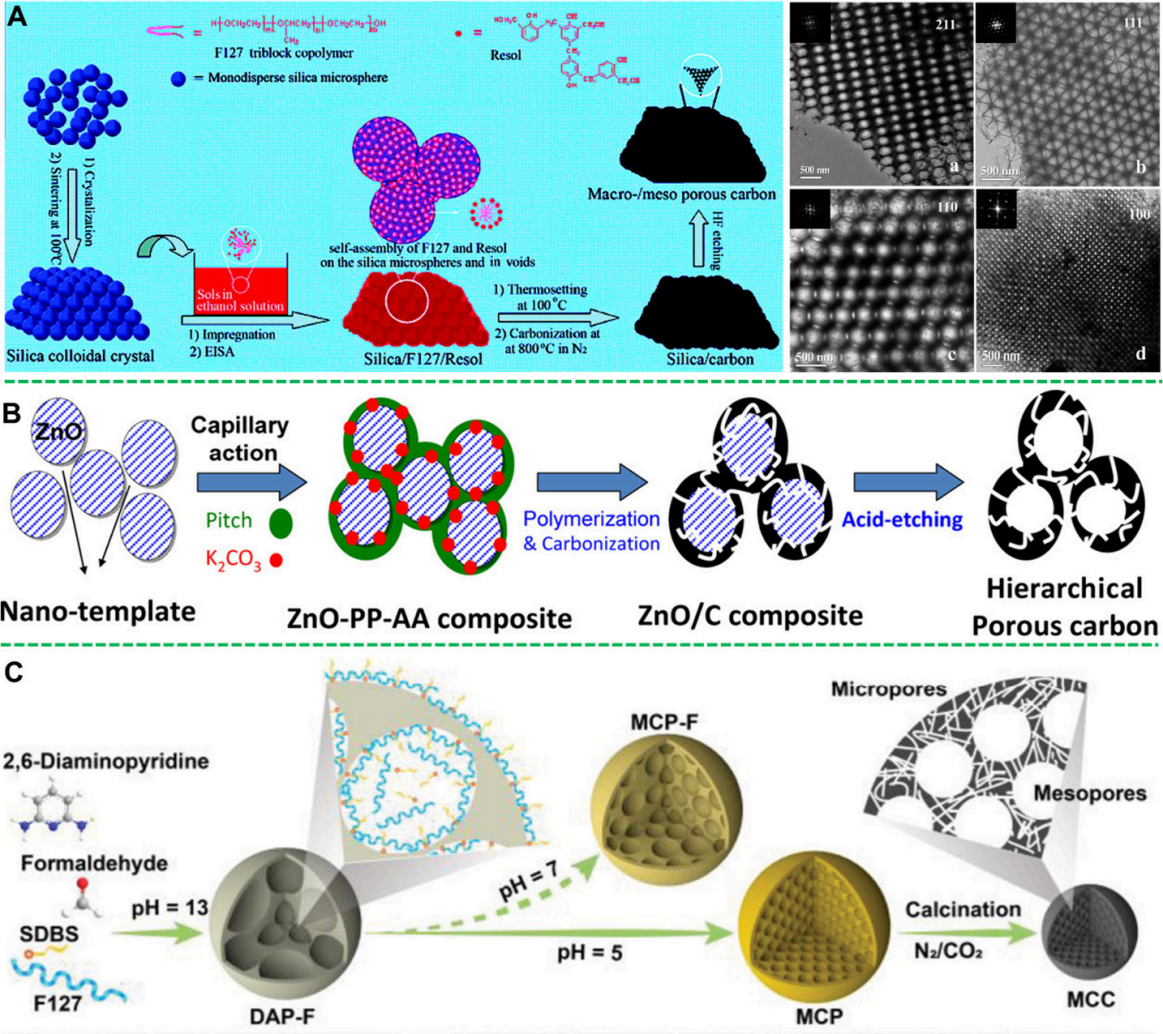
A) Synthesis scheme used for the preparation of the ordered macro-/mesoporous carbons using monodisperse silica colloidal crystals and triblock copolymer F127 as dual templates and phenolic resols as sol precursors, and TEM images of ordered macro-/mesoporous carbons with macropore arrays viewed from (211), (111), (110), (100) planes, respectively.^290^ Reproduced with permission from ref 290. Copyright 2007 American Chemical Society. B) Micro/mesoporous carbon prepared via a solvent-free method using ZnO template.^291^ Reproduced with permission from ref 291. Copyright 2017 American Chemical Society. C) Schematic illustration of a space-confined polymerization strategy to synthesize N-doped multichamber carbon (MCC) microspheres with microporous shells.^292^ Reproduced with permission from ref 292. Copyright 2019 Wiley.

Beyond classical silica- and polymer-based systems, recent efforts have increasingly focused on scalable and environmentally benign templating routes.^298^ For instance, metal oxide nanoparticles such as ZnO have been explored as hard templates in solvent-free systems, where low-cost carbon precursors (e.g., petroleum pitch) are physically mixed, carbonized, and subsequently treated with mild acids for template removal (Figure 19B). By adjusting the ZnO-to-precursor ratio, mesoporosity can be effectively tuned, while subsequent chemical activation introduces microporosity, leading to hierarchical micro/mesoporous carbons with high specific surface areas (up to ∼1979 m^2^ g^–1^). Such materials have demonstrated promising electrochemical performance, delivering specific capacitances of up to ∼200 F g^–1^ along with good cycling stability, highlighting the feasibility of combining structural control with scalable processing.^291^ This strategy avoids the use of hazardous etchants and enables partial control over mesoporosity through template loading, while additional activation steps can introduce microporosity. Such approaches highlight the potential of template-assisted synthesis to bridge structural precision with industrial feasibility.

A conceptually distinct yet mechanistically related evolution of dual-templating strategies is space-confined polymerization, which effectively replaces removable solid templates with dynamic soft interfaces and confinement effects. For example, Wang and co-workers synthesized nitrogen-doped multichamber carbon microspheres (Figure 19C) using a dual-surfactant system (F127 and SDBS). An acid-catalyzed confined polymerization partitions large precursor chambers into small mesopores (∼19–23 nm), while subsequent CO_2_ activation generates a continuous microporous shell that interconnects these internal compartments.^292^ This strategy yields a high surface area (1797 m^2^ g^–1^) and nitrogen content (∼20 wt%) while entirely eliminating postsynthetic template removal steps, thereby representing a fundamentally different design philosophy compared to hard-templating approaches such as the ZnO-etching route described above.

Building on this confinement-enabled design concept, fully soft-templated and green processing strategies have emerged as a promising direction toward further simplifying synthesis while retaining structural tunability. Compressed CO_2_-assisted assembly, for example, has been shown to regulate micelle structure and polymerization kinetics in resorcinol–formaldehyde systems. In these systems, CO_2_ not only provides a tunable acidic environment but also penetrates hydrophobic domains to modulate micelle swelling, enabling precise control over mesopore size while simultaneously expanding micropore accessibility.^299^ This dual functionality allows for the construction of interconnected micro/mesoporous networks without the need for rigid templates, thereby extending the confinement-driven design paradigm toward more sustainable, scalable, and industrially relevant synthesis routes.

Despite these advantages, template-assisted strategies face several critical challenges. The reliance on multistep processing, template removal (often involving chemical etching), and relatively low overall yield limits their scalability. Moreover, the performance advantages observed in idealized, highly ordered structures may not directly translate to practical electrodes, where factors such as packing density, electrode thickness, and electrolyte accessibility introduce additional transport constraints. Therefore, future developments should focus on balancing structural precision with process simplicity, exploring recyclable or self-removable templates, and integrating hierarchical designs with high-density electrode architectures suitable for real-world energy storage devices.

##### 4.1.4.2 Self-Templating and Intrinsic Phase-Separation Strategies

Self-templating strategies provide a more scalable alternative by generating hierarchical porosity through intrinsic structural evolution of the precursor without the need for externally added templates. These approaches rely on phase separation, in situ formation/removal of internal species, or decomposition-driven pore formation to produce multiscale pore networks.^300^

MOF-derived carbons represent one of the most extensively studied systems in this category.^145,301–303^ During pyrolysis, the organic framework transforms into carbon while coordinated metal species evaporate, catalyze structural rearrangement, or are removed post-treatment, generating micropores from framework collapse and meso/macropores from particle aggregation or metal removal. For example, Yang et al. reported the direct carbonization of Zn-based MOFs (IRMOF series), where Zn species vaporization at high temperature (∼900 °C) created abundant ultramicropores (<0.7 nm) alongside meso/macropores formed by gas release and aggregation, yielding a hierarchical carbon with an ultrahigh surface area (∼3174 m^2^ g^–1^) and large pore volume (>4 cm^3^ g^–1^).^145^ Similarly, Cao et al. demonstrated that reducing MOF precursor size (∼600 nm) promotes the formation of interconnected hierarchical structures after pyrolysis, producing carbons with high surface area (∼1356 m^2^ g^–1^) and pore volume (∼2.72 cm^3^ g^–1^), where micropores arise from framework decomposition and Zn evaporation, while meso/macropores originate from interparticle voids, leading to enhanced ion transport and high capacitance (∼369 F g^–1^).^301^ However, such high pore volumes inevitably lead to a substantial decrease in electrode packing density. Consequently, while these materials excel in gravimetric metrics, their volumetric performance (F/cm^3^ and Wh/L) often diminishes significantly due to the excessive void space, highlighting the need to optimize density for high-energy applications.

Beyond MOF systems, transient internal templates formed in situ during carbonization provide an effective route to hierarchical porosities. For instance, glucose- and nitrogen-rich precursors can generate g-C_3_N_4_ intermediates that act as sacrificial templates, whose decomposition induces meso/macropore formation and suppresses layer restacking.^304^ This mechanism exemplifies self-templating driven by intrinsic precursor chemistry and thermal evolution.

Polymer-derived systems provide another important route, particularly those based on resorcinol–formaldehyde or related thermosetting resins.^305^ In these systems, phase separation during polymerization or carbonization leads to gel-like interconnected networks, while gas evolution during pyrolysis generates meso- and macropores. Wang et al. constructed hydrogel-derived hierarchical carbons via covalent assembly of PVA/PANI and graphene oxide, forming interconnected nanosheet networks with improved ion transport.^306^ Zang et al. developed POP-derived carbons via coupled activation and N-doping, achieving ultrahigh surface area and robust hierarchical porosity.^149^

The key advantage of self-templating lies in its synthetic simplicity and scalability. Because pore formation is intrinsically coupled to precursor chemistry, these methods are well suited for bulk production and thick electrode fabrication. However, this advantage comes at the expense of structural precision. Compared with template-assisted approaches, self-templated carbons typically exhibit broader pore size distributions and less ordered architectures, making it more challenging to establish clear and quantitative structure–performance relationships.

##### 4.1.4.3 Postsynthetic and Activation Strategies

Postsynthetic activation provides a critical route to overcome a fundamental limitation of templating strategies, namely, the difficulty in introducing well-defined microporosity into hierarchical carbon frameworks. In most template-derived systems, micropores primarily originate from the decomposition of small molecular fragments during carbonization, making their precise control inherently challenging. As a result, activation processes are often employed as a secondary step to enrich microporosity and rebalance the pore hierarchy.

Chemical activation using agents such as KOH or CO_2_ is particularly effective in generating abundant micropores while simultaneously modifying existing meso- and macroporous structures through etching and gasification.^149,307^ This dual role enables the fine regulation of pore size distribution and surface area. For example, activation of hard-templated carbons (e.g., MgO/polyurethane systems) significantly increases the micropore volume and BET surface area while preserving the overall hierarchical framework.^308^ Similarly, activation of soft-templated carbons can introduce substantial microporosity, demonstrating the general applicability of this approach across different precursor systems.^309^

Importantly, these strategies highlight a key design principle: excessive meso- and macroporosity, while beneficial for ion transport, often comes at the expense of surface area and charge-storage sites. Postsynthetic activation therefore serves as a necessary tuning step to balance micro-, meso-, and macropore fractions, enabling simultaneous optimization of accessibility and capacitance.

From a practical perspective, activation-based strategies are highly scalable and compatible with diverse precursors including synthetic polymers and biomass-derived carbons. However, the lack of structural precision remains a challenge as aggressive activation can lead to pore collapse, reduced electrical conductivity, and diminished mechanical integrity. This underscores an inherent tradeoff between pore generation and structural stability in post-treated hierarchical carbons.

##### 4.1.4.4 Bridging Synthesis and Function in Hierarchically Porous Carbons

Collectively, these synthetic strategies highlight that hierarchical porosity arises from fundamentally different design philosophies, each with distinct implications for electrochemical performance. Template-assisted methods provide model systems with precise structural control, enabling clear elucidation of structure–property relationships. In contrast, self-templating and postsynthetic strategies emphasize scalability and synthetic simplicity, often at the expense of structural uniformity.

Importantly, these differences are not merely synthetic—they directly determine pore connectivity, ion transport pathways, and the spatial distribution of charge storage sites. As a result, the performance of hierarchically porous carbons cannot be understood solely in terms of surface area or pore volume. Instead, it reflects a complex interplay between synthesis route, resulting architecture, and functional roles of pores across different length scales. This synthetic–structural–functional coupling ultimately necessitates a mechanistic framework to understand how hierarchical porosity governs ion transport and charge storage under realistic operating conditions.

##### 4.1.4.5 Multiscale Transport–Storage Coupling in Hierarchically Porous Carbons

Hierarchically porous carbons regulate electrochemical performance not by maximizing surface area alone but by redistributing ion transport and charge storage across multiple length scales.^310^ This multiscale architecture enables a transition from diffusion-limited to transport-enabled charge storage, which becomes particularly critical under practical conditions involving thick electrodes, high mass loadings, and solvated ions.

In conventional microporous carbons, charge storage is dominated by ion confinement within subnanometer pores, often requiring partial desolvation and resulting in sluggish transport kinetics. Consequently, only a fraction of the internal surface area is effectively utilized at high rates. The introduction of meso- and macropores fundamentally alters this behavior by creating continuous ion-transport pathways that penetrate the electrode interior. Within this framework, micropores act as charge storage sites, mesopores mediate ion exchange between domains, and macropores function as low-resistance transport highways and ion buffering reservoirs (Figure 20).

**Figure 20:**
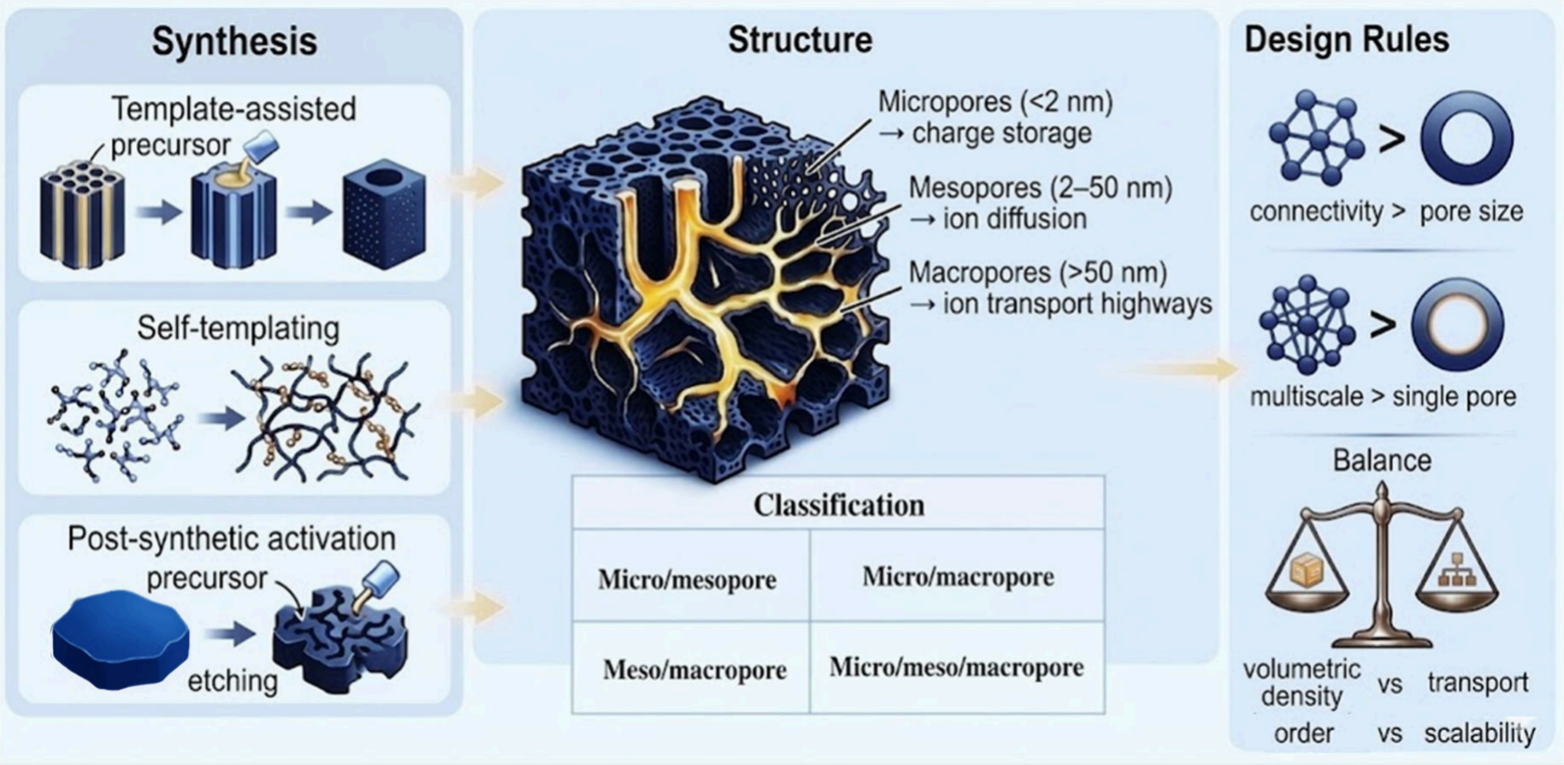
Synthesis, structure and design rules of hierarchically porous carbon materials.

Importantly, these pore regimes do not operate independently but form a coupled transport–storage network. The effectiveness of hierarchical porosity therefore depends less on the absolute size of individual pores than on their connectivity and spatial integration. Poorly connected pores, even if optimally sized, can become electrochemically inactive, whereas percolated macroporous networks ensure uniform electrolyte access and mitigate ion depletion in thick electrodes. Under such conditions, charge storage can partially shift toward larger pores, highlighting that the dominant storage mechanism is not fixed but evolves with transport constraints.

This perspective leads to a key design principle: hierarchical porosity should be understood as a functional network rather than as a structural sum of pores. Optimizing the performance requires balancing three competing factors. First, connectivity must be prioritized over pore size to maintain continuous ion pathways. Second, multiscale integration must be preserved to decouple the transport and storage functions across different pore regimes. Third, the pore volume must be balanced against electrode density, as excessive macroporosity improves transport but compromises volumetric capacitance and mechanical integrity.

Furthermore, the optimal pore architecture is inherently context-dependent. Under realistic operating conditions—such as high mass loading, concentrated electrolytes, or large solvated ions—ion transport increasingly relies on mesoscale and macroscale pathways. As a result, structures that perform well in thin electrodes may fail when scaled, emphasizing that hierarchical design must be aligned with device-level constraints rather than idealized metrics.

Taken together, these insights redefine hierarchical porosity from a passive structural feature to an active design parameter that governs the interplay among ion transport, charge storage, and electrode utilization. This framework shifts the focus from maximizing the surface area to engineering pore connectivity and multiscale integration, providing a mechanistic basis for designing carbon electrodes that perform reliably under practical conditions.

#### 4.1.5 Hollow Carbon

Hollow carbon materials (HCMs), featuring unique internal cavities and tunable shell architectures, have demonstrated outstanding performance across a wide range of frontier applications, including energy storage, catalytic conversion, and biomedicine. Their distinctive characteristics—high specific surface area, low density, excellent chemical stability, good electronic conductivity, and efficient mass/charge transport pathways—position HCMs as representative candidates for next-generation functional materials.

With the rising demand for multifunctional integration, hollow carbon structures have evolved from simple single-shelled spheres to more complex architectures with multishells, multichambers, heterointerfaces, and functional moieties. This structural evolution not only reflects the advancement in material design capabilities but also indicates a deeper understanding of synthetic mechanisms. According to the fabrication principles, current synthetic strategies for HCMs can be broadly classified into three categories: hard-template, soft-template, and template-free (self-templated) approaches, each offering distinct advantages in structural control and applicability.

##### 4.1.5.1 Hard-Template Method

The hard-template strategy is the most straightforward and widely adopted route, akin to the synthesis of traditional ordered mesoporous carbons. In this approach, rigid nanostructures—such as silica or metal oxides—are coated with a carbon precursor (e.g., phenolic resin or polypyrrole), followed by carbonization and template removal via chemical etching. The main advantage lies in its high predictability and controllability over size and morphology, making it suitable for large-scale production of monodisperse hollow carbon spheres (HCSs). Silica spheres remain the most frequently used hard templates, owing to their excellent thermal stability and tunable particle sizes, which are well-suited for constructing multishell or gradient cavity structures. For instance, Dai et al. achieved subnanometer-level control over carbon shell thickness by modulating polydopamine (PDA) deposition and pH (Figure 21A), leading to uniform HCSs with superior rate capabilities in electrochemical energy storage.^311^ In addition to inorganic templates, organic ones such as polystyrene (PS) spheres have attracted attention (Figure 21B) since they can be removed by pyrolysis or solvent dissolution, thereby avoiding acid etching that may damage carbon shells.^312^ Gil-Herrera et al. developed a PS–glucose system that allowed simultaneous shell formation and template removal during hydrothermal carbonization, improving both efficiency and structural integrity.^317^ Green templates such as CaCO_3_ ^312,317–319^ metal^320,321^ and metal oxides^322,323^ have also been introduced for their mild removal conditions and ability to induce porosity or dopant incorporation during carbonization. For example, CO_2_ released during CaCO_3_ decomposition can in situ activate carbon shells, generating porous structures that enhance catalytic and storage performance. Despite its precise replication capability, the hard-template method often involves multistep processes—coating, etching, and washing—and faces challenges in environmental friendliness and template recyclability, which limit its scalability.

**Figure 21:**
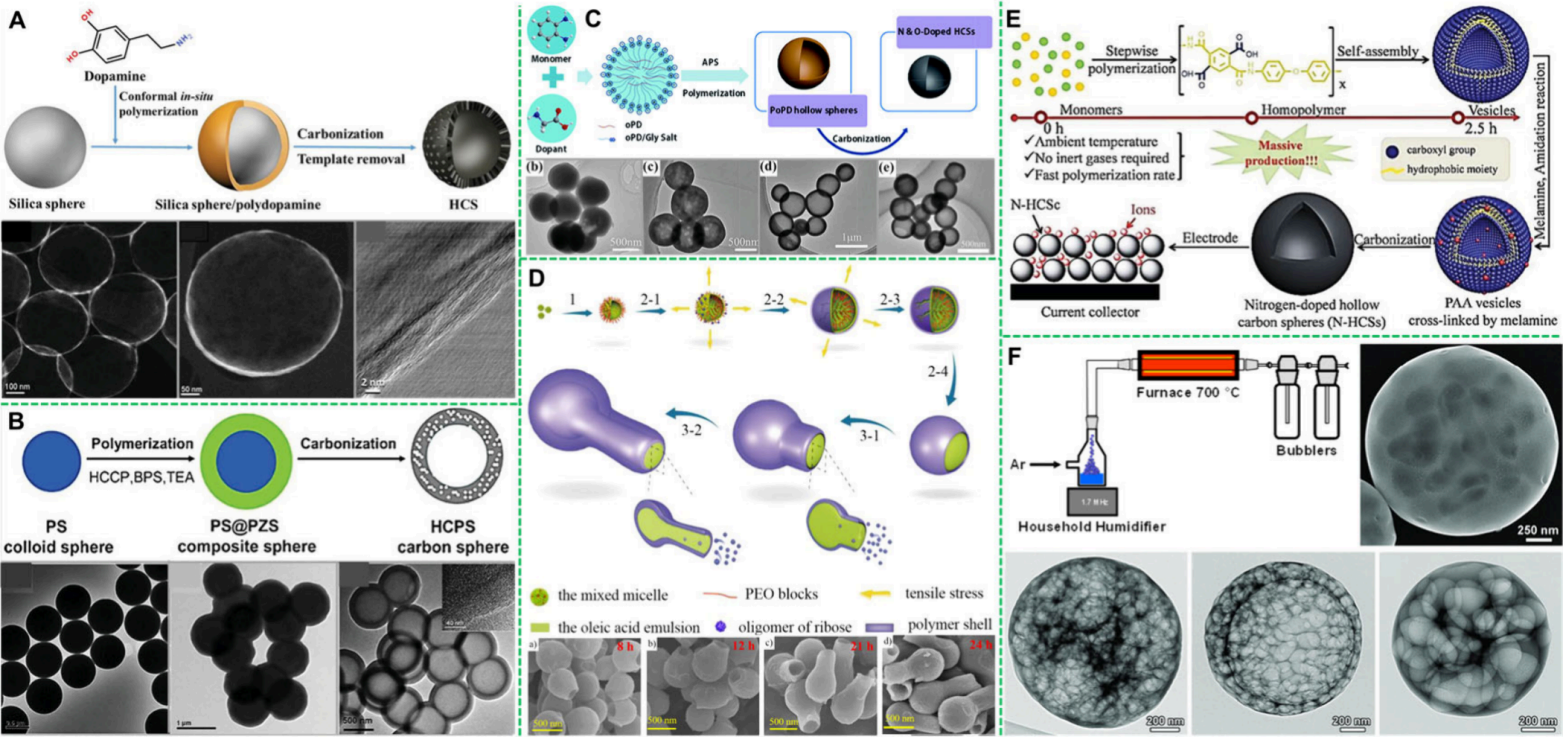
Schematic illustration of the synthesis of hollow carbon spheres (HCS) by hard-template: A) silica sphere;^311^ Reproduced with permission from ref 311. Copyright 2011 Wiley. B) PS sphere.^312^ Reproduced with permission from ref 312. Copyright 2010 Royal Society of Chemistry. Schematic illustration for the synthesis of HCS by soft templating. C) A schematic illustration depicting the synthesis route for N- and O-doped hollow carbon spheres derived from poly­(*o*-phenylenediamine).^313^ Reproduced with permission from ref 313. Copyright 2015 Royal Society of Chemistry. D) Asymmetric flasklike hollow carbonaceous nanoparticles fabricated by the synergistic interaction between soft template and biomass.^314^ Reproduced with permission from ref 314. Copyright 2017 American Chemical Society. E) Scalable fabrication of poly­(amic acid) (PAA) homopolymers and its subsequent self-assembly into vesicles and carbonization into N-HCS.^315^ Reproduced with permission from ref 315. Copyright 2016 Royal Society of Chemistry. F) Synthesized porous carbon spheres by ultrasonic spray pyrolysis (USP) using either halogenated carboxylate salts or simple carbohydrates via a continuous, one-step, and template-free aerosol approach.^316^ Reproduced with permission from ref 316. Copyright 2012 Wiley.

##### 4.1.5.2 Soft-Template Method

The soft-template method leverages dynamic self-assembly systems—such as surfactants, emulsions, or polymer micelles—under interface-guided coassembly. These systems undergo phase separation and interface modulation, synergistically inducing cavity formation and shell construction during carbonization. This strategy eliminates the need for external template removal, offering simpler processes and greater flexibility in introducing functionalities. It is especially suitable for fabricating porous, doped, or gradient-structured HCMs. A representative example is the PoPD micelle system (Figure 21C), where positively charged organic microspheres self-assemble from o-phenylenediamine (o-PD) and glycine in acidic media. During pyrolysis, they form N,O codoped HCSs.^313^ Yuan et al. demonstrated that such heteroatom-doped hollow carbons possess abundant accessible active sites and favorable charge-transfer characteristics, enabling high electrochemical sensitivity and promising performance in electrochemical sensing and detection applications.^324^ Metal–organic frameworks (MOFs), particularly nitrogen-rich ZIF-8, also show promise as soft templates.^325–327^ Upon pyrolysis, the inner MOF core decomposes to generate a cavity while the outer layer carbonizes into a nitrogen-doped shell with abundant active sites. Yang et al. demonstrated ZIF-8-derived HCSs with excellent performance in catalysis and lithium-ion batteries.^328^ Furthermore, Pickering emulsion methods utilize particle stabilizers (e.g., SiO_2_ or lysine) to stabilize water–oil interfaces, guiding the deposition of carbon precursors at these boundaries to form multishell or anisotropic HCSs.^329,330^ Liu et al. tuned interfacial behaviors to construct hierarchical cavities and multiscale pore networks, enhancing ion transport for energy storage applications.^329^ Chen et al. reported a soft-template/biomass coassembly approach (Figure 21D), where asymmetric flask-shaped hollow carbon nanostructures were synthesized using P123 and sodium oleate in the presence of ribose. They proposed a dynamic “nano-droplet filling and evacuation” mechanism to explain the formation of a highly accessible, contracted hollow interior with an exceptionally high surface area (∼2300 m^2^ g^–1^).^314^ Nevertheless, soft-template systems often suffer from poor stability and are highly sensitive to parameters like pH, temperature, and concentration, posing challenges for scalable and reproducible synthesis.

##### 4.1.5.3 Self-Template Method

Self-templating strategies, which eliminate external templates, rely on the intrinsic evolution of precursors and reaction-induced phase separation to spontaneously construct hollow structures. This approach integrates sustainability, efficiency, and structural-functionality synergy, making it a promising route for scalable HCM production. Polymer vesicles, formed from macromolecules such as poly­(acrylic acid) (PAA) or polyethyleneimine (PEI), are common self-templates. These vesicles can be carbonized into HCSs with tunable architectures (Figure 21E).^315^ By adjusting the polymer molecular weight, cross-linkers, or pH, one can finely tune shell thickness and surface functionalities. Melamine–formaldehyde resin spheres serve as another effective system. Upon heating, the outer layer carbonizes first, while the inner core decomposes and releases gas, forming an “inside-out” cavity. Ma et al. used this strategy to synthesize porous HCSs with outstanding high-rate performance and cycling stability in lithium-ion batteries.^331^ Among self-templated approaches, spray pyrolysis stands out for industrial scalability. Precursor solutions are atomized and rapidly dried, condensed, and carbonized in high-temperature environments, where gas evolution induces cavity formation while constructing porous shells. Suslick et al. realized high-throughput synthesis of HCSs using this method, with excellent energy storage performance and cost-effectiveness (Figure 21F).^316^ However, challenges remain in achieving uniform morphology and precise control, particularly for multichamber or heterostructured HCMs, necessitating deeper mechanistic insights.

##### 4.1.5.4 Structural Modulation of Hollow Carbon

The performance of HCMs in practical applications is intrinsically tied to their structural features. Researchers have developed multidimensional strategies to modulate morphology—ranging from template design to kinetic control and hierarchical assembly—thus vastly expanding the structural and functional diversity of HCMs. 1) Template diversification for enhanced architectural freedom: Traditional silica templates can be modified by adjusting the TEOS/TPOS ratio to tune cavity size within the mesoporous range (Figure 22A).^332^ By tuning the TEOS-to-resol ratio and the aging time of the silica precursor, Zhao et al. realized ordered mesoporous carbon frameworks with adjustable mesopore sizes and bimodal pore structures, highlighting the versatility of silica-based templating for pore architecture control.^333^ Droplet-based templates, such as xylene droplets, were employed in Chen et al.’s “nano-pottery” method to dynamically deposit C_60_ at the droplet interface, yielding bowl- and gourd-shaped HCSs (Figure 22B), offering new pathways to multichamber and interface-rich designs.^153^ Beyond these rigid templates, dual-templating strategies combining colloidal silica with diblock copolymer micelles (e.g., PS-b-PEO) have enabled the formation of nitrogen-doped hollow carbon spheres with unusually large (∼20 nm) and tunable mesoporous shells, as demonstrated by Tang et al. The synergistic hard/soft templating expands the accessible pore sizes and enriches the surface chemistry simultaneously.^334^ 2) Precursor-driven cavity evolution: Wan et al. developed a 3-aminophenol/formaldehyde copolymer precursor (Figure 22C),^152^ where molecular weight gradients within particles guided selective dissolution in acetone to create internal cavities. Carbonization then produced HCSs with controllable shell numbers, thicknesses, and mesoporosity for enhanced surface area and active sites. Complementing these chemistry-driven routes, soft-templated hydrothermal assembly has also been leveraged to create unique asymmetric hollow geometries. Zhang et al. reported bowl-like hollow carbon spheres (BHCSs) that form through cooperative hydrothermal carbonization and soft templating, producing well-defined 1–2 μm bowl morphologies with efficient ion-accessible interiors and promising electrochemical double-layer capacitor performance.^335^ 3) Structure–performance coupling: The electrochemical and functional performance of hollow carbon materials (HCMs) is closely dictated by the interplay between their shell architecture, pore hierarchy, and geometric anisotropy. Multishell architectures shorten ion diffusion pathways and buffer mechanical stress, enabling fast-charging behavior in high-power battery systems.^336^ Meanwhile, large mesopores significantly enhance ion transport and electrolyte infiltration, thereby improving capacitor response rates. Beyond these classical hollow motifs, multichamber configurations and anisotropic geometries—such as bowl-like or gourd-shaped shells—further increase the accessibility of active surfaces and provide spatial domains for the selective loading of catalysts or therapeutic cargos, offering unique advantages in cascade catalysis and controlled drug delivery. Collectively, these structural features highlight morphology as a decisive design handle for optimizing ion transport, mass accessibility, and multifunctional performance.

**Figure 22:**
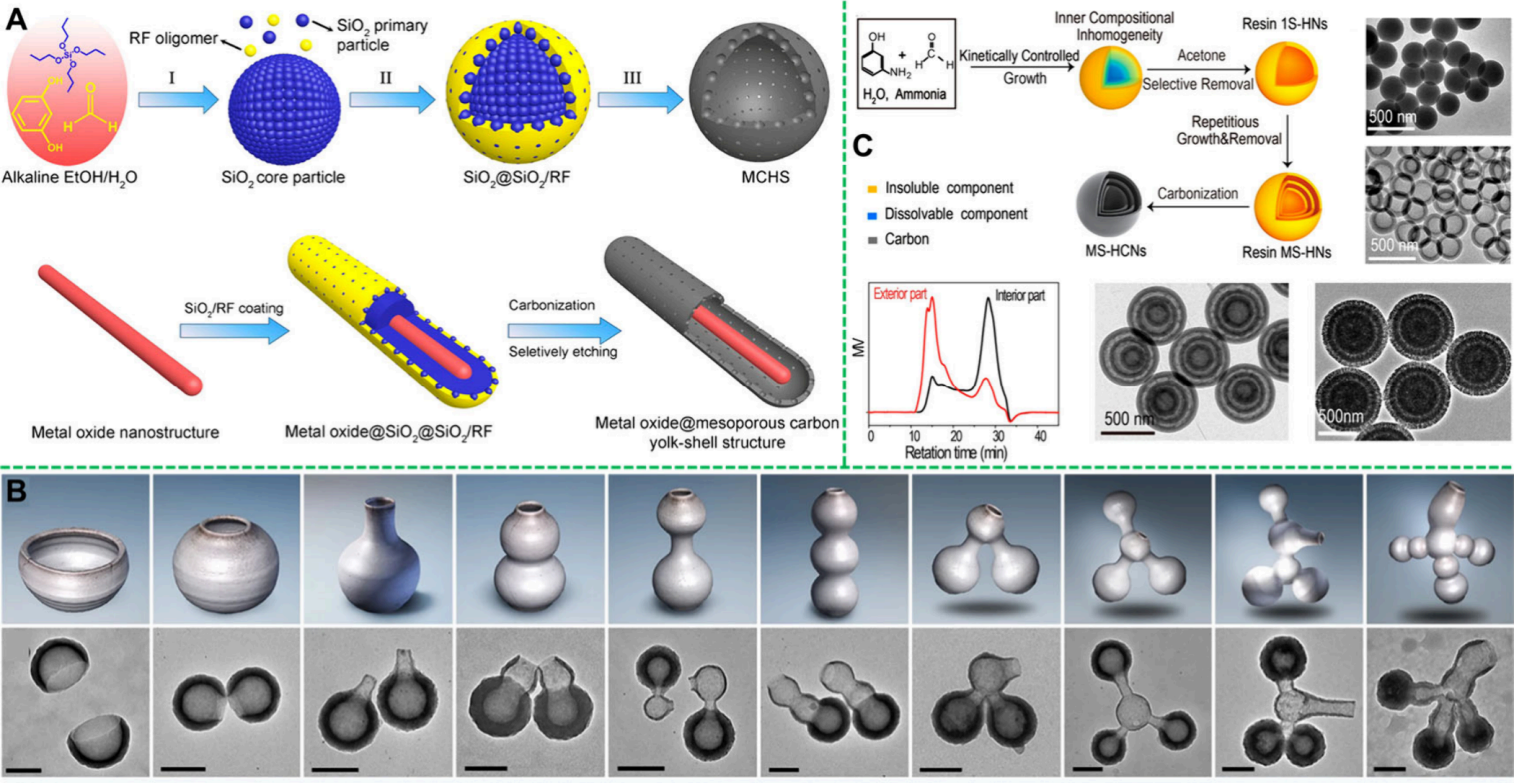
A) A schematic illustration of the one-pot, surfactant-free synthesis of mesoporous carbon hollow spheres.^332^ Reproduced with permission from ref 332. Copyright 2016 American Chemical Society. B) Schematic illustration of the synthesis of fullerene hollow nanostructures with tailored shapes (bowls, bottles, and cucurbits) by exploiting the liquid nature of the xylene template.^153^ Reproduced with permission from ref 153. Copyright 2019 Springer Nature. C) Schematic illustration of the synthesis of multishelled hollow nanospheres (MS-HNs) via kinetically controlled growth.^152^ Reproduced with permission from ref 152. Copyright 2017 American Chemical Society.

Looking forward, future advances will rely on the integrated regulation of morphology, functionality, and interfacial chemistry. Dynamic templating systems (e.g., self-assembled aminophenol–formaldehyde resins or soft/hard dual-templating micelle–silica hybrids) are expected to enable simultaneous programming of complex geometries, heteroatom doping, and hierarchical porosity. In situ characterization tools—including in situ TEM, photothermal imaging, and operando spectroscopies—will provide real-time insight into cavity formation and shell reconstruction, guiding the precise engineering of hollow structures. Moreover, carbon-based composites (e.g., metal oxide@carbon, C_60_-derived shells, or polymer-derived N-doped carbons) will play a pivotal role in expanding the dimensionality, surface reactivity, and interfacial functionality of next-generation HCMs.

##### 4.1.5.5 Hollow Carbon for Fast-Charging

Structurally, hollow carbons present a unique combination of low density, high specific surface area, and short ion diffusion pathways. Compared to microporous or purely mesoporous carbons, hollow structures facilitate rapid electrolyte penetration and ion transport not only through the external surface but also across the internal void space, significantly enhancing rate capability. This dual-interface accessibility, arising from both outer shell and inner cavity, enables more efficient ion flux distribution under high-rate operation. Furthermore, sophisticated hollow architectures such as Janus and core–shell structures can be engineered via interfacial self-assembly or dual-template strategies, offering enhanced functionality and stability for high-rate applications.^340^ Their intrinsic advantages have been demonstrated in a number of representative systems. For instance, Xu et al. demonstrated that ultrahigh-surface-area hollow carbon nanospheres (up to ∼2200 m^2^ g^–1^) provide fast ion accessibility and outstanding capacitive performance owing to the interconnected hollow cavity and thin shell structure (Figure 23A).^337^ Similarly, Tang et al. showed that hollow carbon nanospheres exhibit exceptional rate capability even for sluggish Na^+^ transport (Figure 23B),^339^ underscoring the universality of hollow architectures in boosting ion kinetics in both Li- and Na-based systems. The hollow cavity also acts as an ion-buffering reservoir under high current densities, while the thin carbon shell ensures minimal diffusion resistance. Such a reservoir effect helps alleviate transient ion depletion and reduces concentration polarization during rapid charging/discharging processes. However, this thin shell configuration often compromises mechanical robustness, leading to structural collapse or pulverization during prolonged cycling.^341^ To enhance structural stability, commonly employed strategies include constructing multilayer or reinforced shells and introducing heterostructure modifications.^342^ In addition, Li et al. demonstrated that ultrathin yet mechanically stable hollow carbon shells derived from petroleum asphalt—only ∼4.7 nm thick—can simultaneously promote rapid ion transport and suppress shell degradation (Figure 23C), illustrating the critical role of precisely engineered shell thickness in achieving both high rate performance and long-term durability.^338^

**Figure 23:**
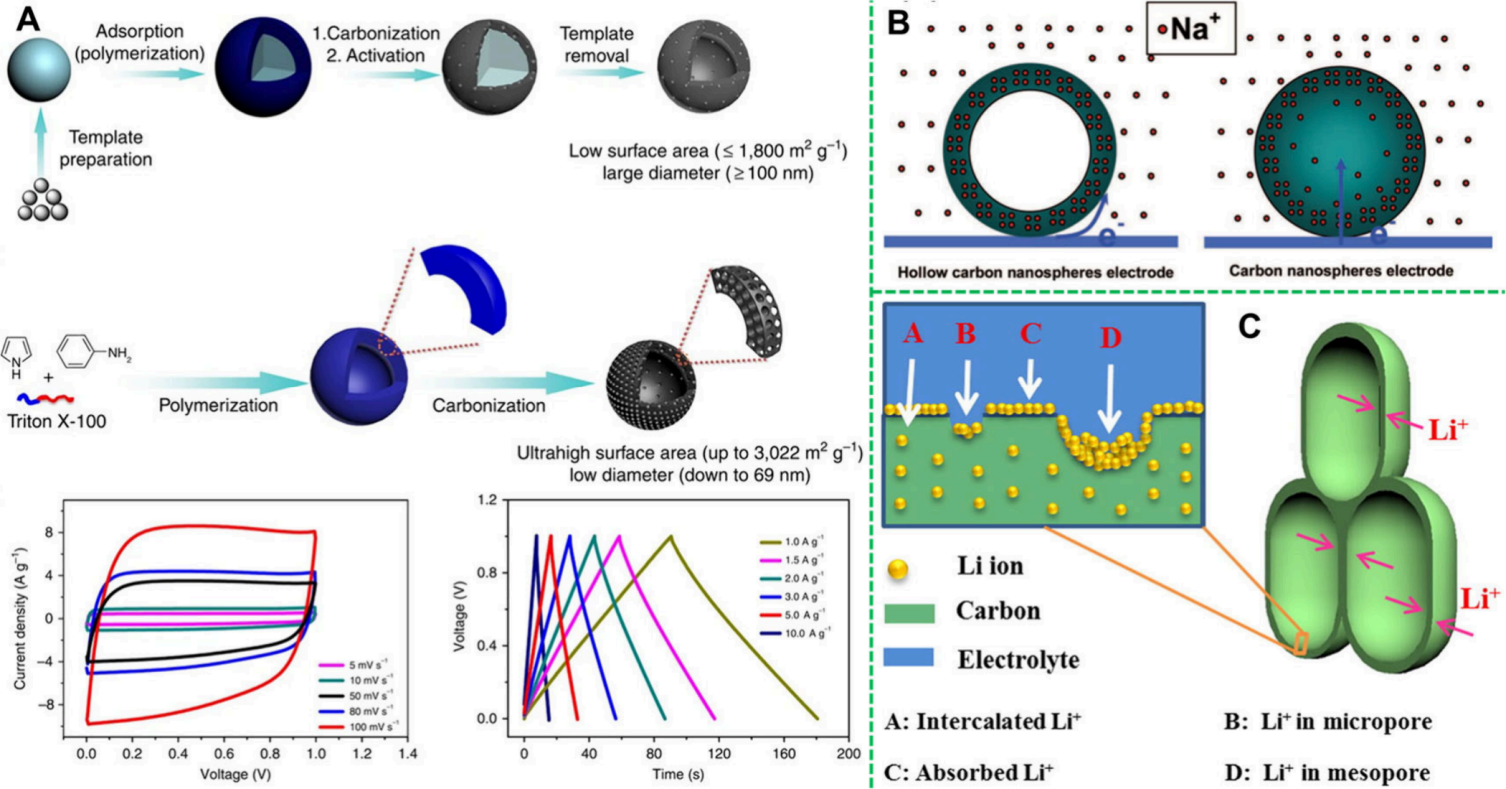
A) Schematic illustration of preparation of Hollow carbon nanospheres (HCNs) and supercapacitive performances.^337^ Reproduced with permission from ref 337. Copyright 2015 Springer Nature. B) Schematic of lithium storage and transport in petroleum asphalt based ultrathin hollow carbon shell (PACS).^338^ Reproduced with permission from ref 338. Copyright 2016 Elsevier. C) Schemes of the electrochemical reaction process of hollow carbon nanospheres and carbon spheres.^339^ Reproduced with permission from ref 339. Copyright 2012 Wiley.

Despite these advantages, the synthesis complexity and difficulty in achieving uniform shell thickness remain challenges, particularly for large-scale production.^343–345^ Emerging approaches, such as precision pore engineering via atomic layer deposition (ALD) coating, dual-template assembly, and heteroatom doping strategies, are being explored to reinforce shell stability and introduce hierarchical micro/mesopores into the shell walls, thus combining the merits of hollow and conventional porous architectures.^346,347^

In the context of fast-charging applications, hollow carbon’s advantages over purely microporous or mesoporous carbons are pronounced. Microporous carbons provide abundant surface area but suffer from sluggish ion diffusion, while mesoporous carbons offer faster kinetics yet often lack sufficient active surface for high charge storage. Hollow carbons, integrating internal cavities with tunable micro/mesoporous shells, effectively mitigate the tradeoff between ion-transport speed and charge-storage density. This multiscale porosity not only supports ultrafast ion kinetics but also buffers transient ion fluxes and accommodates volumetric changes during cycling, making hollow carbon one of the most promising architectures for next-generation fast-charging supercapacitors and battery electrodes.

Beyond these structure–performance correlations, hollow carbon architectures introduce an additional level of structural optimization by fundamentally decoupling ion transport pathways from charge-storage domains. The internal void space shortens effective diffusion distances and enhances electrolyte accessibility throughout the electrode, while simultaneously serving as an ion-buffering reservoir that sustains rapid charge propagation. Compared to dense porous frameworks, such architectures exhibit reduced transport resistance and can maintain high rate capability even at increased electrode thickness, which is particularly advantageous for practical device configurations. However, their intrinsically low packing density and potential structural instability may limit volumetric energy density. Therefore, hollow structures are most effective when integrated with hierarchical porosity, where the hollow framework accelerates ion transport and micropores embedded within the shell provide efficient charge-storage sites. From a structural design perspective, hollow carbons thus function as an intermediate yet critical bridge between mesoporous and fully hierarchical systems.

#### 4.1.6 Hyperporous Carbon

Hyperporous carbons represent a unique class of SPCs characterized by exceptionally high porosity and specific surface area, exceeding the theoretical limit of single-layer graphene (2630 m^2^·g^–1^). From a design-principle perspective, the key advantage of hyperporous carbons is not merely their high surface area, but their ability to simultaneously integrate charge storage domains (micropores) and rapid ion transport pathways (meso/macropores), thereby addressing the intrinsic tradeoff between energy density and rate capability observed in single-scale porous systems. These materials have emerged as promising candidates for next-generation fast-charging energy storage systems. However, rather than the specific precursor chemistry itself, it is the resulting rigid, highly cross-linked framework that governs performance: such structures effectively suppress pore collapse during pyrolysis, enabling retention of ultrahigh porosity and interconnected transport networks. They are commonly synthesized via carbonization of hypercrosslinked polymers derived from monomers such as benzene, pyrrole, thiophene, and more recently, triptycene derivatives. The intrinsic rigidity and interconnected network structure of hypercrosslinked polymers enable effective structural retention during pyrolysis, yielding hyperporous carbons with finely tunable pore architectures and heteroatom doping. This highlights a generalizable design rule: structural rigidity at the precursor stage is critical for preserving hierarchical porosity after carbonization.

Such synthetic strategies afford carbons with interconnected microporous and mesoporous hierarchical structures, enhancing electrolyte accessibility and ion transport—key attributes for high-rate energy storage applications. Critically, the performance enhancement arises from a synergistic mechanism: micropores provide high-density charge storage via ion confinement, while meso- and macropores reduce transport resistance by enabling electrolyte penetration and buffering ion flux. The relative balance between these pore types, rather than their absolute surface area, is the dominant factor governing rate performance. Recent studies have unveiled advanced approaches to fabricate hyperporous carbons from hypercrosslinked polymers and demonstrated their multifunctional applications. For instance, N,S codoped porous carbons synthesized from pyrrole and thiophene monomers (Figure 24A) exhibit a high micropore ratio of 77.5% and a specific surface area of ∼1339 m^2^·g^–1^, delivering outstanding electrochemical performance with capacitances up to 455 F·g^–1^ and excellent cycling stability.^348^ Notably, despite a moderate surface area compared to other hyperporous systems, the high fraction of ion-accessible micropores is primarily responsible for the high capacitance, underscoring that effective pore utilization outweighs absolute surface area. Micropores act as primary charge storage sites, while meso- and macropores facilitate rapid ion diffusion; heteroatom doping enhances conductivity and pseudocapacitive contributions, collectively boosting storage capabilities. Another example involves hyperporous carbons derived from triptycene-based hypercrosslinked polymers, which show ultrahigh surface areas (up to 3125 m^2^·g^–1^) and large pore volumes (1.6 cm^3^·g^–1^) (Figure 24B).^349^ However, these ultrahigh surface areas do not necessarily translate proportionally into electrochemical performance, as excessively large pore volumes can reduce packing density and volumetric capacitance, revealing an inherent limitation of hyperporous architectures. These materials demonstrate exceptional adsorption capacities for iodine in both gas and liquid phases, indicating potential for nuclear waste treatment and environmental remediation. Their rigid, highly cross-linked structures maintain pore stability, and KOH activation induces abundant micropores.

**Figure 24:**
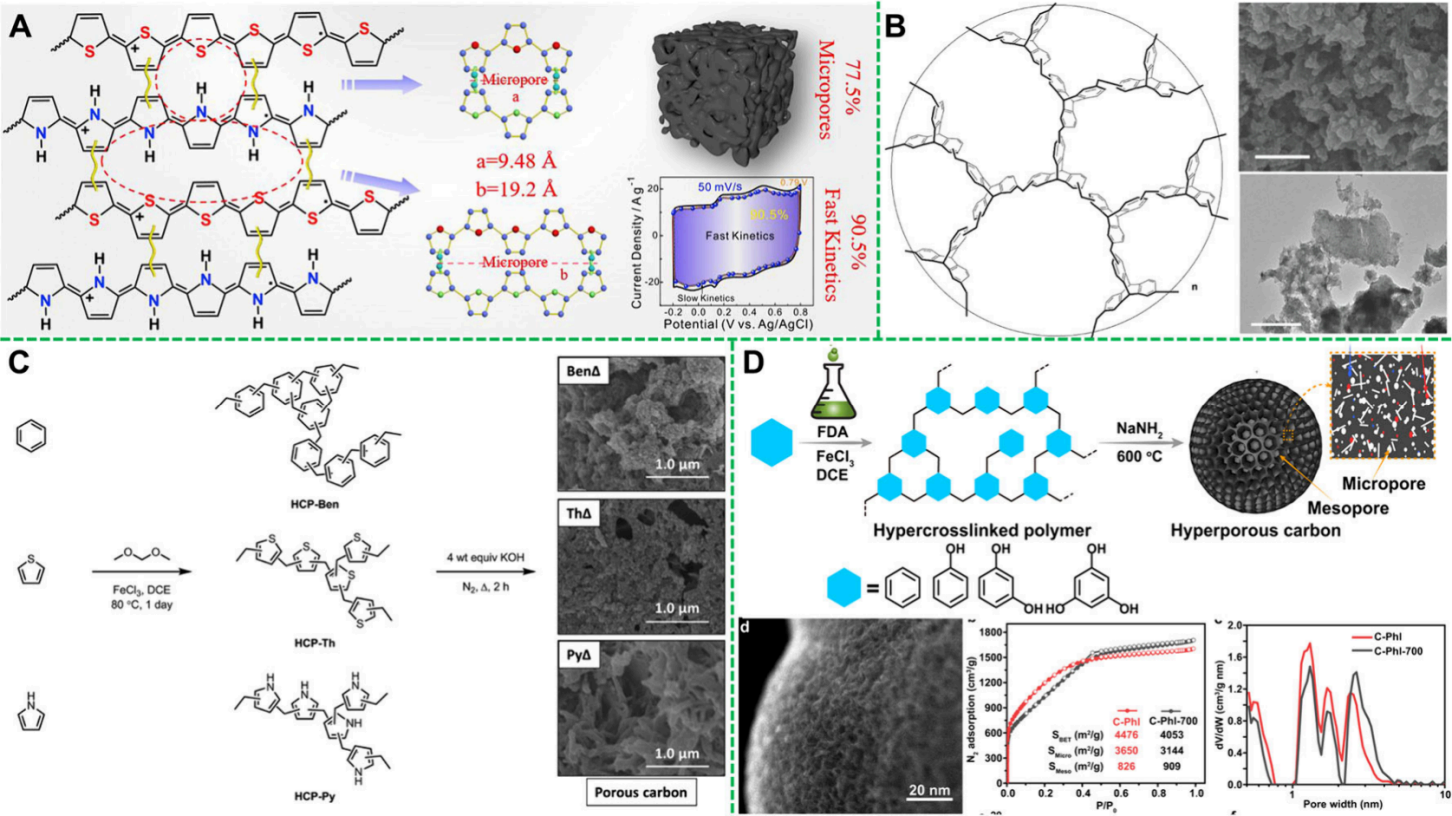
A) Synthesis of N, S-MC derived from hypercrosslinked polymerization of two individual monomers of polypyrrole and polythiophene for ultrafast electron/ion transport supercapacitors.^348^ Reproduced with permission from ref 348. Copyright 2019 Elsevier. B) Hyperporous carbon from triptycene-based hypercrosslinked polymer.^349^ Reproduced with permission from ref 349. Copyright 2019 Wiley. C) Synthesis of the hypercrosslinked polymers and the subsequent carbonization method.^27^ Reproduced with permission from ref 27. Copyright 2016 Wiley. D) Experimental design for the synthesis of the target hyperporous carbons predicted by the artificial neural network (ANN) model.^32^ Reproduced with permission from ref 32. Copyright 2023 Springer Nature.

Furthermore, pore size and structure can be finely tuned by controlling carbonization temperature and processing conditions. Hypercrosslinked polymers synthesized from benzene, pyrrole, and thiophene, followed by carbonization and KOH activation, yield porous carbons with specific surface areas up to 4334 m^2^·g^–1^, where pore architecture and heteroatom doping synergistically enhance gas adsorption selectivity and electrochemical performance (Figure 24C).^27^ Yet, aggressive activation that maximizes surface area often introduces excessive ultramicroporosity and structural defects, which can hinder ion transport under high-rate conditions and reduce initial Coulombic efficiency. Similarly, porous carbons derived from aniline monomers with NH_3_-induced nitrogen doping maintain high porosity while achieving high conductivity and pseudocapacitance, exhibiting excellent rate capability and cycling stability.^350^ Collectively, these reports demonstrate routes to construct hierarchical pore structures and multifunctional surfaces by combining hypercrosslinked polymers with high-temperature carbonization, activation, and heteroatom doping, producing porous carbons with ultrahigh surface areas and superior electrochemical activity. More importantly, they reveal that the optimal performance of hyperporous carbons originates from a multiscale design synergy—balancing pore size distribution, surface chemistry, and framework stability—rather than maximizing any single structural parameter.

Concurrently, emerging synthesis methods for hyperporous carbons have begun to surpass traditional trial-and-error approaches. For example, Wang et al. employed machine learning-assisted design (Figure 24D) to tailor polymer precursors and optimize carbonization processes, utilizing sodium amide as a pore-forming agent to prepare hyperporous carbons with surface areas exceeding 4000 m^2^·g^–1^ and outstanding performance in aqueous H_2_SO_4_ electrolytes.^32^ This data-driven strategy further emphasizes that predictive control over pore architecture and functional group distribution is more critical than empirical optimization for achieving consistent high-rate performance.

Compared with traditional microporous carbons whose ion accessibility and diffusion kinetics are limited, hyperporous carbons strike a superior balance between charge storage and ion transport kinetics. However, this advantage is conditional: when micropore connectivity is insufficient or macropore volume is excessive, the expected rate enhancement diminishes, indicating that hierarchical integration must be continuous rather than merely coexisting. Unlike single mesoporous or hollow carbons, hyperporous carbons integrate microporous storage sites with fast ion transport channels, ensuring high volumetric energy density, alongside excellent fast-charging capabilities. Importantly, challenges remain due to the high surface reactivity and mechanical fragility of ultralight porous frameworks, which might lead to reduced initial Coulombic efficiency. In addition, their intrinsically low packing density poses a fundamental limitation for volumetric energy density, which cannot be resolved solely through pore engineering. Future efforts should focus on precise pore engineering, surface chemistry modulation, and integration with conductive nanostructures to unlock the vast potential of hyperporous carbons for next-generation fast-charging energy storage devices.

### 4.2 Heteroatom Doping

While the pore structure of porous carbons is of critical importance for high-performing electrodes, heteroatom doping provides an additional and fundamentally different design lever by modulating both the surface chemistry and the electronic structure of carbon frameworks. Rather than acting as a universally beneficial modification, heteroatom doping introduces a set of competing effects that must be carefully balanced to achieve simultaneous improvements in energy density and power capability. Specifically, doping influences performance through three primary and often interdependent mechanisms: (i) introduction of pseudocapacitive redox-active sites that enhance energy density, (ii) modulation of electronic conductivity and charge-transfer kinetics that governs power performance, and (iii) alteration of surface polarity and pore accessibility that determines ion transport efficiency.^15,41,351,352^

A critical comparison of these effects reveals that not all doping strategies are equally beneficial: while increasing heteroatom content generally improves gravimetric capacitance, excessive functionalization can deteriorate electrical conductivity and block micropores, ultimately limiting rate capability. Therefore, the key design principle is not maximizing dopant concentration, but optimizing the type, configuration, and spatial distribution of heteroatoms to balance energy–power trade-offs. For instance, O is always present to some extent in porous carbon networks, as carbon’s dangling bonds are highly reactive and can bond with atmospheric moisture/oxygen. Oxygen-based functional groups include, but are not limited to, hydroxyl, carbonyl, epoxy, or carboxyl, with the acidic hydroxyl and carboxyl sites providing pseudocapacitance via quasi-reversible redox reactions in the presence of an alkaline electrolyte,^353,354^ and the basic carbonyl/quinone sites participating in electrochemical reactions involving protons in acidic electrolytes.^354^ While oxygen functional groups may improve carbon’s wettability and introduce pseudocapacitance, increasing the oxygen content of carbon has also been related to decreased conductivity and hindered accessibility of micropores by the ions of the electrolyte (Figure 25).^41,351,355^ Thus, oxygen doping is beneficial primarily at moderate levels, where surface wettability is enhanced without compromising electronic transport or pore accessibility.

**Figure 25:**
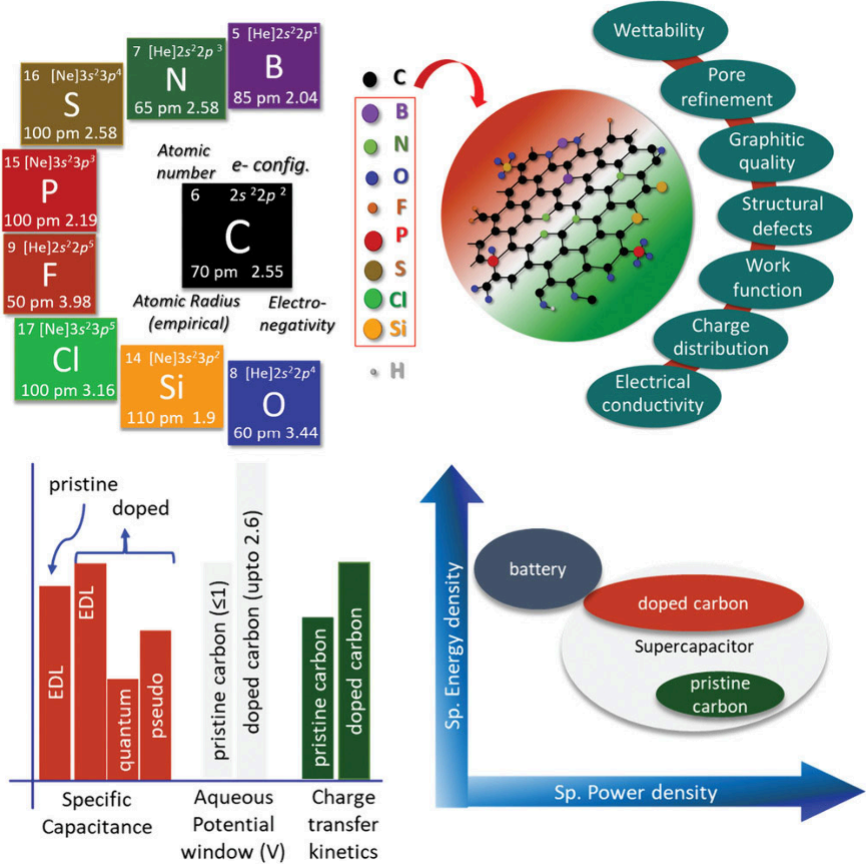
Heteroatom doping (e.g., N, B, P, S, O, and F) can tailor the atomic and electronic structure of carbon, thereby synergistically improving its wettability, pore structure, degree of graphitization, charge distribution, electrical conductivity, and work function. These combined effects enhance quantum capacitance, pseudocapacitive contributions, voltage window, and charge-transfer kinetics, enabling doped carbons to outperform pristine carbons in both energy and power density.^41^ Reproduced with permission from ref 41. Copyright 2020 Wiley.

Among various heteroatoms, the case of nitrogen is by far the most extensively researched owing to the abundance of nature-encountered precursors and simple synthesis, as well as due to its similar atomic size to C (minimizing distortion of carbon structure), and the numerous C–N bonding configurations, each of which endows the carbon network with different attributes. The most frequently encountered nitrogen configurations include graphitic, pyridinic, and pyrrolic N, whereas several other N-based functional groups may be also present in carbon (such as amine, lactam, imide, pyridone, pyridine-N-oxide).^310,356^ Specifically, pyridinic and pyrrolic nitrogen along with quinone oxygen are confirmed to contribute significantly to pseudocapacitance, while quaternary and pyridinic-N-oxide nitrogen enhance electron transfer and capacitance retention, especially at high current loads.^357^ The positively charged graphitic nitrogen contributes a pair of electrons to the conductive π-network improving electron transfer through carbon and increasing its high-rate capability, while the negatively charged pyridinic and pyrrolic configurations do not only improve wettability but also participate in redox reactions providing pseudocapacitance.^358–361^ According to a computational study by Zhan et al., graphitic and pyridinic N doping of graphene can greatly increase its quantum capacitance, proportionally to the doping concentration, whereas pyrrolic N does not provide any enhancement.^362^ This highlights that selective control over nitrogen configuration, rather than total nitrogen content, is essential for optimizing fast-charging behavior.

Similarly to nitrogen, phosphorus behaves as an electron donor and its doping into the carbon structure introduces new states around the Fermi level, enhancing quantum capacitance,^363^ while its various functional groups (e.g., P–C, P–O–H, P–O–C, or P = O) participate in redox reactions providing pseudocapacitance.^356,364–366^ Also, due to the larger atomic size of P compared to C, phosphorus atoms protrude away from the distorted carbon lattice,^367^ deterring agglomeration,^368^ and improving the BET SSA and pore structure of nanocarbons.^369^ Lastly, it has been demonstrated that the C_3_–P=O configuration can effectively stabilize the electrochemical electrode/electrolyte interface, enabling the enlargement of the voltage window in an aqueous electrolyte, improving cycling stability, and decreasing the leakage current.^370^ B-doping introduces holes in the carbon network (acting as an electron acceptor), resulting in higher charge carrier concentration and density of states at the Fermi level. At low B-doping concentration, the chemical adsorption of O is enhanced, promoting the formation of O-based functional groups, which in turn provide pseudocapacitance.^371^ Boron’s functional groups include BC_3_, BC_2_O, and BCO_2_, with BC_3_ and BC_2_O being beneficial for the electric conductivity of the carbon electrode, and BCO_2_ participating in interfacial redox reactions that provide pseudocapacitance.^136,372,373^ S-doping of carbon can occur in various configurations, which include thiophene, thioether, thiol, sulfoxide, sulfone, and sulfonic acid,^356^ with the oxidized sulfur species (e.g., sulfone, sulfoxide, or oxidized thiophene) fortifying the carbon host with pseudocapacitance.^374–377^ Doping with fluorine, the most electronegative element, is known to increase the strain of the carbon host, affecting its SSA and refining its pore structure.^378–380^ Depending on the concentration of fluorine and on its local distribution, the carbon atoms involved the C–F bond may be either sp^3^ (covalent C–F) or sp^2^ (ionic or semi-ionic C–F) hybridized.^381,382^ Due to the high stability of fluorine at the edges of graphitic domains, its beneficial effect in the prevention of carbon corrosion during prolonged cycling has been observed.^379^ While F-doping elevates the hydrophobicity of carbon, potentially diminishing its wettability in aqueous electrolytes, F-doped carbon has been observed to be highly wettable by organic electrolytes and ionogels,^383–385^ while the strong interaction of the C–F bond with metal cations has been leveraged for the improvement of the carbon cathodes of Zn-ion hybrid capacitors.^383,386,387^ This comparison underscores that heteroatoms can be broadly categorized into “energy-enhancing dopants” (e.g., N, P, S) and “conductivity-enhancing dopants” (e.g., graphitic N, B), and their synergistic combination is often required to simultaneously achieve high energy and high power densities.

Moving beyond the conventional role of heteroatom doping in introducing surface functionalities, a deeper impact lies in its ability to engineer the electronic structure of carbon by enriching the Density of States (DoS) near the Fermi level, enhancing quantum capacitance,^41^ and by tuning the work function of carbon materials. As illustrated in Figure 26, the enhancement of energy and power densities in heteroatom-doped carbon electrodes can be rationalized by the interplay between charging potential and adsorption energetics, where optimized minimum adsorption energies maximize energy storage per active site while maintaining high-rate capability. The emerging concept of “noble carbons” demonstrates that carbonization guided by precursor molecular orbital levels can produce carbons with exceptionally high work functions (>6.0 eV) and oxidation resistance.^388^ Conversely, electron-rich precursors yield “reductive carbons” with low work functions (∼2.6 eV). This establishes a continuous electronic spectrum for carbons, enabling precise tuning of the electrode Fermi level.^389^ Such electronic structure control directly optimizes critical supercapacitor interfaces—influencing energy level alignment, voltage window, charge transfer kinetics, and ion adsorption—representing a paradigm shift from chemical doping toward electronic structure design.

**Figure 26:**
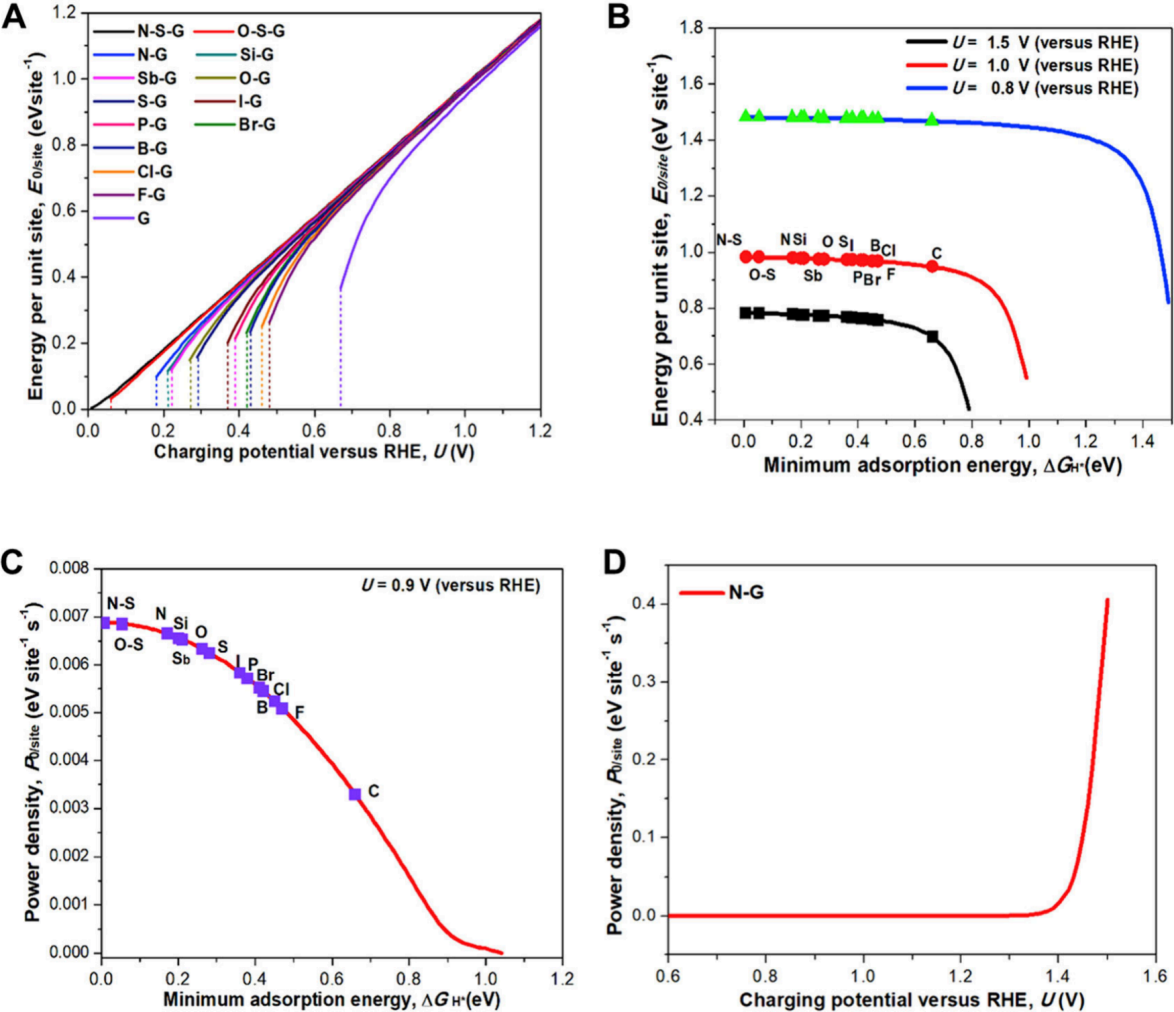
Energy density-enhancement mechanism and design principles for heteroatom-doped carbon-based supercapacitors. Effect of the minimum adsorption energy and the charging potential on energy and power densities. a, energy per unit site versus charging potential U (versus RHE). b, energy per unit area versus minimum hydrogen adsorption energy at a given potential of U = 0.8, 1.0, and 1.5 V. c, power per unit site versus minimum hydrogen adsorption energy at a given potential of U = 0.9 V. d, power per unit site versus charging potential U at minimum hydrogen adsorption energy of 0.17 eV for N-doped graphene.^156^ Reproduced with permission from ref 156. Copyright 2020 Elsevier.

Overall, heteroatom doping should not be viewed as a universally beneficial modification but rather as a multidimensional design variable governed by intrinsic trade-offs. Effective doping strategies for fast-charging energy storage must simultaneously satisfy three key criteria: (i) introducing redox-active sites to enhance energy density while avoiding excessive surface functionalization, (ii) preserving or enhancing electronic conductivity to sustain high rate performance, and (iii) maintaining pore accessibility to ensure efficient ion transport.

Future advances will rely on decoupling these often competing effects through precise control over dopant configuration, spatial distribution, and integration with pore structure engineering, thereby enabling a truly synergistic optimization of energy and power.

In practice, heteroatom-doped porous carbons are most commonly synthesized via two general approaches: direct carbonization of heteroatom-rich precursors, which yields relatively homogeneous doping throughout the carbon framework, and postsynthetic modification of preformed carbons, which primarily introduces dopants at or near the surface.

#### 4.2.1 Monomer Design for Polymer Derived Carbon

Monomer design has emerged as a powerful and versatile strategy for achieving precise structural control in polymer-derived porous carbon (PDCs). By leveraging molecular architecture as a blueprint, this bottom-up approach enables the simultaneous tailoring of pore geometry, size distribution, and specific surface area, thereby directly addressing the structural requirements for high-performance, fast-charging energy storage. As compiled in Table 2, the targeted synthesis of heteroatom-doped porous carbons with superior capacitive performance often involves the pyrolysis of heteroatom-containing polymers, which can be synthesized through various structurally controlled polymerization reactions.

**Table 2:** Comparison of BET Specific Surface Area (SSA_BET_), Elemental Composition, and Specific Capacitance (C_S_) at a Nominal Applied Specific Current (I_D_), of Porous Carbons Originating from Different Monomers^a^.

Monomers	Comment on synthesis	SSA_BET_ (m^2^ g^–1^)	Dopants	C_S_ (F g^–1^) // I_D_ (A g^–1^)	Ref.
P/urea/F	Mg(OH)_2_ template	1504	(*)O, 6.4% N	156 // 10	^390^
P/F/bis-tris/OTAB	KOH activation	1462	9.0% O, 0.7% N	247 // 1	^391^
P/F/phenanthroline	aluminosilicate template	1560	5.5% N	148 // 0.25	^392^
R/F/thiourea	silica template and KOH activation	3599	(*)O, (*)N, (*)S	461.7 // 0.1	^393^
R/F/M/thiourea	HIPE template and KOH activation	1721	8.3% O, 2.9% N, 0.6% S	213.5 // 1	^394^
PG/PEI/F	Mild annealing	221	20.5% O, 28% N	728 // 10	^395^
C/PF/g-C_3_N_4_	Solvent-free	181	3.7% O, 21.7% N	285.2 // 1	^396^
TAPT-THPT-PF	KOH activation	1469	9.2% O, 6.8% N	185 // 1	^397^
P/F/aniline	SBA-15 template	784	5.0% O, 4.7% N	411 // 1	^398^
P/thiourea/F	SBA-15 template	1259	10.7% O, 19.2% N, 1.0% S	511 // 1	
PANI	Silica template	399	10.5%O, 4.3% N	125 // 0.25	^399^
PANI	Palygorskite template	517	(*) O, (*) N	290 // 50	^400^
PANI	Simple pyrolysis	46.4	5.4% O, 7.4% N	163 // 0.1	^401^
PANI-PPy copolymer	KOH activation	3900	(*)O, 4.7% N	937 // 1	^402^
PPy	FeCl_3_-MO template	N.A.	(*) O, (*) N	153.2 // 1	^403^
PANI-PPy	ZnCl_2_ and KOH activation	1080	5.9% O, 6.7% N	550.6 // 1	^404^
PPy	FeCl_3_-MO template	58.9	(*) O, (*) N	228 // 0.9	^405^
DAP/F	Dual-surfactant and CO_2_ activation	1797	2.1% O, 4.6% N, 0.1% S	301 // 0.2	^292^
DAP/F	Simple pyrolysis	778	(*) O, 8.8% N	424 // 1	^406^
M/F	Simple pyrolysis	753	12.2% O, 6.0% N	306 // 0.1	^407^
M/urea/F	KOH activation	2248	7.7% O, 2.2% N	341 // 1	^408^
M/urea/F	SBA-15 template and KOH activation	2553	(*) O, 12.6% N	272 // 10	^409^
M/F/thiourea	Mg(OH)_2_ template	862	9.0% O, 21.8% N, 2.3% S	104 // 1	^410^
M/F/HEDP	Silica template	649	5.1% O, 8.0% N, 0.2% P	132 // 20	^411^
M/PVA/F	Simple pyrolysis	186	10.8% O, 12.4% N	330 // 20	^412^
M/F	Phytic acid and KOH activation	2732	13.1% O, 2.2% N	190 // 10	^413^
M/F	CaCO_3_ template	834	13.4% O, 11.3% N	190 // 20	^414^

a P: phenol, R: resorcinol, PG: phloroglucinol, C: catechol, CNP: cyanophenol F: formaldehyde, PF: paraformaldehyde, M: melamine, DAP: diaminopyridine.

A representative example involves the use of nitrogen-rich monomers such as dopamine, which can be polymerized in the presence of hard templates such as SBA-15 to form ordered mesostructures (Figure 27A). The rigid silica template dictates the pore arrangement, and upon pyrolysis and subsequent template removal, nitrogen-doped ordered nanoporous carbon (NONC) with a well-defined templated mesoporosity is obtained.^415^ These materials exhibit supercapacitor performance approximately twice that of undoped mesoporous carbons, highlighting the critical synergy between a precisely engineered porous architecture and beneficial heteroatom doping for facilitating efficient ion diffusion during electrochemical processes.

**Figure 27:**
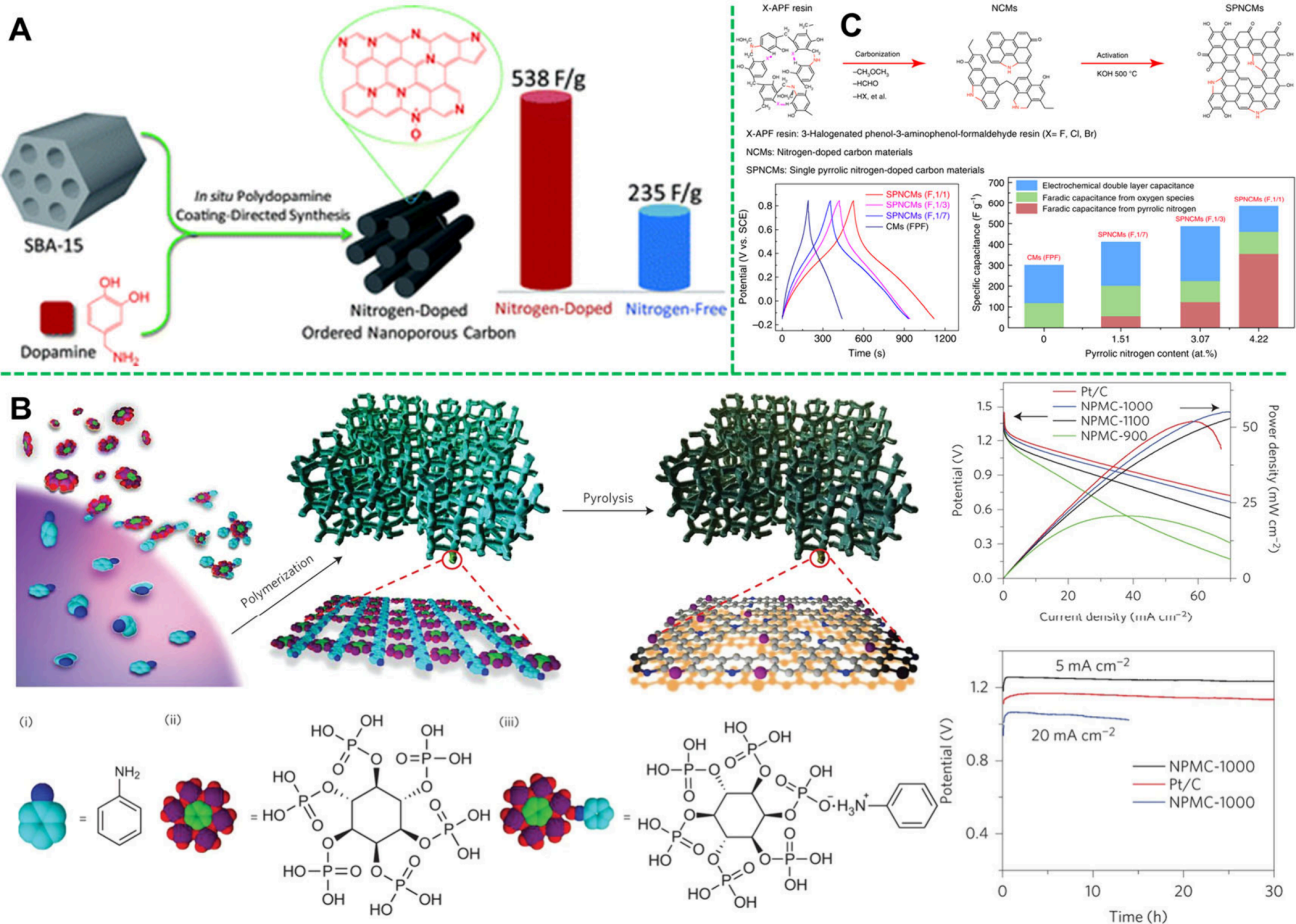
A) Schematic diagram for the route of polydopamine coating-directed synthesis of nitrogen-doped ordered nanoporous carbons.^415^ Reproduced with permission from ref 415. Copyright 2013 Royal Society of Chemistry. B) Schematic illustration of the preparation of N, P codoped porous carbon (NPMC) foams: an aniline–phytic acid complex is formed and polymerized into a 3D PANi hydrogel, which is then freeze-dried and pyrolyzed in Ar to yield NPMC, and their performance of Zn–air battery.^416^ Reproduced with permission from ref 416. Copyright 2015 Springer Nature. C) Single-site pyrrolic-nitrogen-doped sp^2^-hybridized carbon materials and their pseudocapacitance.^417^ Reproduced with permission from ref 417. Copyright 2020 Springer Nature.

Template-assisted polymer-derived strategies for fabricating heteroatom-doped carbons with controlled porosity have been widely investigated. Polymers such as polyaniline (PANI), polypyrrole (PPy), and polyacrylonitrile (PAN) are frequently employed as carbon precursors not only for their high nitrogen content but also for their ability to conform to templated structures. When combined with templating and activation techniques (e.g., ammonia activation), these precursors yield carbons with hierarchically organized porosity and enhanced electrochemical activity. For instance, nitrogen-rich aromatic polymers in combination with colloidal silica templates (Figure 27B), followed by carbonization and ammonia activation, afford carbon materials with replicant porosity from the template, high surface areas, and abundant nitrogen functionalities. These materials demonstrate excellent oxygen reduction reaction (ORR) activity, with a half-wave potential of 0.85 V vs RHE. When used in zinc-air batteries, the resulting metal-free catalysts outperform traditional Pt-based benchmarks.^416^ More importantly, advanced monomer engineering enables the design of specific nitrogen configurations for targeted functionalities.^418^ For example, single pyrrolic nitrogen-doped carbon materials (SPNCMs) have been synthesized via low-temperature dehalogenation and alkaline-activated pyrolysis of 3-halogenated phenol-3-aminophenol-formaldehyde resins (Figure 27C). This strategy allows precise control of pyrrolic nitrogen content (up to 4.22 at.%), significantly enhancing pseudocapacitance and ion transport properties, and confirming pyrrolic nitrogen as a highly active site for electrochemical energy storage.^417^ Furthermore, small organic molecules can also act as efficient precursors. Liang et al. employed o-phenylenediamine as a monomer, together with colloidal silica hard templating and ammonia activation, to synthesize hierarchically porous carbons with optimized nitrogen doping, which exhibited markedly enhanced electrocatalytic performance toward the oxygen reduction reaction and in zinc–air batteries.^419^

Beyond nitrogen, sophisticated monomer engineering approaches have been employed to incorporate other heteroatoms such as phosphorus (P),^420^ boron (B),^421^ fluorine (F),^422^ and sulfur (S),^377^ including multiheteroatom-doped configurations. For example, phosphorus-doped hard carbons synthesized by H_3_PO_4_-assisted cross-linking of epoxy resins followed by pyrolysis exhibit enhanced Li/K-ion storage capacity, rate capability, and cycling stability, attributed to increased active sites and improved structural robustness.^420^ Boron-doped porous carbons, prepared using SBA-15 as a template and inspired by the CMK-3 synthesis route, show surface positive polarization effects, enabling the chemisorption of sulfur and polysulfides. As a result, B-doped carbons stabilize sulfur/carbon cathodes, improve conductivity, and enhance both cycling durability and rate performance.^421^ The introduction of fluorinated functional groups via monomer design has been shown to improve surface polarity and ionic accessibility. For instance, fluorinated covalent triazine frameworks (FCTFs) have been synthesized by self-polymerization of tetrafluoroterephthalonitrile (C_8_F_4_N_2_). The resulting carbons exhibit ultramicroporous structures, high surface areas, and rich heteroatom contents, along with reduced HOMO–LUMO gaps and bandgaps. These materials retain a high capacity of 581 mAh g^–1^ even after 1000 cycles at a high current density of 2 A g^–1^ in alkaline (Na, K) organic batteries.^422^ Furthermore, sulfur and nitrogen codoped mesoporous hollow carbon spheres have been synthesized using CaCO_3_ templates, delivering a capacity of 144 mAh g^–1^ at 20 A g^–1^ and excellent cycling stability for fast sodium storage. The synergistic effects of S/N codoping and hollow mesoporous architectures significantly enhance ion diffusion and capacitive energy storage.^150^ In addition, a molecular cross-linking strategy has also been developed to create nitrogen and phosphorus codoped mesoporous soft carbons (NPSC) by reacting polyphosphoric acid with p-phenylenediamine, followed by in situ activation during pyrolysis. These materials feature enlarged interlayer spacings, shortened ion diffusion paths, and abundant active sites, achieving a high-rate capacity of 215 mAh g^–1^ at 10 A g^–1^ and excellent durability over 4700 cycles.^423^

Thus, monomer engineering offers a bottom-up, multifunctional, and effective route to fine-tuning the doping chemistry, porosity, and electronic structure of polymer-derived porous carbons. This approach lays a robust foundation for the rational design of next-generation materials for fast-charging energy-storage systems.

#### 4.2.2 Ionic Liquid Derived Carbon

Ionic liquids (ILs) have been recognized as versatile carbon precursors for synthesizing heteroatom-doped porous carbons due to their unique physicochemical properties, including high thermal stability, intrinsic ionic structure, and designable functional groups. The synthesis of ionic liquid-derived carbons (ILCs) typically involves direct pyrolysis of neat ILs under inert atmospheres ranging from 500 to 1000 °C. The final carbon structure, heteroatom doping level (e.g., nitrogen or sulfur), and overall porosity are significantly influenced by the chemical nature of the IL, particularly the identity of the cation (e.g., imidazolium, pyridinium, phosphonium) and anion (e.g., BF_4_^–^, PF_6_^–^, NTf_2_^–^). In 2009, Dai and co-workers first demonstrated the feasibility of converting ILs into nitrogen-doped porous carbons using task-specific ionic liquids (TSILs) containing one or two imidazolium units and reactive nitrile groups, resulting in hierarchically micro/mesoporous N-doped carbon networks (Figure 28A).^424^ In these systems, bulky anions such as bis­(trifluoromethanesulfonyl)­imide ([NTf_2_]^−^) played a critical role in generating porosity by serving as a self-template during carbonization. TSILs incorporating cross-linkable cations or anions can also yield nanoporous carbons enriched in nitrogen or boron, with the pore size largely determined by the decomposition of the noncross-linkable counterions. To achieve high surface areas and pore volumes suitable for electrochemical applications, larger anions are generally preferred. Alternatively, TSILs with larger cations containing two nitrile moieties provided a route to carbons with tunable microporous and mesoporous structures at high carbonization yields, enhancing their charge storage capacities (Figure 28B).^425^ By adjusting the carbonization temperature and the ratio of ionic liquids (ILs) containing cross-linkable anions, such as 1-butyl-3-methylimidazolium tricyanomethanide [BMIm]­[C­(CN)_3_] and 1-ethyl-3-methylimidazolium tetracyanoborate [EMIm]­[B­(CN)_4_], boron- and nitrogen-rich carbons featuring slit-shaped pores and a specific surface area exceeding 500 m^2^ g^–1^ were synthesized (Figure 28C).^426^ Furthermore, it was found that the addition of FeCl_2_ during the IL carbonization process not only promotes the graphitization of carbon layers but also effectively regulates the formation of mesoporous structures, achieving excellent specific surface area and high conductivity at 900 °C (Figure 28D).^427^

**Figure 28:**
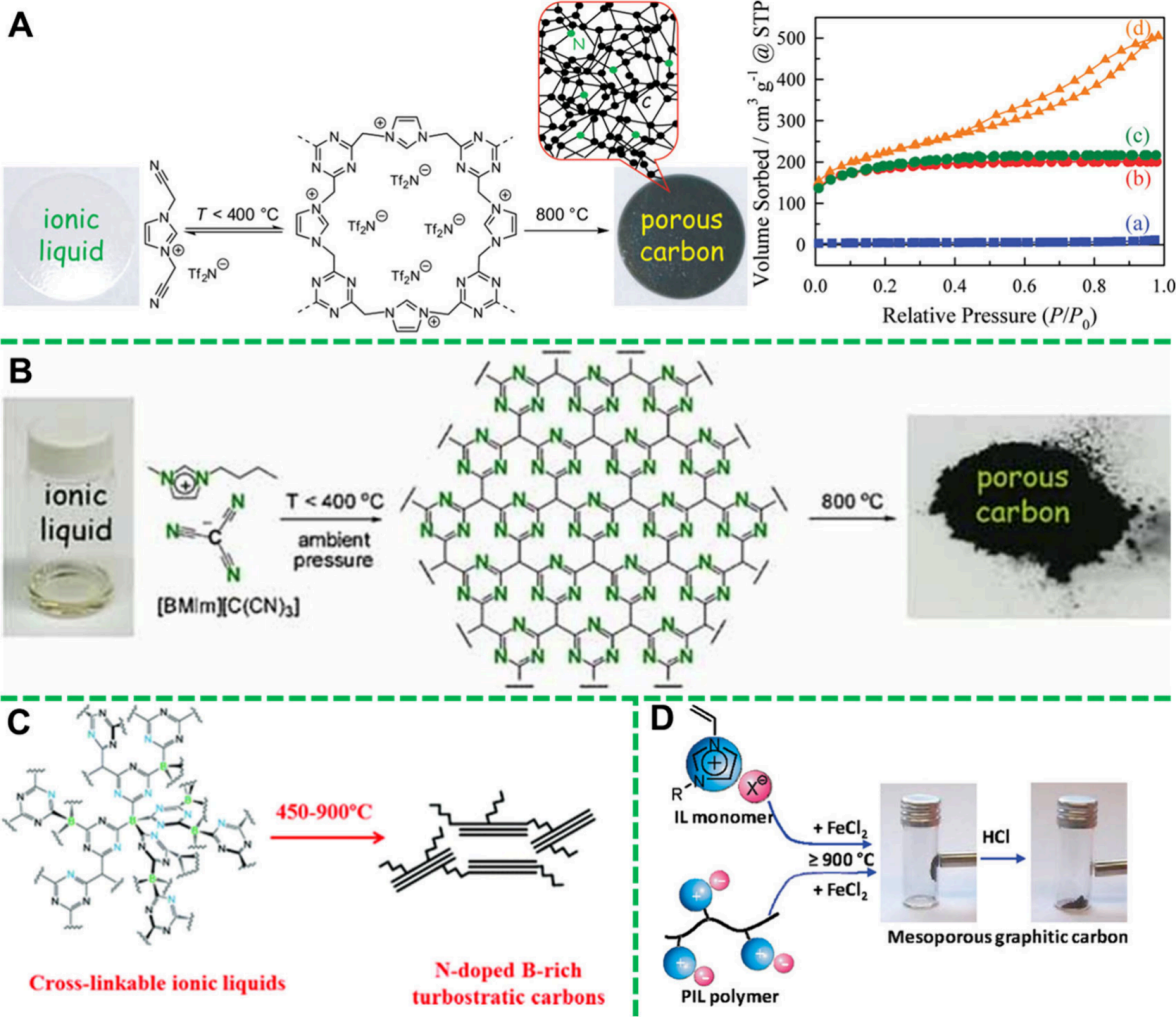
A) Facile ionothermal synthesis of microporous and mesoporous carbons from task specific ionic liquids.^424^ Reproduced with permission from ref 424. Copyright 2009 American Chemical Society. B) Schematic illustration of the direct pyrolysis of task-specific ionic liquids into N-rich porous carbons.^425^ Reproduced with permission from ref 425. Copyright 2010 Wiley. C) Boron and nitrogen-rich carbons from ionic liquid precursors with tailorable surface properties.^426^ Reproduced with permission from ref 426. Copyright 2011 Royal Society of Chemistry. D) Schematic illustration of the template-free synthesis of mesoporous graphitic carbons from ionic liquid precursors with FeCl_2_-assisted carbonization.^427^ Reproduced with permission from ref 427. Copyright 2010 American Chemical Society.

Beyond this intrinsic templating effect, ILs can also be employed in conjunction with sacrificial hard or soft templates—such as silica, ZnO, or block copolymers—to engineer specific pore structures. Subsequent template removal allows for the creation of microporous, mesoporous, or hierarchically porous carbon frameworks. Dai et al. demonstrates the confined pyrolysis of ionic liquids within a silica network by preparing IL-containing silica gels via sol–gel processing, followed by carbonization and silica removal to yield porous carbon materials with tunable IL content (Figure 29A).^428^ Structurally, ILCs offer high tunability in pore size distribution, surface functionality, and heteroatom content, all of which are critical for fast ion transport and charge storage in high-rate electrochemical energy storage systems. For example, nitrogen and oxygen codoped carbon nanobubbles with thin graphitic shells and hierarchical porosity have been synthesized via infiltration of ILs into silica templates followed by annealing (Figure 29B). These nanostructures exhibit high specific capacity, excellent rate capability, and improved long-term cycling stability when employed as anodes in lithium- and sodium-ion batteries. The superior performance is attributed to enhanced ion transport through multiscale pores and interfacial capacitance from heteroatom-enriched surfaces.^429^ Salt-templating approaches have also been developed, wherein ILs are mixed with eutectic salts (e.g., ZnCl_2_/KCl) and pyrolyzed to form vertically aligned (Figure 29C), nitrogen-doped graphitic carbon nanosheets with high surface area and interconnected ion transport channels, delivering promising performance for fast-charging applications.^430^

**Figure 29:**
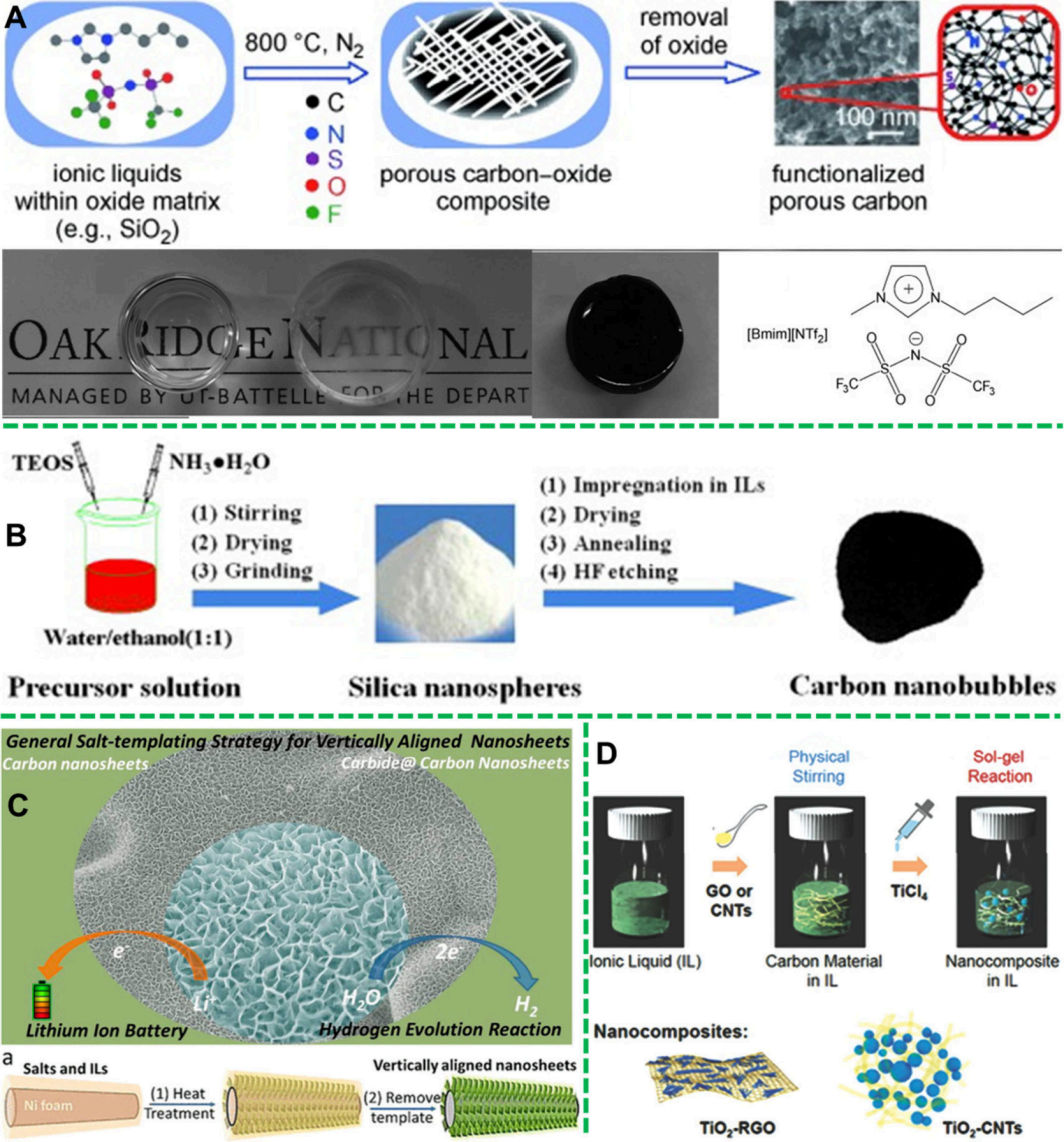
A) Schematic illustration of confinement carbonization: IL thermolysis in oxide frameworks yields heteroatom-doped porous carbons and carbon–oxide composites.^428^ Reproduced with permission from ref 428. Copyright 2010 Wiley. B) Schematic illustration of the synthesis of N, O codoped carbon nanobubbles via silica-templated IL impregnation and annealing.^429^ Reproduced with permission from ref 429. Copyright 2014 Elsevier. C) Schematic illustration of the salt-templating process for the formation of vertically aligned two-dimensional (2D) nanosheets.^430^ Reproduced with permission from ref 430. Copyright 2015 American Chemical Society. D) Ionic liquid-assisted synthesis of TiO_2_–carbon hybrid nanostructures.^431^ Reproduced with permission from ref 431. Copyright 2016 Wiley.

The ability to control the precursor composition at the molecular level enables precise tuning of the resulting carbon’s structural and electronic properties. Nonetheless, challenges remain regarding yield, cost, and scalability, as many ILs are expensive and difficult to recover postsynthesis. Moreover, the complex thermal decomposition pathways of ILs can result in variable carbon morphologies without carefully optimized conditions. To address these challenges, researchers have explored the rational design of task-specific ionic liquids (TSILs) incorporating preinstalled porogens or volatile fragments to promote pore formation during pyrolysis. Co-pyrolysis of ILs with additional carbon sources—such as GO, CNTs, glucose, cellulose, or other biomass-derived precursors—has also been employed to improve carbon yield, reduce production cost, and tailor structural properties (Figure 29D).^431^ Alternatively, ILs can be combined with metal or metal salt precursors to generate oxide/carbon or metal/carbon composite materials upon pyrolysis, offering a versatile route toward structurally integrated and functionally enhanced hybrid carbon frameworks.^158,432^

Overall, ILCs represent a promising and rapidly evolving subclass of SPCs. Their combination of tunable porosity, high surface area, and intrinsic heteroatom doping makes them particularly attractive for energy storage devices, especially in supercapacitors and fast-charging batteries, where rapid ion diffusion and high conductivity are essential.

#### 4.2.3 Treatment for Doping

Beyond molecular precursor selection, a range of chemical and physical treatments—applied either during or after carbonization—have been developed to facilitate heteroatom doping and simultaneously regulate the pore structure of synthetic carbons. Among these, molten salt-mediated synthesis has emerged as a powerful in situ strategy.^436,437^ In this approach, salts such as ZnCl_2_, KCl, or NaCl serve dual functions: they act as porogens or structure-directing agents and provide a confined environment for the carbonization of heteroatom-containing polymers or additives. This method enables uniform heteroatom incorporation (e.g., N, S, or P) under high-temperature molten conditions while promoting the development of hierarchically porous structures with partially graphitized domains. The resulting carbons typically exhibit high heteroatom content, tunable micro/mesoporosity, and enhanced conductivity, making them highly suitable for high-rate charge storage. For instance, Cai et al. synthesized aerogel-like nitrogen-doped carbons (NDC-MS) by carbonizing glycine in a NaCl/ZnCl_2_ eutectic molten salt system (Figure 30A), achieving an ultrahigh specific surface area (1548.6 m^2^ g^–1^) and well-developed hierarchical porosity, which resulted in outstanding oxygen reduction reaction (ORR) performance.^433^ Similarly, He et al. prepared high nitrogen-content carbon nanosheets by employing a Schiff-base reaction between melamine and terephthalaldehyde in a LiCl-KCl molten salt medium (Figure 30B), obtaining materials with a specific surface area of up to 696 m^2^ g^–1^, a high nitrogen content of ∼30 wt% predominantly in pyrrolic and pyridinic forms, and a unique two-dimensional structure, which significantly improved the capacity and rate performance as lithium-ion battery anodes.^434^ Notably, Weng et al. demonstrated an innovative approach where CO_2_ was electrochemically captured and converted into carbon nanotube structures during molten salt electrolysis (NaCl-CaCl_2_-CaO system), achieving simultaneous CO_2_ fixation and carbon structure formation (Figure 30C). This strategy not only enables in situ doping but also significantly reduces carbon emissions during materials synthesis.^435^ However, postsynthetic washing is required to remove residual salts, and scalability can be limited by salt recovery and corrosion concerns.

**Figure 30:**
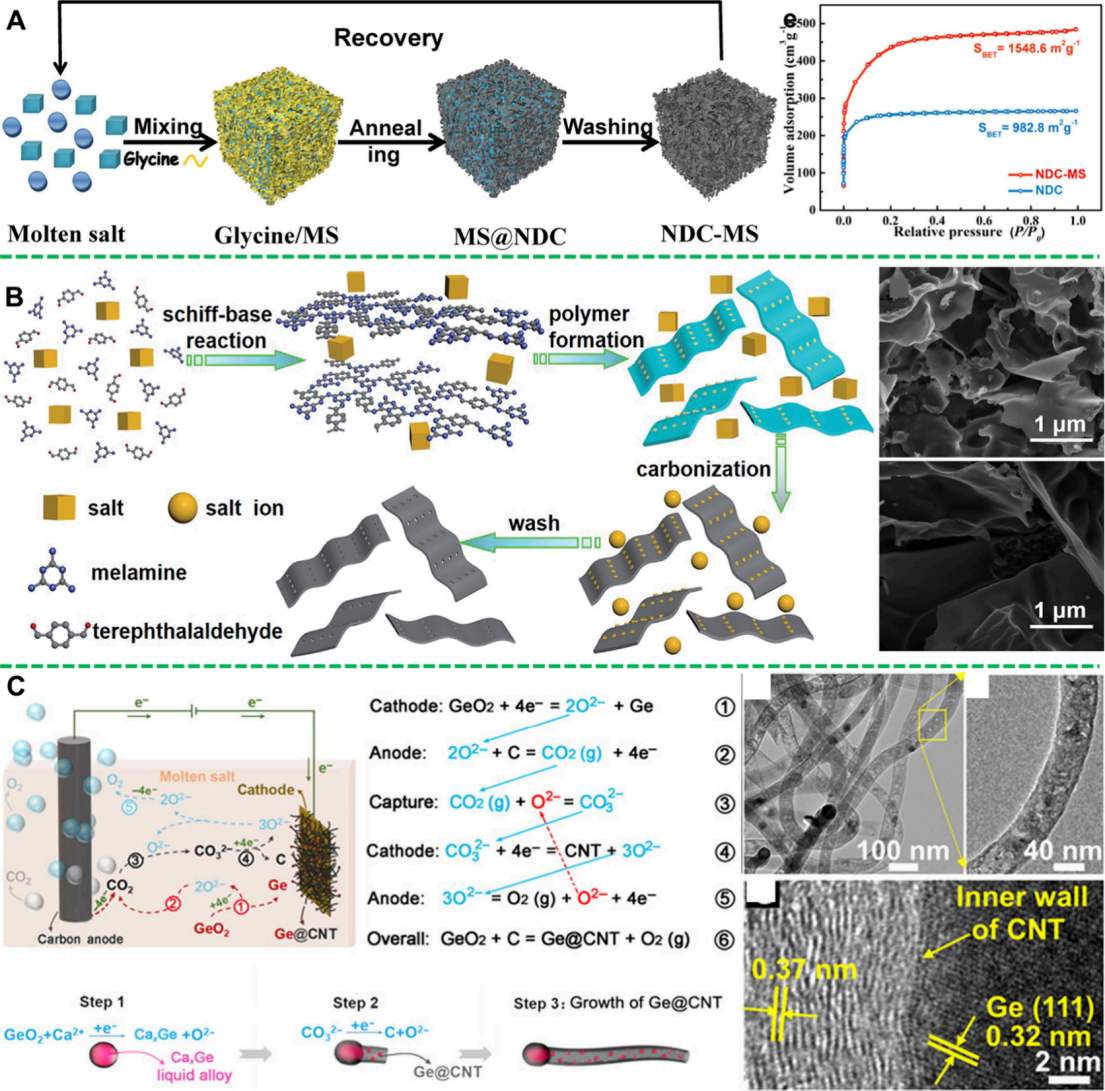
A) Schematic illustration for the synthesis of nitrogen doped carbon (NDC-MS) via molten salt method.^433^ Reproduced with permission from ref 433. Copyright 2020 Elsevier. B) High nitrogen-content carbon nanosheets formed using the Schiff-base reaction in a molten salt medium as efficient anode materials for lithium-ion batteries.^434^ Reproduced with permission from ref 434. Copyright 2015 Royal Society of Chemistry. C) In situ electrochemical conversion of CO_2_ in molten salts to advanced energy materials.^435^ Reproduced with permission from ref 435. Copyright 2020 American Association for the Advancement of Science.

Thermal activation and gas-phase treatments represent another category of doping-related processes, frequently applied as post-treatments. Exposure of carbonized frameworks to reactive gases such as NH_3_, CO_2_, or H_2_O vapor at elevated temperatures can introduce heteroatoms while tailoring surface functionalities and porosity.^438^ For example, NH_3_ treatment not only dopes nitrogen into graphitic frameworks but also generates abundant surface defects, which act as active sites for ion adsorption.^438–440^ Similarly, CO_2_ and steam activation selectively etch disordered carbon regions, creating a high density of micropores that facilitate ion transport. Hydrothermal oxidation or steam activation with H_2_O can introduce oxygen-containing functional groups such as hydroxyl (−OH) and carboxyl (−COOH) onto the carbon surface, thereby enhancing the hydrophilicity and ion adsorption capability of the material.^144,441,442^ These gas treatments are particularly valuable when the original polymer precursors lack sufficient heteroatom content or when precise surface modification is desired. Nevertheless, the high temperatures required for effective doping may degrade pore integrity or reduce conductivity if not carefully controlled.

To address these limitations, activation-assisted strategies have been developed that enable simultaneous heteroatom incorporation and pore structure evolution under comparatively milder conditions. For example, He et al. synthesized three-dimensional N,O-codoped egg-box-like carbons using coal tar pitch with K_2_CO_3_ as a pore-forming agent and carbon cloth as a template. In this system, heteroatom incorporation occurs concurrently with carbonization and activation, avoiding the need for additional postfunctionalization or harsh chemical treatments.^443^ Notably, the resulting material can be purified by simple water washing, representing a more environmentally benign alternative to conventional acid-assisted activation processes. The hierarchical architecture, combined with N,O functionalities, enables efficient ion transport and enhanced surface wettability, leading to excellent cycling stability (1.9% capacitance decay after 50,000 cycles) and a high areal capacitance of 27.6 μF cm^–2^. Despite these advantages, as with many activation-driven approaches, the spatial distribution and chemical configuration of heteroatoms remain difficult to precisely control, making it challenging to decouple the respective contributions of doping and pore architecture.

Complementary to these activation and gas-phase treatments, acid washing and template removal processes also play critical roles in exposing active sites and eliminating inactive residues that may impede electrochemical performance. Silica or MgO templates, used to generate ordered meso/macropores, must be removed using strong acids (e.g., HF or HCl), which can also alter surface chemistry.^444,445^ Together, these treatment strategies offer a modular toolbox for heteroatom doping and structural engineering of SPCs. While each approach presents unique advantages—such as high dopant uniformity, hierarchical porosity, or enhanced defect density—they also come with trade-offs including processing complexity, environmental impact, or structural degradation. A synergistic combination of controlled doping and structural modulation remains key to achieving the fast ion transport and surface reactivity required for high-performance energy storage applications.

#### 4.2.4 Challenges and Future Directions in Heteroatom Doping

Heteroatom doping in synthetic porous carbons remains a powerful yet underoptimized strategy for balancing energy density and rate capability. While significant progress has been made in introducing pseudocapacitive functionalities and tuning the electronic structure, a fundamental challenge lies in decoupling beneficial surface redox activity from the accompanying kinetic penalties and stability issues, particularly under high-voltage or high-rate conditions.

A key open question is how to achieve spatially and chemically controlled doping within the hierarchical pore networks. In most current systems, heteroatoms are randomly distributed, making it difficult to distinguish their roles in micropores, mesopores, and external surfaces. Future efforts should therefore focus on site-selective doping strategies, where heteroatom functionalities are deliberately positioned to maximize interfacial charge transfer while minimizing the transport resistance.

In addition, the interaction between heteroatom functionalities and electrolyte systems remains insufficiently understood. Emerging studies suggest that doping not only introduces redox-active sites but also modulates the electric double-layer structure, ion desolvation behavior, and ion affinity, particularly in nonaqueous electrolytes and ionic-liquid-based systems. This highlights the need for a more integrated design paradigm that couples surface chemistry engineering with electrolyte optimization rather than treating them as independent variables.

Looking ahead, advancing this field will require the integration of operando characterization techniques and data-driven approaches to quantitatively resolve how heteroatom type, configuration, and spatial distribution influence charge storage mechanisms across multiple length scales. Establishing such predictive relationships will be essential for transforming heteroatom doping from an empirical enhancement strategy into a rational design tool for achieving simultaneous high energy density, high power capability, and long-term stability in next-generation supercapacitors.

### 4.3 Synthetic Carbonaceous Networks

While polymer-derived synthetic carbons typically require carbonization at temperatures above 600 °C, synthetic carbonaceous networks provide an alternative route to construct porous carbon frameworks under significantly milder conditions, in some cases even at ambient temperature. Rather than representing merely new synthetic routes, these approaches fundamentally differ in how they control carbon network formation—either through interfacial reactions, mechanically driven bond reconstruction, or bottom-up π-conjugated polymerization. This distinction is critical, as each strategy governs a different set of structure-determining parameters, including graphitic ordering, pore connectivity, and heteroatom distribution, which in turn dictate the balance between energy density and rate capability.

From a functional perspective, synthetic carbonaceous networks can be broadly categorized into three design paradigms: (i) interfacial or alloy-mediated reconstruction that promotes graphitic ordering and conductivity, (ii) mechanochemical pathways that enable rapid formation of disordered yet porous sp^2^ networks, and (iii) π-conjugated frameworks that translate molecular-level design into well-defined conductive architectures. These paradigms differ not only in synthesis conditions, but more importantly in their ability to decouple ion transport and charge storage, a key requirement for fast-charging systems.

#### 4.3.1 Liquid Alloy Catalytic Polymerization

Liquid alloy catalytic polymerization has recently emerged as a promising approach to constructing synthetic carbonaceous networks with tailored porosities and superior electrochemical performance. This strategy leverages the unique properties of liquid metal alloys to catalyze the formation of highly interconnected carbon frameworks under mild conditions.

More importantly, the defining feature of this approach lies in its interfacial reaction environment, which simultaneously governs carbon nucleation, growth, and templating. This enables partial graphitic ordering and continuous network formation even at relatively low temperatures, distinguishing it from conventional polymer-derived carbons. As a result, liquid alloy-mediated synthesis primarily targets the improvement of electronic conductivity and structural connectivity—two key parameters for high-rate performance.

A notable advancement in this area is the liquid sodium-potassium (Na/K) alloy interfacial engineering strategy reported by Tang et al. Using CCl_4_ as the carbon precursor and Na/K alloy as the reducing agent (Figure 31A), porous carbon frameworks were synthesized at ambient temperature without the need for high-temperature pyrolysis. In this process, in situ-generated NaCl/KCl salts acted as sacrificial templates, resulting in a hierarchical porous structure. The porous carbons retained abundant carbon-chlorine (C–Cl) bonds, which enabled facile postsynthetic functionalization, leading to materials with excellent catalytic activity and structural stability. This approach offers a novel route to customizing surface chemistry while maintaining high porosity, beneficial for fast ion transport and efficient charge storage.^446^

**Figure 31:**
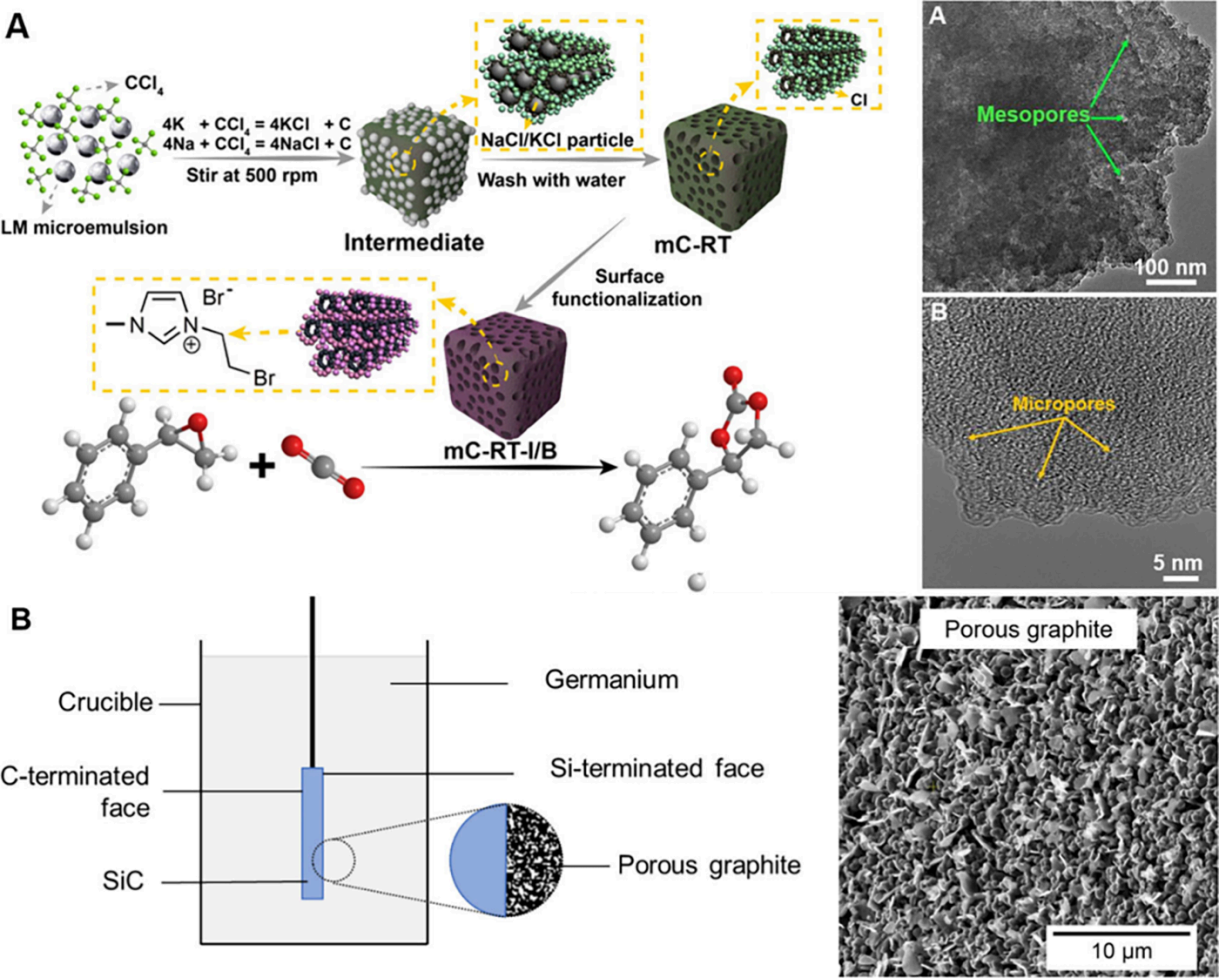
A) Synthetic route of functional porous carbon via liquid metal interfacial engineering strategy.^446^ Reproduced with permission from ref 446. Copyright 2022 Wiley. B) Schematic diagram depicting dealloying process of single crystal SiC in molten Ge, Ge dissolves Si from the parent alloy, leaving behind a porous graphite network.^447^ Reproduced with permission from ref 447. Copyright 2020 Elsevier.

In addition to polymer-derived approaches, liquid metal dealloying (LMD) has been extended to ceramic precursors for the fabrication of synthetic carbonaceous frameworks. Greenidge et al. demonstrated the successful synthesis of porous graphite by immersing silicon carbide (SiC) samples into molten germanium (Ge) at 1400 °C, followed by nitric acid etching to remove the solid Ge phase (Figure 31B). The resulting porous graphite exhibited a three-dimensional interconnected structure with controllable ligament size, low defect density, and high crystallinity, as evidenced by an I­(D)/I­(G) ratio of 0.3 for samples processed at the highest temperatures. Kinetic studies revealed that the dealloying depth followed a square-root-of-time dependence, and the high activation barrier suggested a Si diffusion mechanism impeded by unsaturated carbon bonds near the interface.^447^ In contrast to low-temperature interfacial polymerization, liquid metal dealloying (LMD) shifts the design priority toward maximizing graphitic ordering and electrical conductivity. The resulting frameworks exhibit superior electron transport and structural robustness, which are advantageous for thick electrodes and extreme fast-charging conditions. However, this improvement in conductivity is inherently achieved at the expense of microporosity and surface functionality, thereby limiting charge storage sites and constraining energy density.

More broadly, liquid alloy-based strategies—whether based on interfacial polymerization or dealloying—share a common structural outcome: the formation of continuous, well-connected carbon backbones that favor efficient electron transport. This makes them intrinsically suited for enhancing power capability. However, because these approaches do not inherently promote micropore-dominated charge storage, their contribution to energy density remains limited.

This dichotomy highlights a fundamental limitation: liquid-alloy-mediated synthesis is effective for improving rate performance but cannot, on its own, deliver a balanced optimization of energy and power. Achieving such a synergy therefore requires coupling with complementary strategies that explicitly introduce ion-accessible microporosity or redox-active functionalities.

#### 4.3.2 Mechanochemistry

Mechanochemistry has emerged as an efficient and sustainable strategy for the construction of synthetic carbonaceous networks. Unlike conventional solution-based or ionothermal methods, mechanochemical synthesis operates under solvent-free, ambient conditions, providing distinct reaction environments that can facilitate bond formation, enhance polymerization kinetics, and broaden the scope of accessible materials. Mechanochemical processes rely on mechanical forces, typically applied via ball milling, to promote chemical transformations through localized high-energy impacts. These impacts induce bond cleavage and subsequent recombination, enabling the synthesis of various conjugated scaffolds with highly extended π-systems and porous structures. More fundamentally, the defining feature of mechanochemistry lies in its nonequilibrium reaction pathway, where rapid bond cleavage and reconstruction favor the formation of defect-rich, highly interconnected sp^2^-carbon networks with hierarchical porosity. This structural motif inherently enhances ion accessibility and shortens diffusion pathways, making mechanochemical carbons particularly advantageous for improving charge storage kinetics and thus energy delivery at practical rates. Importantly, mechanochemistry circumvents the use of hazardous organic solvents, shortens reaction times, and allows the use of monomers with limited solubility—advantages that are particularly valuable for fabricating porous carbonaceous frameworks for energy storage applications.^451–455^

Dai’s group has contributed a series of works to this field.^452,456^ In 2017, they reported an innovative approach to prepare ordered mesoporous carbon via the ball milling of block copolymer F127 (Figure 32A), enabling a solid-state coordination-driven self-assembly.^448^ Building on this, they later developed a mechanochemical graphitization strategy for producing crystalline carbon frameworks. Notably, by ball milling graphite-phase carbon nitride (g-C_3_N_4_) with magnesium at ambient temperature led to the denitridation and rearrangement into highly crystalline graphite without the need for high-temperature treatment (Figure 32B), thus offering a low-energy route to conductive sp^2^-carbon frameworks.^449^ In parallel, Casco and co-workers demonstrated that ball milling hexachlorobenzene (C_6_Cl_6_) with calcium carbide (CaC_2_) leads to the formation of porous carbon with a highly ordered sp^2^ microstructure (Figure 32C).^450^ Extending this approach, they employed cyanuric chloride (C_3_Cl_3_N_3_) and CaC_2_ to produce nitrogen-doped porous carbon at room temperature.^457^

**Figure 32:**
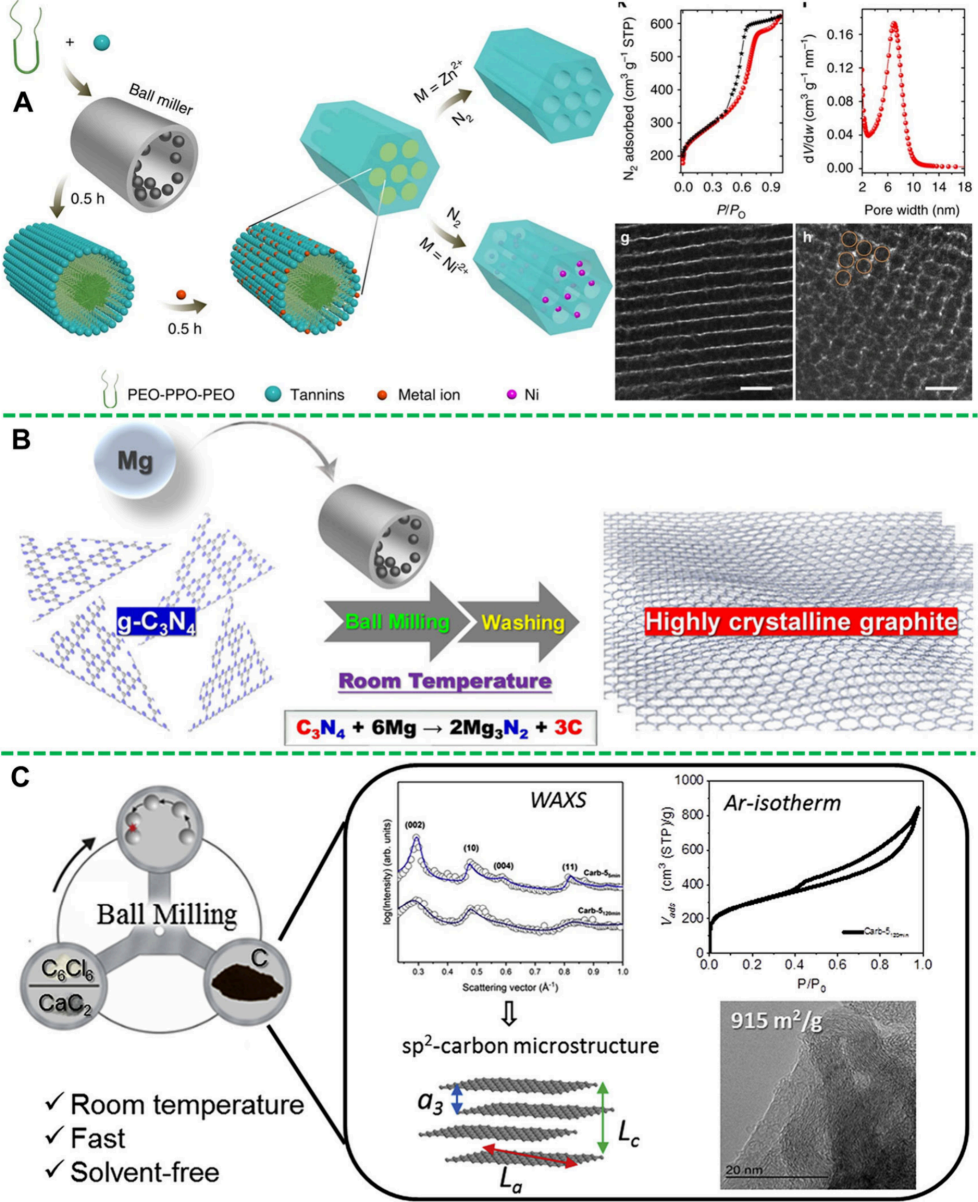
A) Solid-state synthesis of ordered mesoporous carbon catalysts via a mechanochemical assembly through coordination cross-linking.^448^ Reproduced with permission from ref 448. Copyright 2017 Springer Nature. B) Mechanochemical synthetic approach to afford highly crystalline graphite.^449^ Reproduced with permission from ref 449. Copyright 2020 Wiley. C) Mechanochemical synthesis of porous carbon at room temperature with a highly ordered sp^2^ microstructure.^450^ Reproduced with permission from ref 450. Copyright 2018 Elsevier.

Beyond the synthesis of graphitic and sp^2^-carbon frameworks, mechanochemistry has enabled the polymerization of aromatic monomers into extended conjugated networks.

The mechanochemical construction of conjugated porous networks (CPNs) has been widely demonstrated using electron-rich building blocks, such as carbazole, benzene, and naphthalene derivatives. In oxidative polymerization systems promoted by Lewis acids (e.g., FeCl_3_), ball milling enabled the rapid synthesis of porous CPNs with surface areas up to 1850 m^2^ g^–1^ and hierarchical pore structures.^458^ Beyond simple oxidation coupling, Friedel–Crafts alkylation reactions driven by mechanochemistry were also employed to fabricate covalent triazine frameworks (CTFs) with tunable porosity and nitrogen content (Figure 33A). These frameworks exhibit excellent potential as electrode materials due to their high surface areas, abundant redox-active sites, and robust conjugated backbones.^459^

**Figure 33:**
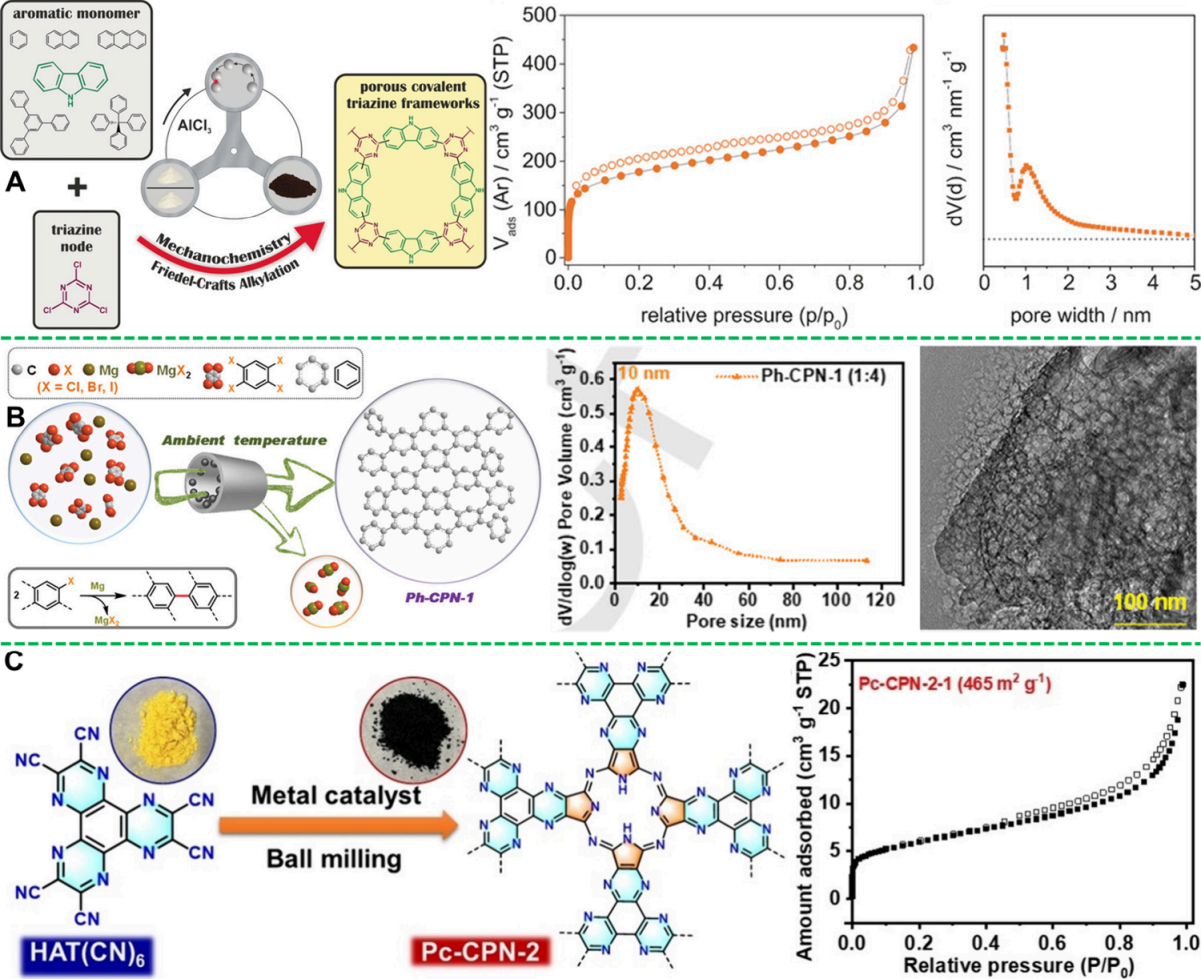
A) Ball milling was applied to synthesize covalent triazine frameworks with permanent porosity.^459^ Reproduced with permission from ref 459. Copyright 2017 Wiley. B) Benzene ring knitting achieved by ambient-temperature dehalogenation via mechanochemical Ullmann-type reductive coupling.^460^ Reproduced with permission from ref 460. Copyright 2021 Wiley. C) Fully conjugated poly­(phthalocyanine) scaffolds derived from a mechanochemical approach.^461^ Reproduced with permission from ref 461. Copyright 2022 Wiley.

Expanding beyond electron-rich systems, mechanochemical strategies have also been adapted for electron-deficient monomers, using reductive coupling reactions catalyzed by metal powders (e.g., Mg, Al, Zn). For instance, Mg-promoted homocoupling of tetrabromobenzene yielded amorphous CPNs featuring hierarchical porosity, and the approach was further extended to polymerize challenging monomers such as hexabromotriphenylene (Figure 33B). These results highlight mechanochemistry’s ability to access carbonaceous frameworks that are otherwise difficult to obtain by solution-based routes.^460^

Moreover, mechanochemistry has enabled the synthesis of poly­(phthalocyanine) frameworks by reductive polymerization of aromatic nitriles. Using metal catalysts such as Mg, aromatic nitrile monomers like 1,2,4,5-tetracyanobenzene (TCB) were converted into conjugated macrocyclic networks featuring nitrogen-doped structures (Figure 33C), high surface areas (up to 357 m^2^ g^–1^), and enhanced electrochemical properties. The choice of metal catalysts critically influenced the polymerization efficiency and the resulting material’s porosity and heteroatom incorporation.^461^

From a structure–performance standpoint, mechanochemical approaches are particularly effective in maximizing surface accessibility, defect density, and heteroatom incorporation, all of which contribute to enhanced charge storage capacity and pseudocapacitive behavior. However, these advantages are often accompanied by limited long-range graphitic ordering and moderate electrical conductivity, which can become a bottleneck under ultrahigh-rate conditions.

Therefore, while mechanochemistry is highly effective for improving energy density and ion transport kinetics, it is intrinsically less suited for maximizing electron transport compared with highly graphitized systems. This highlights a fundamental tradeoff: the same structural disorder that enhances ion accessibility may compromise the electronic conductivity.

#### 4.3.3 π-Conjugated Carbon Networks

π-Conjugated carbon networks represent a unique class of SPCs characterized by extended π-electron delocalization along their polymeric backbones. These networks are typically constructed through bottom-up polymerization strategies from sp^2^- or sp-hybridized monomers bearing aromatic rings, ethynyl groups, or vinylene linkers, leading to conjugated microporous polymers (CMPs), π-conjugated covalent organic frameworks (π-COFs), and graphdiyne (GDY) and its derivatives. Distinct from both liquid alloy and mechanochemical strategies, the defining feature of π-conjugated networks lies in their ability to couple electronic structure and pore architecture at the molecular level. This enables simultaneous control over charge transport pathways and ion-accessible porosity, positioning them as one of the few material platforms capable of addressing both energy density and power capability within a unified design framework.

CMPs are commonly synthesized via transition-metal-catalyzed carbon–carbon coupling reactions, such as Sonogashira–Hagihara, Suzuki, or oxidative acetylene coupling, resulting in amorphous yet fully conjugated three-dimensional networks.^465^ For instance, HATN-CMP, synthesized via Sonogashira cross-coupling, exhibits a hierarchical porous structure with a high surface area (616 m^2^ g^–1^), enabling efficient lithium-ion transport and stable battery performance with a near-unity Coulombic efficiency and high-capacity retention over 50 cycles.^466^ In contrast, π-COFs are constructed via dynamic covalent reactions—including Knoevenagel condensation and β-ketoenamine formation—to yield crystalline two- or three-dimensional skeletons with long-range π-conjugation. Notably, conjugated 3D COFs such as 3D-scu-COF-1 and 3D-scu-COF-2 (Figure 34A), built from a 3D homoaromatic conjugated pentipyrene-based building block and 2D fully conjugated linkers, exhibit excellent electrical conductivity (3.2–3.5 × 10^–5^ S cm^–1^), high surface areas (up to 2340 m^2^ g^–1^), and interconnected pore structures. These features make them promising sulfur host materials for lithium–sulfur batteries, delivering high capacities (1035–1155 mA h g^–1^ at 0.2 C) and superior cycling stability (71–83% capacity retention after 500 cycles).^462^

**Figure 34:**
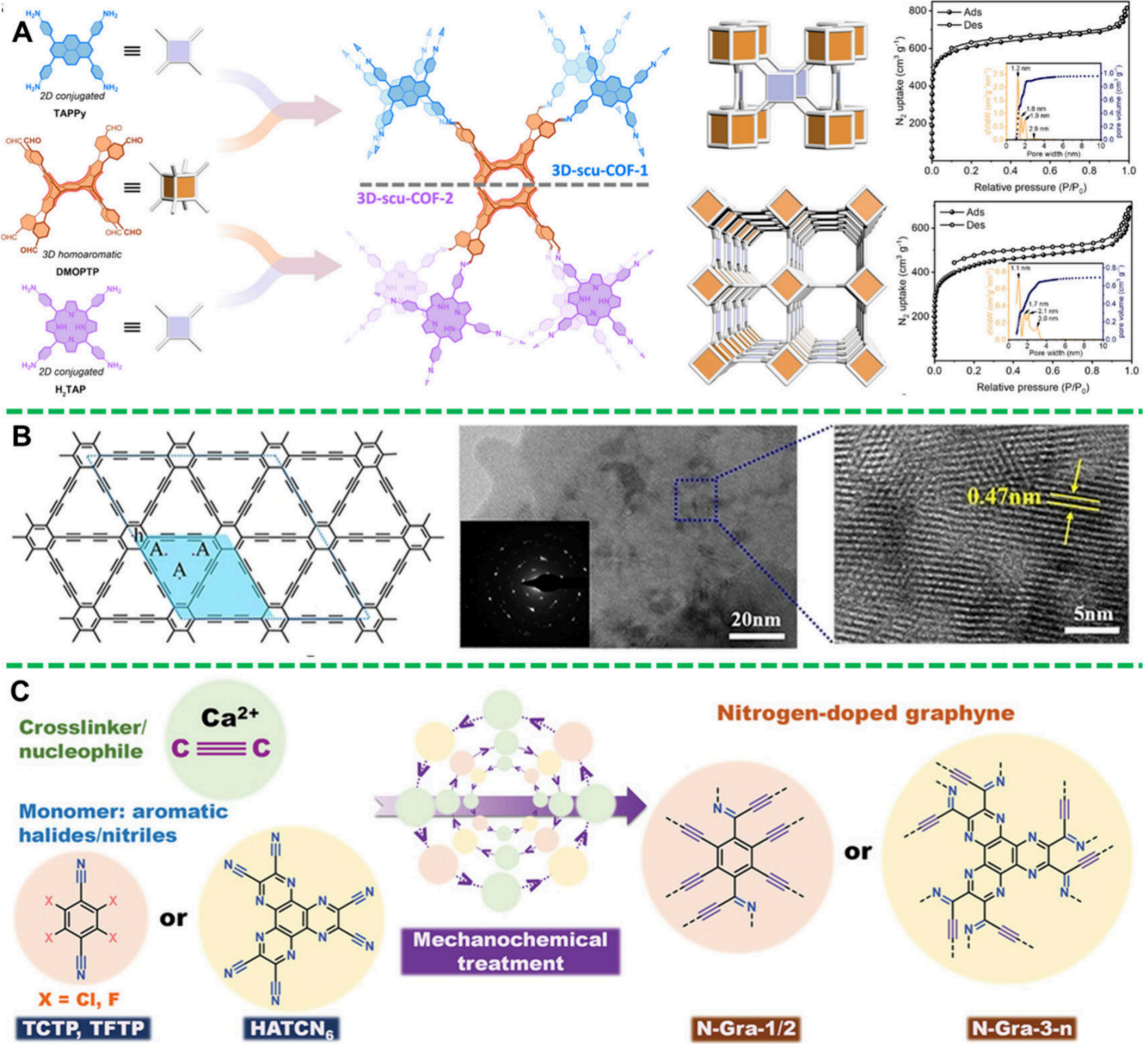
A) Schematic illustration of conjugated 3D high-connected COFs (3D-scu-COF-1/2).^462^ Reproduced with permission from ref 462. Copyright 2022 American Chemical Society. B) Graphdiyne anodes for fast-charging lithium-ion batteries.^463^ Reproduced with permission from ref 463. Copyright 2022 Wiley. C) Schematic illustration of the nitrogen-doped graphyne materials construction via the mechanochemical treatment pathway using CaC_2_ as the cross-linker and multiply substituted aromatic halides or nitriles as the starting materials.^464^ Reproduced with permission from ref 464. Copyright 2023 Wiley.

Graphdiyne, a carbon-rich two-dimensional material, consists of benzene rings connected via butadiyne (−C≡C–C≡C−) linkages and is typically synthesized through interfacial Glaser coupling of hexaethynylbenzene (Figure 34B). Variants can also be obtained via solution-phase or solid-state polymerizations, and different alkynyl precursors enable the construction of a range of GDY-based frameworks.^467–469^ Graphdiyne anodes exhibit self-expanding Li-ion transport channels enabled by reversible alkyne–alkene complex transitions during cycling, which significantly reduce Li-ion diffusion energy barriers and eliminate Li metal plating. This results in superior fast-charging performance, with a high capacity (342 mA h g^–1^ at 6 C) and ultralong lifespan (over 22,000 cycles), even at low temperatures.^463^

From a structure–performance perspective, π-conjugated carbon networks differ fundamentally from other synthetic routes in that their conductivity is not solely dependent on post-treatment graphitization, but is instead embedded within the conjugated backbone. At the same time, their intrinsic nanoporosity—derived from monomer geometry and network topology—provides well-defined ion transport pathways. This dual optimization allows these materials to mitigate the traditional tradeoff between electron transport and ion accessibility. These frameworks can be further tuned through heteroatom incorporation and thermal treatment. For example, nitrogen-doped graphyne scaffolds synthesized via mechanochemical cross-linking exhibit tunable nitrogen content (6.9–29.3 wt.%) (Figure 34C), hierarchical porosity, and enhanced capacitive behavior. Upon pyrolysis, these materials evolve into partially graphitized carbons while retaining heteroatoms, further improving conductivity and ion transport.^464^

Despite these advantages, a critical limitation lies in their synthetic complexity and scalability. Highly ordered π-conjugated frameworks (e.g., COFs and GDY) often require stringent reaction conditions and exhibit limited structural uniformity at scale, while CMP-derived systems, although more accessible, tend to be amorphous with moderate conductivity. This introduces a practical tradeoff between structural precision and manufacturability.

Overall, π-conjugated carbon networks can be viewed as a “structure-integrated” design paradigm, in which electronic conductivity and ion transport are coengineered at the molecular level. This distinguishes them from conductivity-driven (liquid alloy) and porosity-driven (mechanochemical) approaches, and explains their unique potential to simultaneously enhance both energy and power. However, realizing this potential in practical devices requires overcoming challenges in scalable synthesis and structural stability.

### 4.4 Machine-Learning Guided Material Design

Machine learning (ML) has significantly enhanced our ability to design synthetic porous carbon (SPC) materials by establishing complex nonlinear correlations between structural parameters and electrochemical performance metrics.^472^ This paradigm shift—from empirical trial-and-error methods to predictive, data-driven strategies—facilitates the rational tailoring of SPCs for fast-charging energy storage systems.

In this context, synthetic carbon nanostructures have emerged as ideal model systems for ML-assisted design, owing to their tunable hierarchical pore architectures, high electrical conductivity, and well-defined structure–property relationships. These features not only ensure rapid ion transport but also provide a robust platform for integrating pseudocapacitive components. For instance, hybridizing such conductive carbon frameworks with transition metal compounds (e.g., NiCo-layered double hydroxides, sulfides) can significantly enhance energy density through synergistic faradaic and nonfaradaic charge storage mechanisms, highlighting the importance of multicomponent design in next-generation supercapacitors.^473–479^ Furthermore, the emergence of quantum materials, including carbon quantum dots (CQDs) and MXenes, has introduced unique quantum confinement effects and expanded active sites, facilitating faster ion diffusion and improved rate capability.^480–482^ The frontier of this field now leverages density functional theory (DFT) to elucidate charge storage mechanisms at the atomic level and integrates machine learning algorithms (e.g., random forest and artificial neural networks) to optimize fabrication parameters and predict long-term cycling stability of these advanced synthetic carbon-based systems.^483,484^ For example, XGBoost models trained on extensive experimental data sets have identified key structural features such as specific surface area (SSA) (Figure 35A), the ratio of microporous to total surface area (Smicro/SSA), and pore size (PS) as dominant factors governing capacitance behavior in supercapacitors. These insights enable inverse design of SPCs with optimized hierarchical porosity, allowing researchers to prioritize structures that maximize ion-accessible surface area and charge storage efficiency, while significantly reducing experimental workload.^132^

**Figure 35:**
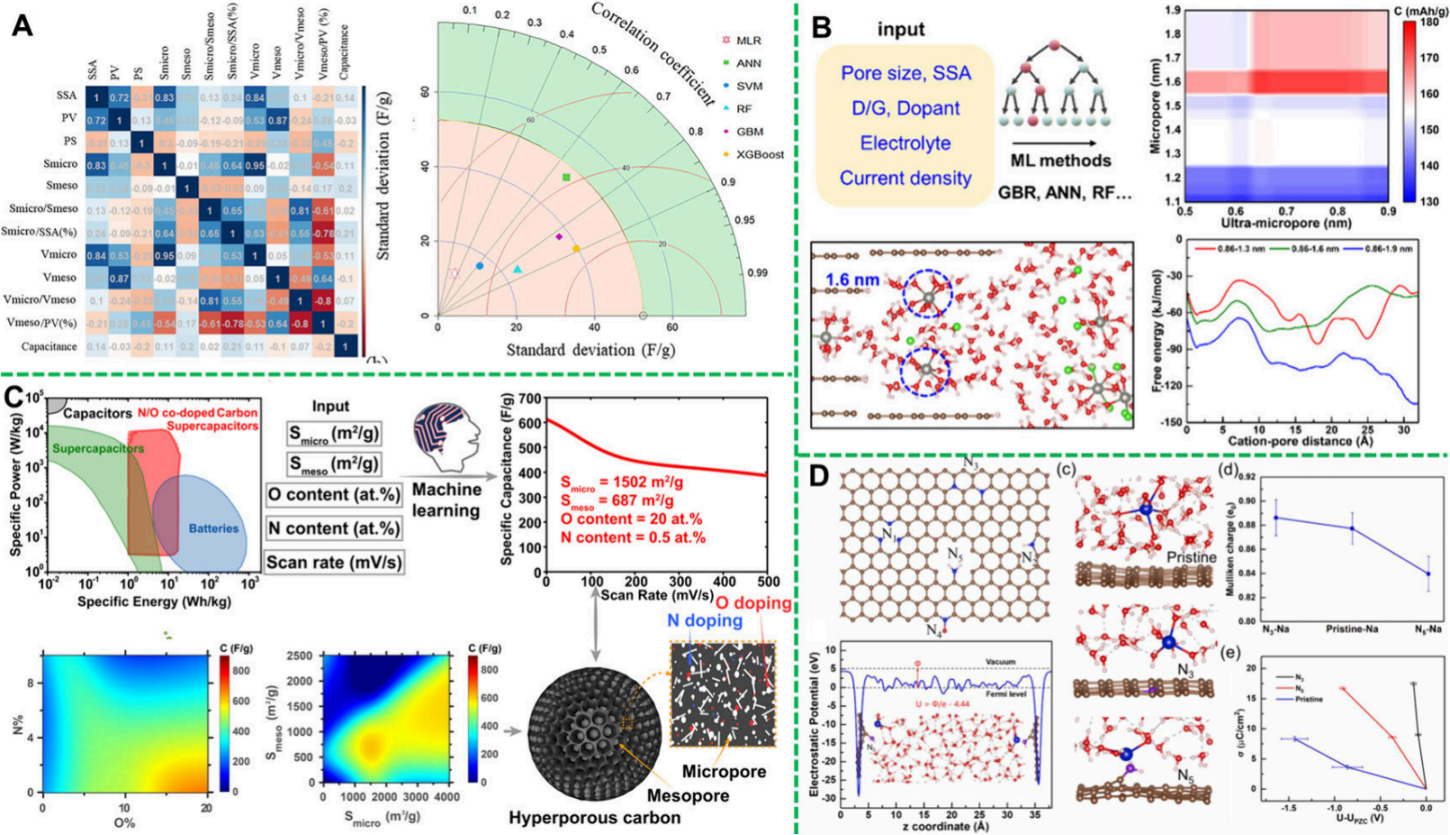
A) Machine learning–assisted design of porous carbon-based supercapacitor materials: Pearson correlation between structural features and capacitance; Taylor diagram comparing model predictions with observations.^132^ Reproduced with permission from ref 132. Copyright 2021 Elsevier. B) Design principles of gradient porous carbon for aqueous Zn-ion hybrid capacitors revealed by combined machine learning and molecular dynamics: optimal synergy of 0.6–0.9 nm ultramicropores and 1.6 nm micropores enhances ion storage and charging dynamics.^470^ Reproduced with permission from ref 470. Copyright 2025 American Chemical Society. C) Machine-learning-assisted design of oxygen-rich porous carbons for aqueous supercapacitors: Ragone plot comparison, ANN-predicted capacitance boundaries, and correlations of capacitance with heteroatom content and pore surface areas are shown.^32^ Reproduced with permission from ref 32. Copyright 2023 Springer Nature. D) Ab initio and machine-learning-accelerated molecular dynamics simulations revealing distinct roles of N3 and N5 doping in microporous carbon on ion transport and charge storage.^471^ Reproduced with permission from ref 471. Copyright 2024 Elsevier.

ML has also been applied to guide the design of gradient porous carbons by correlating pore structure characteristics—such as the distribution of ultramicropores (<1 nm) and micropores (1–2 nm)—with capacitive performance in energy storage devices (Figure 35B). A representative study on aqueous zinc-ion hybrid capacitors (ZIHCs) showed that a gradient porous carbon architecture combining 0.6–0.9 nm and ∼1.6 nm pores resulted in enhanced ion accessibility and promoted stepwise desolvation, thereby improving specific capacity and charging kinetics. ML-accelerated molecular dynamics (MD) simulations further confirmed that Zn^2+^ ions preferentially accumulate in 1.6 nm micropores, where their modified solvation shells enhance ion packing and surface charge balancing.^470^

Beyond pore size optimization, machine learning (ML) has emerged as a powerful tool for unraveling the interplay between heteroatom doping and electrochemical performance in SPCs. By integrating computational predictions with experimental synthesis, ML-guided frameworks have successfully identified nitrogen and oxygen codoping as key factors in enhancing charge storage behavior. For instance, Wang et al. employed a data-driven approach to predict that the maximum capacitance of N/O codoped activated carbon in 1 M H_2_SO_4_ electrolyte could be achieved at a micropore surface area of 1502 m^2^ g^–1^, mesopore surface area of 687 m^2^ g^–1^, oxygen content of 20 at.%, and nitrogen content of 0.5 at.% (Figure 35C). Guided by these targets, they synthesized hyperporous carbons (SSA up to 4476 m^2^ g^–1^) via sodium amide activation of hypercrosslinked polymers at a moderate temperature of 600 °C, preserving oxygen functionalities critical for both wettability and capacitance. The resulting material delivered an ultrahigh specific capacitance of 628 F g^–1^ at 0.2 A g^–1^.^32^ Complementary ML studies by Rahimi et al. further revealed that the optimized capacitance (∼500 F g^–1^) in 6 M KOH electrolyte could be achieved with a micropore area of ∼1400 m^2^ g^–1^, pyrrolic-N content of 2%, and carboxyl-O content of ∼8%, emphasizing the critical role of surface chemistry.^485^ Moreover, ML-accelerated ab initio molecular dynamics (AIMD) simulations have uncovered nuanced insights into the effect of specific nitrogen functionalities on ion transport within confined pore architectures. Graphitic nitrogen (N3), for example, was found to impede ion mobility in micropores due to steric hindrance, whereas pyrrolic nitrogen (N5) enabled hydrogen-bond-mediated electrolyte penetration (Figure 35D), thereby enhancing charge transfer kinetics.^471^ Zhou et al. studied the effect of functional groups that are grafted on porous carbons with different structural features, in the presence of aqueous KOH electrolyte, showing that pyridinic N can significantly improve the capacitance of carbon, whereas pyrrolic N has a negative effect on the capacitance at higher rates due to the high binding energy between pyrrolic N and potassium ions.^31^ These mechanistic revelations underscore the importance of incorporating both pore topology and local chemical environments in ML-driven material design.

In addition, ML has played a critical role in simulating the complex growth processes of carbon nanostructures on catalytic metal substrates such as Cu(111), Cr(110), and Ti(001), accurately predicting optimal synthesis conditions for defect-free carbon frameworks.^486^ Active ML models, integrated with molecular dynamics and force-biased Monte Carlo simulations, offer atomistic insights into growth pathways, enabling precise control over nanostructure formation through substrate-catalyzed synthesis. Beyond predictive modeling, ML is increasingly integrated with generative design tools and additive manufacturing techniques to propose and fabricate entirely new 3D porous carbon morphologies.^487,488^ Generative adversarial networks (GANs) and variational autoencoders (VAEs) have been used to generate topologically diverse carbon structures optimized for high surface area and rapid ion diffusion.^487^ These ML-generated architectures can be directly manufactured by using 3D printing, closing the loop between computational prediction and material realization.

Collectively, these advances highlight the transformative potential of machine learning in guiding both the design and synthesis of polymer-derived porous carbons for high-rate energy-storage applications, paving the way toward intelligent and efficient materials engineering beyond empirical boundaries.

Despite these promising advances, the application of machine learning to porous carbon design for electrochemical energy storage is still at an early stage and several challenges remain. One key limitation is the scarcity and heterogeneity of high-quality data sets, as experimental data are often collected under different testing conditions and reported using inconsistent descriptors, which complicates model training and validation. In addition, models trained on specific material systems or synthesis routes may exhibit limited transferability when applied to new carbon architectures, electrolytes, or device configurations. Another important challenge lies in the interpretability of complex machine learning models, particularly deep learning approaches, which can provide accurate predictions but often offer limited mechanistic insight into the underlying structure–property relationships. Recent studies have begun to address these issues by integrating data-driven approaches with advanced characterization and mechanistic investigations, including operando and NMR-based techniques that provide deeper insights into ion adsorption, desolvation behavior, and electrolyte–carbon interactions in porous electrodes. Future efforts should focus on constructing standardized and open databases, developing physics-informed machine learning frameworks, and integrating ML with high-throughput experimentation and theoretical modeling. Such strategies may ultimately enable closed-loop materials discovery and accelerate the rational design of next-generation porous carbon electrodes.

## 5 Advanced Characterization Technologies for Carbon-Based Supercapacitors

While conventional physicochemical characterization methods provide a solid framework for studying the surface chemistry and pore structure of electrode materials, they remain inherently limited in resolving the local carbon structure at the atomic and nanoscale. In particular, pore size distribution—one of the most critical parameters governing charge storage in carbon-based EDLCs—is typically derived from gas sorption isotherms using model-dependent approaches. These methods rely on the decomposition of adsorption data into kernels of partial isotherms based on a series of assumptions that may significantly deviate from the complexity of real porous systems. For instance, the Barrett–Joyner–Halenda (BJH) method assumes complete wetting of the surface, cylindrical and noninterconnected pores, while nonlocal density functional theory (NLDFT) similarly relies on idealized pore geometries and the assumption of pore independence. As a result, such approaches often fail to capture the hierarchical connectivity, irregular morphology, and dynamic accessibility of pores in realistic carbon materials.

Therefore, corroborating gas sorption results with complementary experimental techniques that do not rely on these restrictive assumptions is essential for developing a more accurate and physically meaningful understanding of charge storage behavior. Moreover, conventional characterization techniques are predominantly limited to ex situ analysis and thus cannot capture the dynamic and localized processes occurring at the electrode–electrolyte interface under operating conditions.

In this context, advanced characterization techniques—including X-ray and neutron scattering methods, Raman spectroscopy and spatially resolved Raman microscopies, as well as in situ and operando microscopies—have emerged as indispensable tools for bridging this gap (Figure 36). Among them, Raman spectroscopy is one of the most widely used tools for carbon materials because it is highly sensitive to defect density, graphitic ordering, and local structural disorder through the analysis of the D, G, and related second-order bands. In addition, confocal Raman microscopy and Raman mapping can resolve spatial heterogeneity in carbon frameworks, enabling visualization of local variations in disorder, graphitization, and heteroatom-associated structural features that are often obscured in bulk-averaged measurements. When combined with advanced characterization techniques and electrochemical analysis, Raman-based methods provide an important bridge between local carbon structure and charge-storage behavior in porous carbons. Small-angle X-ray scattering (SAXS) provides statistically robust information on pore size distribution and hierarchical structure without relying on simplified pore models, while quasi-elastic neutron scattering (QENS) enables direct probing of ion and solvent dynamics within confined nanopores, offering unique insights into diffusion mechanisms and confinement effects. Additionally, atomic force microscopy (AFM), particularly in electrochemical environments, allows for nanoscale mapping of surface morphology, mechanical properties, and interfacial processes with high spatial resolution.

**Figure 36:**
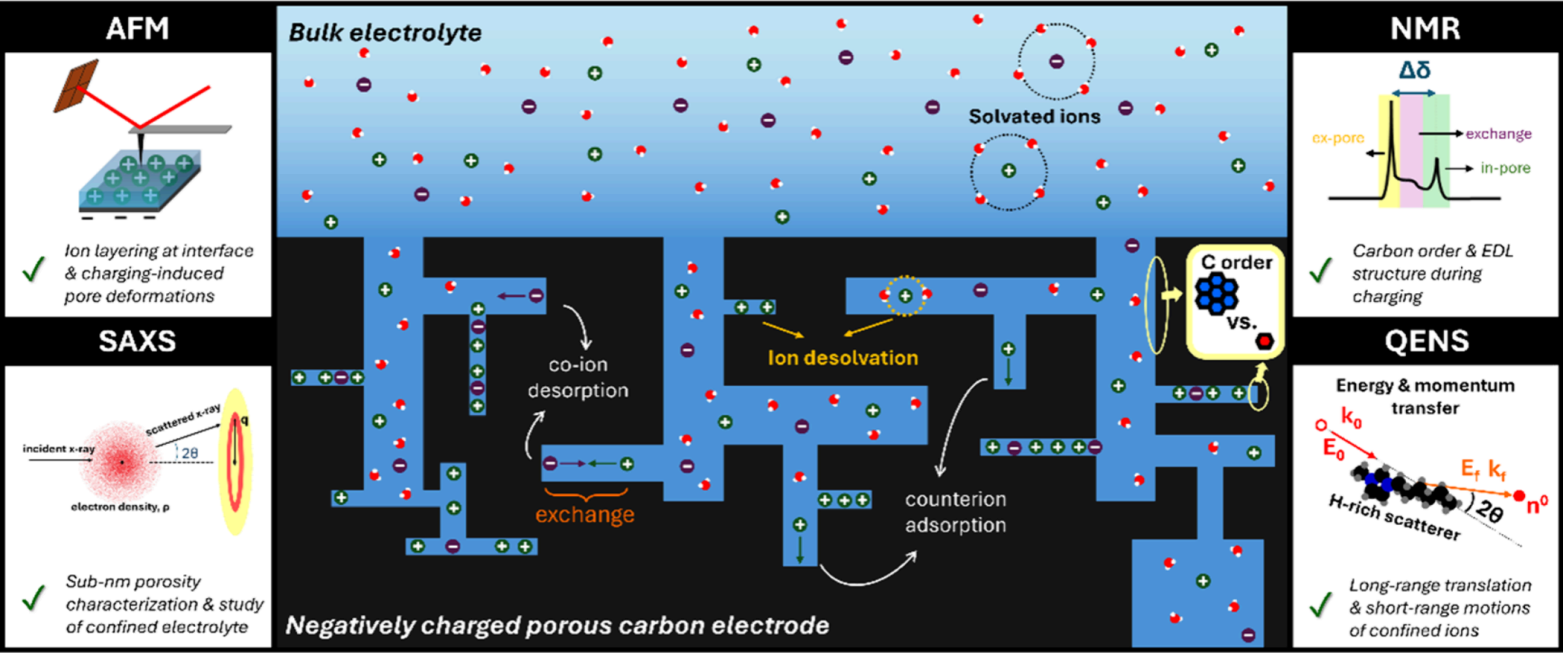
Illustration of processes that occur at the interface of a negatively charged porous carbon electrode with an electrolyte, during the EDL formation (ion desolvation, counterion adsorption, coion desorption, ion exchange), and some advanced characterization methods that enable the in situ study of the electrode–electrolyte interface.

Operando X-ray absorption spectroscopy (XAS) and in situ microscopies further enable real-time monitoring of electronic structure evolution and oxidation state changes under realistic electrochemical conditions. Meanwhile, in situ nuclear magnetic resonance (NMR) has proven especially powerful for directly probing ion adsorption, desolvation, and electrolyte–carbon interactions within confined pores. Together, these advanced techniques provide complementary structural and dynamical information across multiple length and time scales, enabling direct validation of theoretical predictions derived from ab initio and machine-learning approaches.^483^

By capturing the dynamic evolution of the electrode–electrolyte interface and resolving ion transport phenomena in realistic environments, these methods establish a critical link between idealized theoretical models and the actual charge storage behavior of porous carbons.

### 5.1 Raman Spectroscopy

Raman spectroscopy relies on the inelastic scattering of photons upon their incidence to matter to detect molecular vibration modes and is considered as the “workhorse” technique for the study of carbon structure and disorder, due to the high polarizability of delocalized π-electrons in sp^2^ carbon networks. The Raman spectrum of each carbon is unique, enabling discrimination not only between different allotropes (e.g., buckyballs_,_ carbon nanotubes, graphene, graphite, diamond), but also between different graphene structures (e.g., monolayered vs few-layered graphene stacks). Key features in the Raman spectra of graphitic carbons include the first-order G band (∼1580 cm^–1^), which is associated with the doubly degenerate in-plane transverse and longitudinal optical phonon (iTO/iLO) stretching modes, as well as the second-order, defect-independent 2D band (∼2700 cm^–1^) arising from a double resonance two-phonon scattering process near the Brillouin edge zone.^489^

While absent in ideal sp^2^ lattices of infinite size, the emergence of the second-order D band (∼1350 cm^–1^) in graphitic structures manifests the breaking of translational symmetry due to the presence of defects (Figure 37a), which may include vacancies, edge defects, topological defects, grain boundaries, sp^3^-type defects, or heteroatom doping, offering a pathway to assess defect density of carbon via calculating the D/G Raman band ratio. The presence of defects is particularly important in energy storage, as they typically exhibit enhanced reactivity and may act as electrolyte ion adsorption sites.^490,491^ Tuinstra and Koenig were the first to correlate the D/G Raman intensity ratio with the density of defects, establishing an inversely proportional dependence with the average crystallite size of graphite (I_D_/I_G_ ∼ 1/L_a_).^492^ Ferrari and Robertson showed that the Tuinstra-Koenig relationship fails at highly disordered carbons in which L_a_ is lower than ∼20 Å due to the higher sensitivity of the D band to amorphization, establishing a nonmonotonous dependence of the I_D_/I_G_ ratio on the density of one-dimensional edge defects, where I_D_/I_G_ ∼ L_a_^2^ in the highly defective regime.^493^ Later, Lucchese et al. studied the effect of zero-dimensional point defects on the I_D_/I_G_ ratio extracting a qualitative validation of Ferrari and Robertson’s model (Figure 37a, b), and concluding that increasing the distance of point defects, L_D_, up to L_D_ ≈ 4 nm increases the I_D_/I_G_ ratio, whereas when L_D_ > 4 nm the I_D_/I_G_ scales with the inverse square of L_D_.^494^ Another defect-activated Raman band is the so-called D’ band (∼1620 cm^–1^), which has been proposed to allow the distinction between different defect types through the calculation of the I_D_/I_D’_ ratio.^495^ Confocal Raman microscopy experiments on defective graphene by Novoselov and colleagues have shown that I_D_/I_D’_ becomes ∼13, ∼7, and ∼3.5 in the case of sp^3^-defects, vacancy-type defects, and grain boundaries, respectively.^496^ While the establishment of the above correlations has proven valuable in carbon research, it should be noted that these are often extracted from ideal systems (e.g., monolayer exfoliated graphene) in which well-defined defects are introduced (i.e., via Ar^+^ bombardment). On the contrary, porous carbons often possess a complex and heterogeneous combination of edges, vacancies, topological defects, and functionalities, which may all be probed at the same time due to the limited spatial resolution of conventional Raman spectroscopy, resulting in a convoluted Raman response that limits the quantitative reliability of the above ratios. Hence, these should be interpreted as qualitative descriptors of disorder unless supported by complementary characterization techniques.

**Figure 37:**
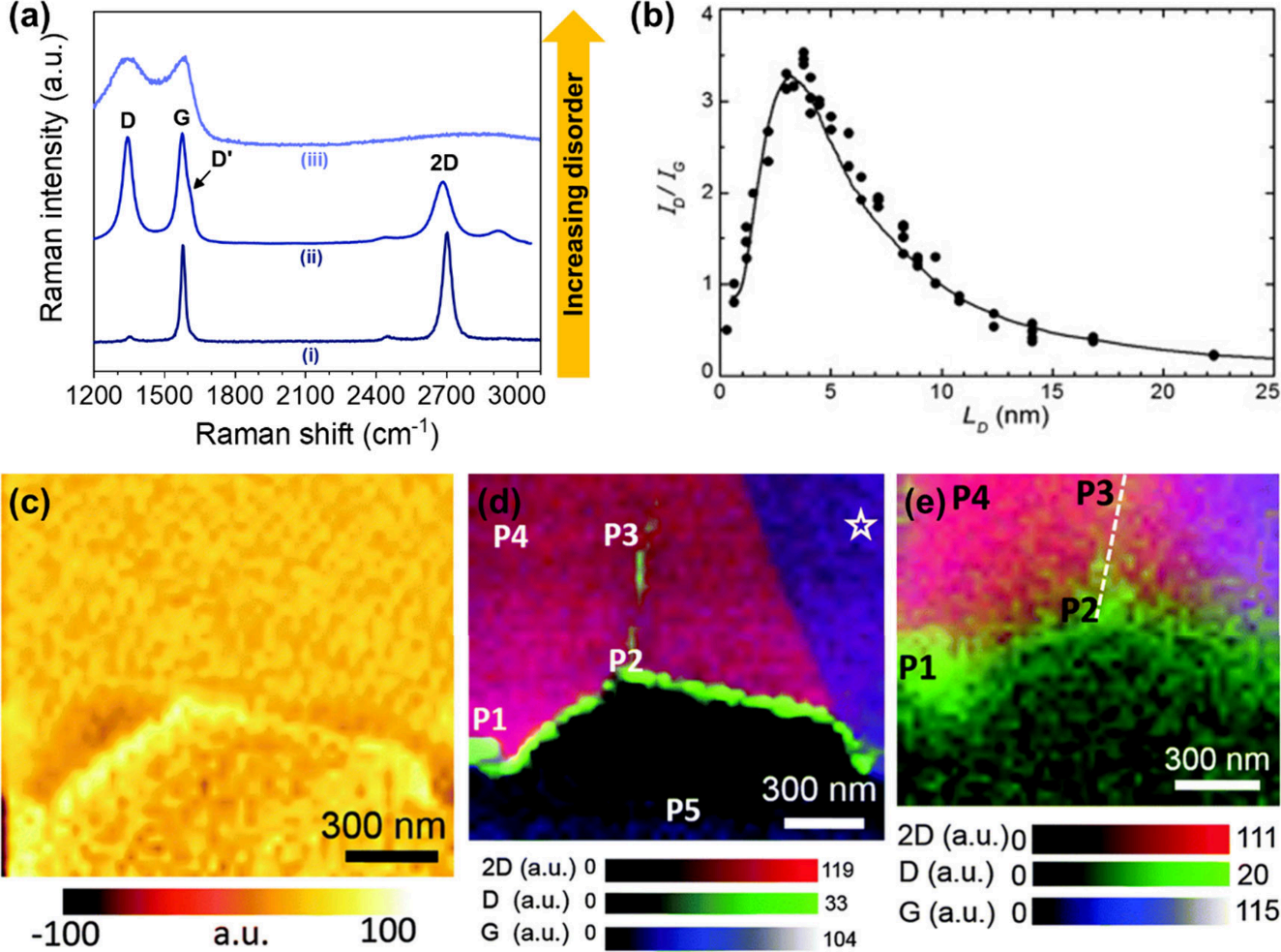
(a) Typical Raman spectra of carbon networks, where (i), (ii), and (iii) correspond to graphene networks of low defect density, highly defected graphene networks, and amorphous carbon, respectively. (b) Dependence of I_D_/I_G_ Raman band intensity ratio on the average distance of point defects (L_D_) for the case of monolayer graphene.^494^ Reproduced with permission from ref 494. Copyright 2010 Elsevier. (c, d) Atomic force microscopy topography image of a monolayer/bilayer graphene boundary and corresponding TERS mapping (pink/purple region: monolayer/bilayer graphene). (e) Confocal Raman spectroscopy mapping of the region in (d) shows the inferior spatial resolution compared to TERS.^497^ Reproduced with permission from ref 497. Copyright 2016 Royal Society of Chemistry.

To overcome the above-mentioned limitations of conventional Raman spectroscopy, advanced spatially resolved variants of Raman spectroscopy can be employed to map carbon heterogeneity.^498,499^ For instance, combining conventional Raman spectroscopy with confocal microscopy achieves the removal of the defocused part of the laser beam, probing a shrunken and well-defined focal volume within the sample, which enables a detailed mapping of carbon heterogeneity.^500–502^ Despite its improved spatial resolution (submicrometer scale), confocal Raman microscopy is fundamentally constrained by the diffraction limit of light, which enforces a loss of information when an object becomes smaller than the laser wavelength.

To break free from the light diffraction limitation of Raman imaging, Tip-Enhanced Raman Spectroscopy (TERS) can be employed. TERS combines Raman spectroscopy with scanning probe microscopy that implements a metallic tip (typically Au, Ag, or Al of granular morphology). This approach relies on the excitation of localized surface plasmon resonances at the metallic tip upon laser incidence, resulting in amplification of the Raman signal from a nanoscale region of the sample, enabling extremely high spatial resolution in the order of ∼10 nm, or even lower.^503^ Such high spatial resolution is important, as it enables the mapping of intrinsic material properties. Yano and colleagues used TERS to measure localized strain across the length of an isolated single-walled carbon nanotube, leveraging the strain-induced splitting of the doubly degenerate G band into the G^+^ and G^–^ bands.^504^ TERS has also proven to be powerful in the study of defects in graphene. For instance, Su et al. detected an intrinsic line-defect in monolayer graphene (Figure 37c,d), which was assigned to a mixture of disordered sp^2^ carbon and linear dislocation induced strain, and was invisible in their atomic force microscopy topography map.^497^ Park et al. revealed twisted bilayer structures of grain boundaries in polycrystalline monolayer graphene and identified misorientation angles in the overlapping regions of different crystal facets.^505^

### 5.2 Quasi-elastic Neutron Scattering (QENS)

QENS relies on the measurement of the energy and momentum transfer between the highly penetrative, H-sensitive neutrons and their scatterer species to extract information over the complex structure and dynamics (on nano- to picosecond time scales) of the electrode–electrolyte interface with nanoscale spatial resolution, providing critical insights on the processes responsible for charge storage.^508^ As the electrolyte’s mobility in the electrode–electrolyte interface correlates with the performance of a supercapacitor, QENS has received considerable attention in the experimental study of matrix-confined electrolytes, especially of hydrogen-rich ionic liquid (IL) cations that are confined in carbons with well-defined pore structure, such as CDCs or ordered mesoporous carbon matrices.^506^

Chathoth and colleagues were the first to study a mesoporous (8.8 nm pore size) carbon-confined IL ([bmim^+^]­[Tf_2_N^–^]) via QENS, revealing the occurrence of two distinct relaxation processes, namely of a slow long-range translational diffusion process, and of a faster process assigned to spatially localized mobility.^506^ Counterintuitively, the hydrogen-bearing cations that are not immobilized near the pore walls exhibit enhanced rather than suppressed diffusivity (manifested in the confinement-induced quasi-elastic spectra broadening, and the decay of I­(q,t)/I­(q,0) in Figure 38a), which was assigned to the increased density of cations near the pore walls and their decreased density in the middle of the pores. QENS has been also employed for the study of imidazolium-based ILs of variable cation size.^509–511^ To assess the influence of the mesopore-confined (pore size 2–10 nm) ILs’ alkyl chain length on the charging behavior, ion arrangement and dynamics, Osti et al. compared long (12-carbon, C_12_mim^+^) and short (3-carbon, C_3_mim^+^) cations (using TFSI^–^ as the common anion).^509^ Their findings suggest that while long alkyl chains decrease long-range cation diffusivity, longer chains exhibit higher localized mobility as they can undulate and move independently of the imidazolium ring, potentially benefiting ion exchange phenomena at the electrode–electrolyte interface. In addition, if a critical voltage threshold is exceeded, cation concentration of the C_12_mimTFSI system becomes depleted in pores, and capacitance of the larger ion system becomes lower than that of the C_3_mimTFSI. Similarly, Dyatkin et al. changed the cation chain length from an ethyl (n = 2) to a butyl (n = 4) to a hexyl (n = 6) group to assess the dynamics and accumulation densities of [C_n_mim^+^]­[TFSI^–^] confined in CDC (0.8 nm pore size), reporting a monotonous diffusion coefficient and jump length decrease for both localized motions and long-range translation as the chain length increases.^510^ While the replacement of [C_2_mim^+^]­[TFSI^–^] with [C_4_mim^+^]­[TFSI^–^] slightly reduced the diffusion coefficients and jump lengths, their values were significantly decreased when [C_4_mim^+^]­[TFSI^–^] was replaced with [C_6_mim^+^]­[TFSI^–^]. Apart from the case of endohedral carbon network-confined ILs, QENS has been used to study the effect of the IL’s cation size on the high-rate capability of a system using exohedral carbon nano-onions.^511^ The increasing cation size, transcending from [EMIM^+^]­[Tf2N^–^] to [HMIM^+^]­[Tf2N^–^] and to [BMIH^+^]­[Tf2N^–^], negatively affected the IL diffusion coefficient, resulting in a more pronounced capacitance drop at higher charging/discharging rates.

**Figure 38:**
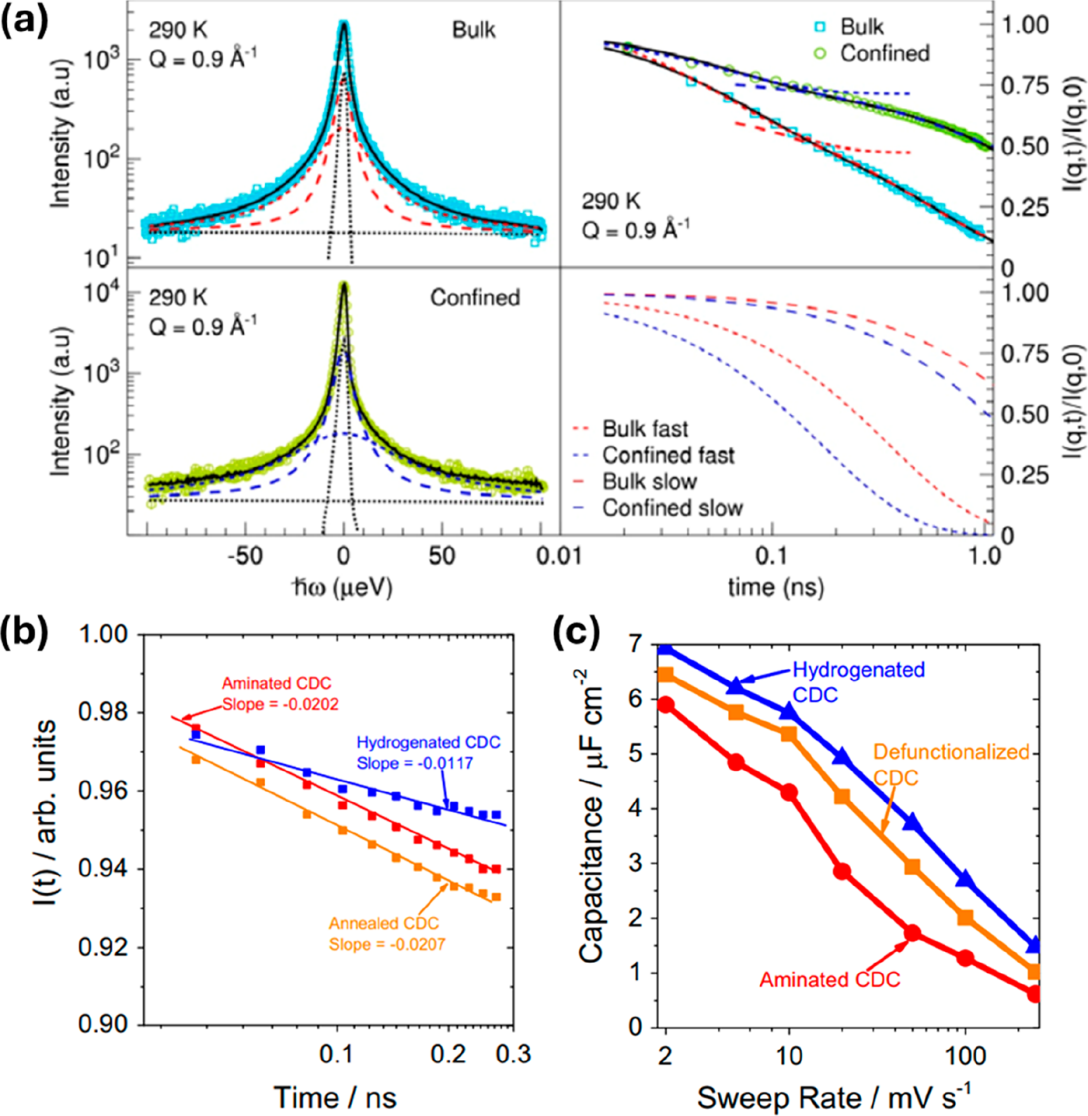
(a) Fitted QENS spectra of bulk and confined [bmim^+^]­[Tf_2_N^–^]. Normalized Fourier transformed QENS spectra of bulk and confined IL along with their fits.^506^ Reproduced with permission from ref 506. Copyright 2012 IOP Publishing. (b, c) Logarithmic decay relationship between relaxation time and the incoherent scattering function of CDC-confined [EMIM^+^], and capacitance dependence on the cyclic voltammetry scan rate for the CDC/[EMIM^+^]­[TFSI^–^] systems.^507^ Reproduced with permission from ref 507. Copyright 2015 Elsevier.

Besides the variable ion to pore size ratio, the surface chemistry of the carbon matrix can significantly affect its interactions with the electrolyte’s ions, and thus the electrochemical performance. To isolate the influence of the electrode’s functional groups, Gogotsi and colleagues studied the mobility of [EMIM^+^]­[TFSI^–^] confined in TiC-derived carbons of same pore structure (mostly microporous, with pore size of 0.6–0.7 nm), but of different surface chemistry, namely of a defunctionalized CDC, a hydrogenated CDC, and an aminated CDC.^507^ Among the self-correlation functions for [EMIM^+^] cations in various environments, the I­(t) for the H-rich CDC decayed the slowest (see Figure 38b), suggesting that ions confined in H-functionalized pores have the lowest mobility, manifesting their strongest intermolecular interactions. As a consequence, the H-rich CDC system exhibited the highest capacitance throughout the whole range of scan rates (Figure 38c). On the contrary, comparison between the defunctionalized and N-rich CDC indicated the absence of favorable interaction, providing a rationale for the inferior performance of aminated CDC. The same group investigated the influence of commonly encountered oxygen-containing functional groups on the capacitance and electrosorption dynamics of [EMIM^+^]­[TFSI^–^] onto porous CDC and nonporous graphene nanoplatelet (GNP) electrodes.^512^ Unlike the case of planar GNPs, where O-functionalization decreased capacitance, the oxygen functional groups increased the capacitance and high-rate capability in the case of the porous CDC electrode. The attraction of oxygen groups to [TFSI^–^] outweighed their repulsion of hydrophobic [EMIM^+^] cations, resulting in an overall increased ionophilicity of the oxygen-rich pore walls, benefiting both the electrosorption and the self-diffusion of [EMIM^+^]­[TFSI^–^]. Similarly, oxidation of a molybdenum carbide-derived carbon matrix possessing both micropores and mesopores improved the mobility of a confined [OMIM^+^]­[TFSI^–^] IL; however, contrary to the previous study on the CDC/[EMIM^+^]­[TFSI^–^] system,^512^ this mobility improvement did not result to a capacitance enhancement, but rather to a declined performance.^513^ While oxygen functional groups enhanced the interface of [TFSI^–^] with the CDC, they repelled the hydrophobic [OMIM^+^] cations, decreasing the density of electrosorbed ions.

### 5.3 Small Angle X-ray/Neutron Scattering (SAXS/SANS)

SANS and SAXS are nondestructive and complementary techniques that rely on the scattering of neutrons and X-rays to provide structural information on the nanoscale.^514,515^ In SAXS, X-rays interact with the electron density and therefore depends on the atomic number of the system’s components, whereas in SANS, neutrons interact with nuclei, being sensitive to light elements and to isotopic substitution. In both techniques, the signal intensity grows with the contrast between the porous carbon network and its voids and thus drops after introducing a medium (e.g., an electrolyte) in these voids. *In-situ* application of small-angle scattering methods has already proven its high value for the study of the electrochemical behavior of cells that incorporate porous carbon electrodes.^516,517^

Following the study of Iiyama et al., who studied the adsorption of water molecules on hydrophobic activated carbon fibers, reporting the formation of small clusters irrespective of carbon’s pore width,^518^ *in situ* SAXS has received considerable interest in the examination of porous carbon electrodes that are filled with an electrolyte, in the absence or presence of polarization. Ruch et al. used SAXS to correlate the capacitance gain after electrochemical activation of mesophase pitch-derived carbon to the electrochemically driven irreversible insertion of ions into previously inaccessible void space, which could not be explained by gas sorption techniques.^519^ Bañuelos et al. used both SAXS and SANS to investigate the molecular-scale properties of [C4mim^+^]­[Tf2N^–^] IL confined within a hierarchical nanoporous carbon network, reporting on the preferential filling of subnanometer slit-like micropores along the mesopore surfaces, with the micropore confinement resulting in a low excluded volume and an ion density that is higher than that of the mesopores or the bulk electrolyte phase.^520^ Prehal and colleagues combined operando SAXS with X-ray transmission to probe the structural arrangement of various aqueous alkali metal chlorides confined in activated carbon, showing that at low charge density the total number of ions (cations plus anions) remains constant during charging/discharging (ion swapping), whereas at high charge density, charging involves both counterion adsorption and local in-pore ion rearrangement.^521^ The same group presented a synergistic approach of interpreting *in situ* SAXS experiments with the help of Monte Carlo simulations (Figure 39) to study the voltage-dependent specific arrangement of ions in aqueous electrolyte within the pores of microporous carbons, and concluded that the capacitance is affected not only by carbon’s pore size, but also by the degree of ion confinement in micropores (DoC), the degree of ion desolvation (DoDS, occurring even in mixed micro–mesoporous carbons with pore size well above 1 nm), as well as carbon’s pore size dispersity.^522^

**Figure 39:**
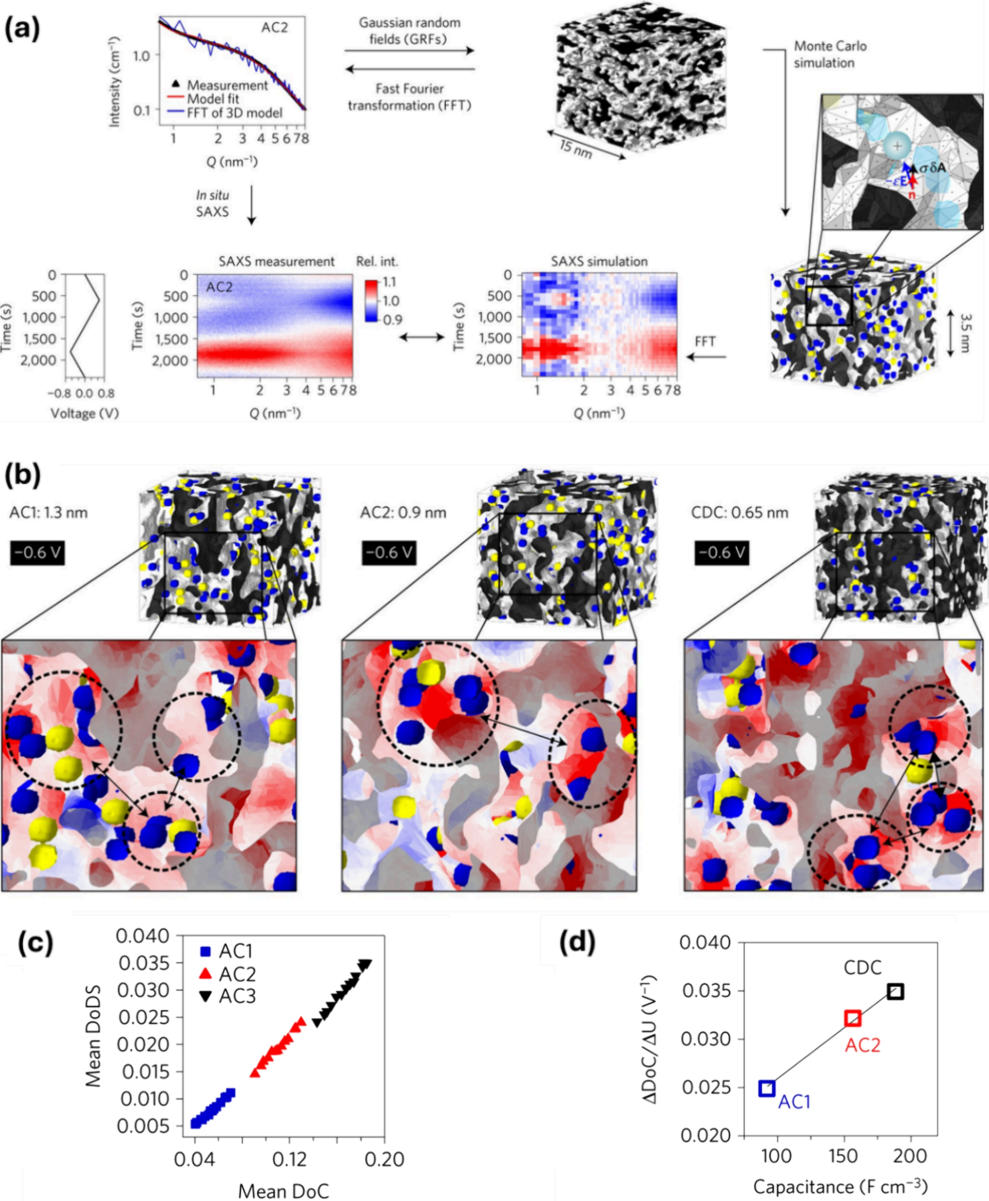
(a) Combined in situ SAXS and modeling approach. SAXS intensity versus scattering vector length (Q) results in a real-space pore structure using the concept of Gaussian random fields. For each electrode voltage, cations and anions (blue and yellow) rearrange according to a Monte Carlo simulation. Weighting of ions, water and carbon phase by their respective electron density and subsequent FFT provides the simulated SAXS intensity, which is compared to its experimental counterpart. (b) Ion arrangement near high negative surface charge density (red) shows more localized charge storage for the CDC electrode (right). (c) Linear correlation of DoDS and DoC. (d) Linear dependence of mean DoC with the micropore volume-normalize capacitance.^522^ Reproduced with permission from ref 522. Copyright 2017 Springer Nature.

Similarly to SAXS, SANS has also proven to be a powerful tool in the study of microporous carbon. For instance, Gogotsi and colleagues compared gas sorption with SANS measurements from TiC-derived carbons, reporting that the former overestimates the actual pore size in the case of low temperature chlorination, due to the presence of inaccessible small pores and because of the nearly spherical shape of microporous CDC (as density functional theory models assume nonspherical pores).^523^ The research group of Yushin used SANS to monitor the hydrogen distribution changes in nanoconfined aqueous H_2_SO_4_ electrolyte (in H_2_O and D_2_O) upon the application of an external potential to binder-free activated carbon fabric electrodes, providing insight on the electrowetting phenomenon in subnanometer pores under an applied potential, which can counterbalance the insufficient carbon wetting by the solvent.^517^ Unlike the case of a H_2_O solvent, where the whole carbon surface was wetted by the electrolyte, D_2_O could not access the smallest pores, greatly diminishing proton adsorption at these sites (decreasing capacitance). The limited (of ∼20%) infiltration of aqueous NaCl electrolyte in microporous carbon electrodes was also reported by Liu et al. following their study in aqueous electrolytes,^524^ Yushin and colleagues investigated the case of an organic tetraethylammonium tetrafluoroborate (TEATFB) salt in deuterated acetonitrile (d-AN), observing an increase of the specific capacitance at higher applied potentials, when the smallest pores become more accessible to electrolyte.^525^

### 5.4 Atomic Pair Distribution Function (PDF) Analysis

Porous carbons are typically characterized by long-range disorder (poor crystallinity), as is manifested by the broad Bragg peaks in the X-ray diffractograms of these materials. However, diffuse scattering enables the extraction of a pair distribution function (PDF), G­(r), which is obtained through Fourier transform of the measured structure factor, S­(Q), where Q is the momentum transfer of the scattered particles (e.g., neutrons, X-rays), and represents the probability of finding atom pairs separated by a distance r. As the PDF provides the atomic structure of nonperiodic materials through a real-space measure of the locations of near-neighbor atoms,^526^ the structural insights offered by this method are valuable for assessing the short-range order of porous carbons, which varies depending on the synthetic route. According to Kane et al., increasing the pyrolysis temperature of poly­(furfuryl alcohol)-derived carbon transforms the porous carbon from a disordered structure to one that is more ordered on a length scale of 15 Å, with a temperature of 1200 °C resulting in a PDF that matches better to that of graphene than that of graphite.^526^ Smith et al. relied on a computational approach to study the atomic structure of nongraphitizing carbons by introducing a small percentage of 5-membered and 7-membered rings into sp^2^ carbon layers, with a 1% nonhexagonal ring content, resembling the experimentally obtained PDF from poly­(furfuryl alcohol)-derived carbon pyrolyzed at 1200 °C.^527^ Increasing the chlorination temperature of TiC resulted in enlarged and less curved sp^2^ carbon domains in CDCs, with correlations extending to ∼20 Å (Figure 40a).^528^ Lower temperatures in the synthesis of porous carbon result in layers of higher defect density and increased curvature that reduce atomic coherence, in the limit of a relatively open structure which resembles neither that of trigonally coordinated and layered graphite nor that of tetrahedrally coordinated diamond.^529^

**Figure 40:**
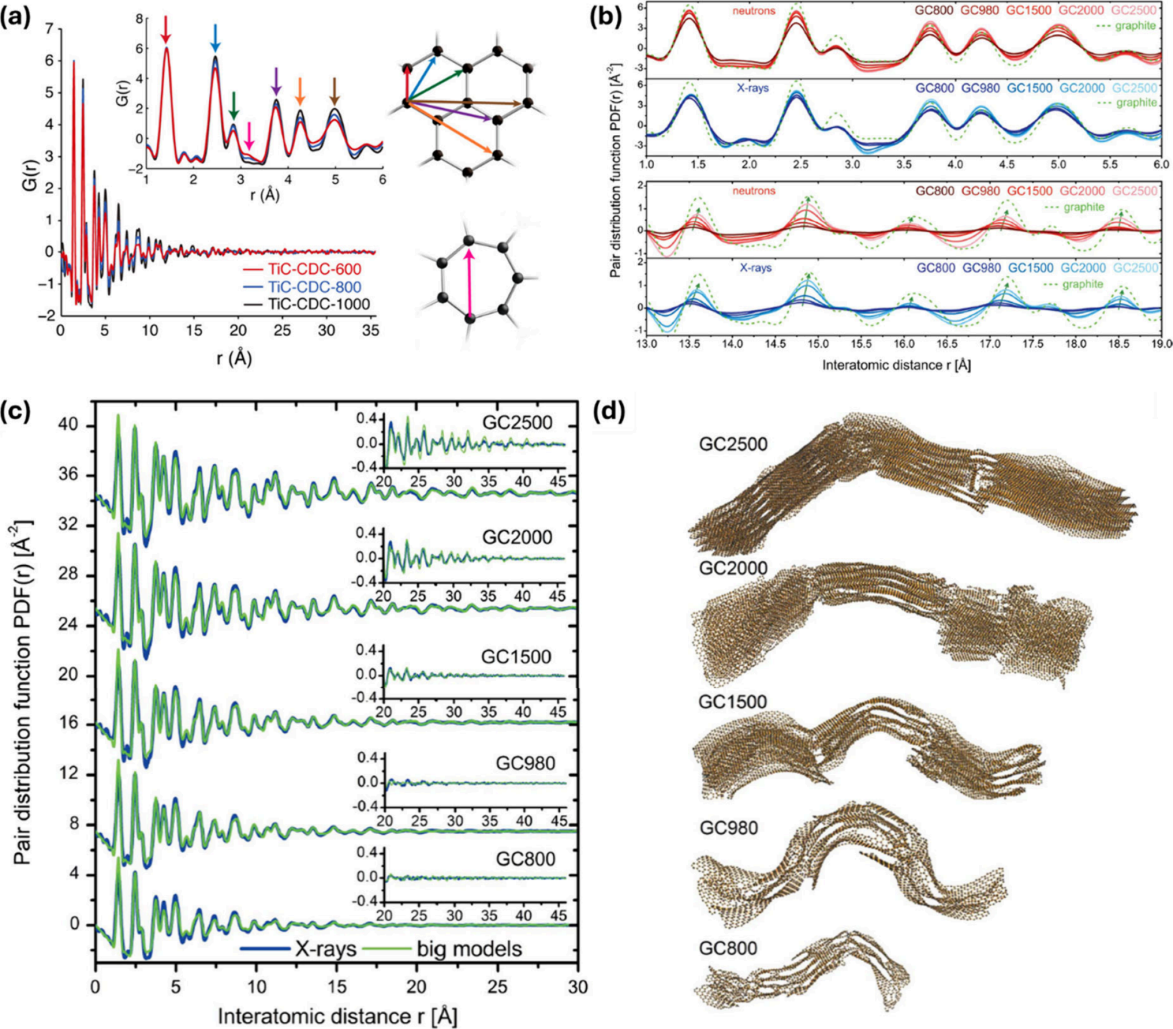
(a) X-ray PDFs from CDCs chlorinated at different temperatures. Inset shows the 1–6 Å short-range region and the assignments to various C–C correlations.^528^ Reproduced with permission from ref 528. Copyright 2015 American Chemical Society. (b) Comparison of interatomic distances obtained from experimental PDFs of glassy carbons obtained at different temperatures (800–2500 °C) with the ones that are calculated for graphite, in short-range and intermediate-range order. (c) Comparison of the PDFs computed for large-scale models with the ones experimentally obtained from X-ray scattering. (d) Visualization of the proposed large-scale models.^530^ Reproduced with permission from ref 530. Copyright 2017 International Union of Crystallography

Jurkiewicz et al. used a combined modeling and X-ray/neutron scattering approach to study the changes in the structure of nongraphitizing glassy carbons as a function of the pyrolysis temperature in the range of 800–2500 °C, demonstrating that at higher temperatures the glassy carbon structure approaches (but never reaches) the order of graphite, manifested by a gradual increase in C–C bond uniformity and domain size, as well as a decrease in curvature.^530^ As illustrated in Figure 40b,c, the short-range PDF peak positions are insensitive to the pyrolysis temperature and same as that of unstrained graphite, while their width decreases, whereas the intermediate-range PDF peak position shift toward longer interatomic distances as the temperature increases, approaching those of intralayer graphitic structure, suggesting a gradual flattening under the influence of a more intensive heat treatment. After assuming the absence of spatial correlations among the coherently scattering domains, to generate small models of these domains, the authors constructed larger models (Figure 40d) to account for domain cross-correlations, that may be associated with the formation of a porous structure.^530^

### 5.5 Nuclear Magnetic Resonance (NMR) Spectroscopy

NMR spectroscopy has become an increasingly popular analytical tool in porous carbon research, either *ex-situ* or *in situ*, as it can provide unique insights into the short-range ion environment of carbon. The method is based on the chemical shift (Δδ) of strongly adsorbed or confined molecules/ions, which is due to the shielding from the delocalized electron distribution of sp^2^ carbon domains. NMR spectroscopy exhibits significant advantages, such as the fact that it can be applied to both solids and liquids with no requirement for long-range order, its element specific nature, enabling the extraction of information for one species of interest without interference from the other components of the system, as well as its sensitivity to dynamics over a wide range of timescales.^160^

Gray and co-workers investigated the structure of CDCs using a combined NMR, Raman spectroscopy, and PDF analysis approach.^528^ Their NMR experiments showcased the sensitivity of chemical shifts to pore size distribution and aromatic carbon domain size, as: (i) the Δδ magnitude of TiC CDCs drops monotonously as their chlorination temperature decreases from 1000 to 600 °C, owing to the decreasing size of ordered domains, and (ii) high temperature (1400 °C) vacuum annealing post-treatment of CDCs resulted in a contrasting, but more subtle, increase in Δδ as the TiC chlorination temperature drops from 800 to 400 °C (Figure 41a–c).^528^ Recently, leveraging findings of the previous study, Forse et al. evaluated a large series of commercial nanoporous carbons, with capacitance values that did not correlate with their surface area, pore size, or heteroatom content, demonstrating a strong correlation of capacitance with carbon’s structural disorder, probed by Δδ (see Figure 41d,e).^90^

**Figure 41:**
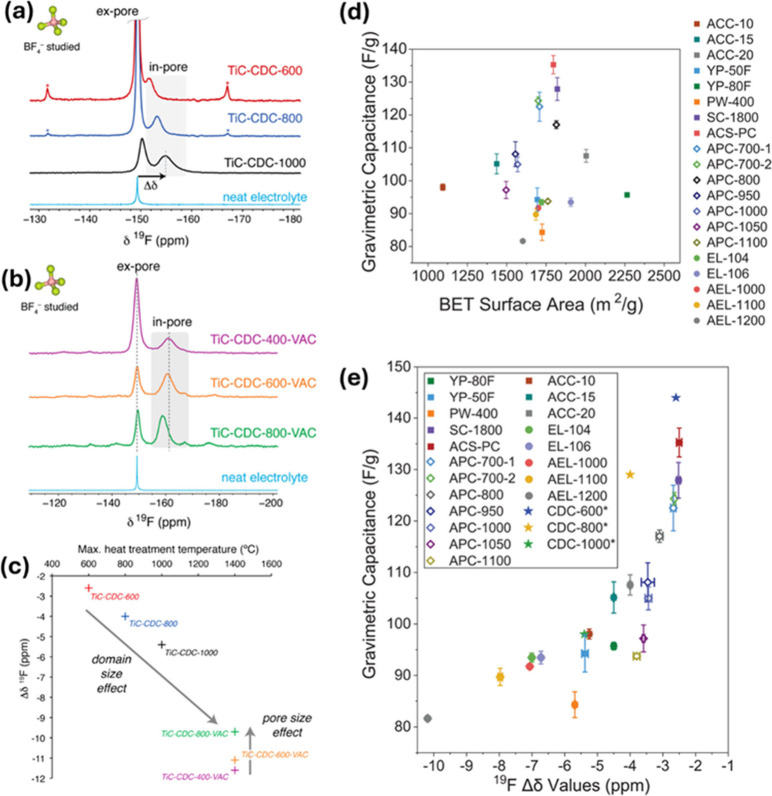
^19^F MAS NMR spectra of TiC-derived CDCs (soaked with NEt_4_BF_4_/dACN) (a) obtained at different chlorination temperature, and (b) chlorinated at different temperature, but subjected to vacuum annealing post-treatment at 1400 °C.^528^ Reproduced with permission from ref 528. Copyright 2015 American Chemical Society. (c) Domain size and pore size effects on Δδ ^19^F values.^528^ Reproduced with permission from ref 528. Copyright 2015 American Chemical Society. (d) Lack of correlation between the specific capacitance and the BET SSA of porous carbons, (e) Correlation of specific capacitance with ^19^F Δδ values of porous carbons.^90^ Reproduced with permission from ref 90. Copyright 2024 American Association for the Advancement of Science.

Lee et al. used magic angle spinning (MAS) NMR to study the adsorption behavior of an organic electrolyte anion, tetrafluoroborate, on activated carbon electrodes of an EDLC (at open-circuit voltage, charged, and discharged state), distinguishing between the anions located outside and inside the pores.^531^ Gray and colleagues studied the charge storage behavior of supercapacitors via *in situ* NMR, concluding that charging can be divided into two distinct regimes depending on the cell voltage.^532^ At low cell voltages, anions desorb from the carbon micropores at the negative electrode, while at the positive electrode there is no significant increase in the adsorption of anions as the voltage increases. On the other hand, above a certain voltage threshold, adsorption of anions in the positive electrode increases significantly, while anions continue to desorb from the negatively charged carbon electrode. Borchardt et al. used NMR to study the interaction of an organic TEABF_4_ electrolyte with various carbons of well-defined porosity (CDCs, mesoporous carbon, and hierarchical carbon combining mesopores/micropores), concluding that the chemical shift of NMR-active nuclei of molecules adsorbed in carbon of comparable sp^2^ content correlates with pore size, with molecules being increasingly immobilized in smaller pores.^533^ One NMR signal was recorded for all carbons regardless of a unimodal or bimodal pore size distribution, suggesting the rapid movement and exchange of adsorbed molecules inside the pores. Unlike the case of larger pores, where solvent evacuation increased the contact of the electrolyte with the pore walls, there was no increase of the diamagnetic shift for pores that are smaller than 1 nm, supporting that the electrolyte molecules do not possess an intact solvent shell in such small pores. Fulik et al. applied two-dimensional exchange spectroscopy, further elucidating the exchange kinetics of TEABF_4_/acetonitrile in microporous, mesoporous, and hierarchical carbons.^148^ By measuring the mixing time dependence of the intensity ratio between cross and diagonal peaks, two different types of exchange processes were distinguished, namely, a fast process that is due to the exchange at the interface between carbon particles and the surface layer of the surrounding bulk electrolyte, and a slow process that is governed by the diffusion of the electrolyte molecules inside the particles. For both processes, solvent molecules exchange substantially faster than ions, with cations being the slowest, while the presence of mesopores was found to promote faster ion exchange.

Griffin and colleagues used NMR spectroscopy to quantify the local concentration of alkali ions spontaneously adsorbed on activated carbon in the absence of applied potential, reporting that at low electrolyte concentrations the ions preferentially occupy the larger pores where there is less distortion of the solvation shell.^534^ Deschamps et al. used *ex-situ* MAS NMR spectroscopy to study the organization of an organic TEABF_4_ electrolyte in activated carbon electrodes after their charging, revealing the presence of two distinct adsorption sites both of which undergo chemical exchange with the free electrolyte molecules.^535^ At both electrodes, charging results in substitution of coions by counterions, the proportions of which were quantified by NMR, whereas at the negative electrode there is also removal of acetonitrile molecules due to the larger size of the incoming TEA^+^ cations (in relation to removed BF_4_^–^ anions).

### 5.6 Atomic Force Microscopy (AFM)

Atomic force microscopy (AFM) relies on the deformation (such as bending or torsion) of the AFM cantilever as a result of the interaction of its ultrasharp tip with the scanned sample. Throughout scanning, this variable deformation is detected through the change in the deflection of a laser beam that is reflected off the cantilever’s backside. Quantification of this deflection enables the mapping of the sample’s topography (as well as of various physicochemical properties, depending on the specific AFM mode) with high vertical resolution of a few angstroms and lateral resolution of a few tens of nanometers.^536^ Thus, coupling of AFM with an electrochemical cell and a potentiostat renders this technique an indispensable tool for the *in situ* and *operando* study of electrochemomechanical phenomena at the electrode–electrolyte interface, such as the cracking of the solid-electrolyte interface in Li-ion battery anodes due to volume variations.^537^

Considering EDLCs, *in situ* AFM force–distance measurements can detect both the ion arrangement on a surface and the layered structure of the EDL, provided that the electrode surface is atomically flat.^167,538^ Black et al. studied the reconfiguration and orientational transitions of the Stern layer upon applied bias in a [Emim^+^]­[Tf_2_N^–^]/HOPG (highly oriented pyrolytic graphite) interface, reporting an almost complete screening of the charged electrode by counterions within 1.0 nm from the interface.^539^ Similarly, Elbourne et al. used i*n-situ* amplitude-modulated AFM to reveal the lateral structure of the Stern layer at the interface of [EMIM^+^]­[TFSI^–^] IL with a HOPG electrode.^540^ While well-defined anion–cation–cation–anion rows were observed at an open-circuit potential, application of a small positive potential rearranges cation double rows to single rows as the anion domains grow wider, whereas increasing this positive potential indicates the formation of a dense but amorphous counterion (anion) arrangement. The lateral structure of the Stern layer evolved differently at negative applied potentials, with the counterions (cations) attaining a disordered arrangement at low potentials, transcending to well-defined and elevated cation double rows at higher potentials. On the other hand, an *in situ* AFM study of the in-plane and out-of-plane EDL structures of [PYR_14_^+^]­[TFSI^–^] on HOPG by Tsai et al., revealed that the first adsorbed layer consists of both disordered and ordered lateral domains of rectangular shape, submicron size and complex periodicity, with preferred domain orientations of 120° with respect to each other (Figure 42 a-c), while the out-of-plane EDL structure was found to be independent of the in-plane structure.^541^ The ordered domains disappeared outside a certain applied positive or negative potential, in the form of a hysteretic order–disorder transition (Figure 42d). The group of Balke observed the internal structure of defects on the interface of [Emim^+^]­[Tf_2_N^–^] IL with a HOPG electrode, demonstrating that graphite’s step edges induced only a short-ranged disordering of the ion layers at the interface, while dislocation-type topological defects were observed on planar surfaces (see Figure 42e,f).^538^ The layered IL EDL structure on HOPG was also studied by Zhou et al., observing pronounced molecular density variations within each layer, in the form of zigzag-like patterns with a quasi-periodicity of 1.5–2 nm. Upon bias, the first EDL layer exhibited the most significant reconfiguration. Specifically, changing the potential from 0 to ± 0.5 V, widened the first layer and enhanced zigzag oscillations, while at ± 1 V, the first layer split into two layers, with the lower layer approaching the HOPG surface (∼0.2 nm distance) and the upper layer being ∼0.5 nm above the lower layer (Figure 42g,h), manifesting the ion rearrangement as response to the applied potential.^542^

**Figure 42:**
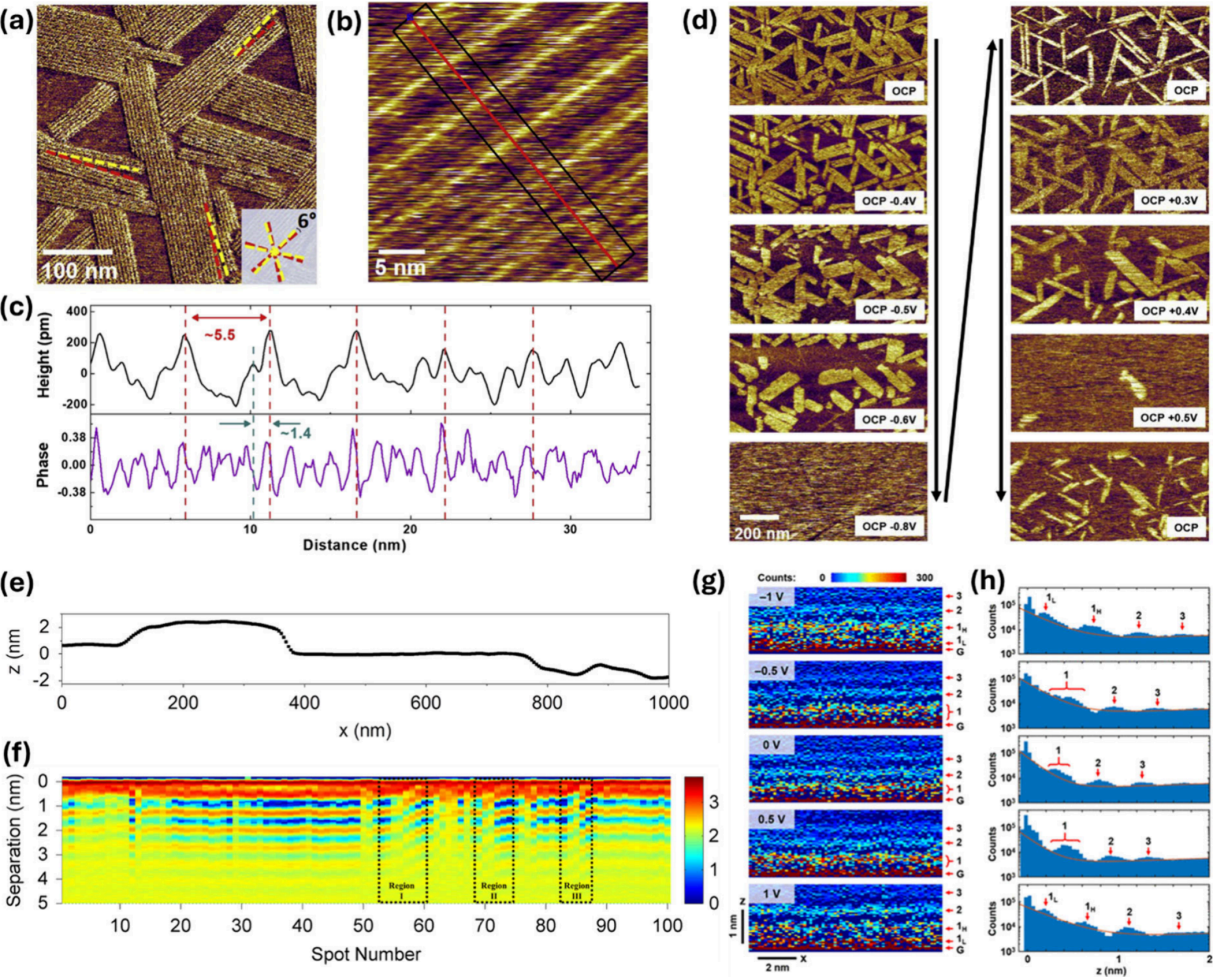
(a) AFM image of the first adsorbed layer of [PYR_14_^+^]­[TFSI^–^] on HOPG. Two subsets of orientations with an angle mismatch of 6° can be observed. (b, c) High magnification height image of the ordered domains, and height/phase profile across the red line. (d) Evolution of ordered domains with applied bias, across the trajectory denoted with arrows.^541^ Reproduced with permission from ref 541. Copyright 2019 Elsevier. (e, f) Line profile of HOPG of 100 sets of force–distance curves, and top-down view of separation histograms for all 100 locations (bar stands for the common logarithm of the data point frequency). Dislocation defects are observed in Regions I, II, and III.^538^ Reproduced with permission from ref 538. Copyright 2015 Elsevier. (g, h) Dependence of EDL x-z count maps on the applied potential, and corresponding point count histograms as a function of the distance from the electrode, z (orange curves were fitted to the local histogram minima). Arrows show the position of HOPG and of the first three adsorbed layers, with 1_L_/1_H_ representing the split lower/upper first adsorbed layer^542^ Reproduced with permission from ref 542. Copyright 2020 American Chemical Society.

While AFM force–distance curves cannot extract information on electrolytes under confinement, other modes of AFM can track the insertion of ions on porous networks through the monitoring of volume changes. Arruda and colleagues employed *in situ* AFM dilatometry to investigate the charging-induced electrochemical expansion of thin microporous carbon electrodes, at a deformation/strain resolution that is 2–3 orders of magnitude higher than that of electrochemical dilatometry, observing two distinct processes of EDLC charging, namely a fast process manifesting the EDL formation and a slow process related to ion transport within the porous network.^543^ While both of these processes contribute to capacitance, only the latter process was found to induce significant volume changes in porous carbon. Black et al. combined *in situ* AFM with molecular dynamics (MD) to study the ion flux of [EMI^+^]­[TFSA^–^] IL into mesoporous carbons with a narrow distribution of mesopores and varying micropore content, and to assess the pressure exerted on porous electrodes under applied potential.^544^ At anodic potentials, the carbon with the highest volume of small accessible micropores was associated with elevated mechanical stress, while decreasing the total amount of inserted ions (as the charging rate increases) leads to a reduction of the strain exerted on the carbon network.

### 5.7 Modeling, Simulations, and Data-Driven Approaches

The anomalous increase in the capacitance of carbons with subnanometer pore size, demonstrated two decades ago by Gogotsi et al., has triggered intensive research effort toward the improvement of theoretical EDL models and the prediction of optimized carbon’s pore structure and surface chemistry.^66^ Aiming to rationalize this phenomenon, which challenged the long-held axiom that pores smaller than the solvated ions are incapable of contributing to charge storage, Kondrat and Kornyshev used mean-field theory to introduce the concept of the “superionic state”,^71^ a state of broken Coulombic ordering that was recently confirmed via experimental SAXS by Kaneko and colleagues,^545^ in which the electrostatic interactions of ions inside electronically conductive nanopores are screened by the induction of image charges in the pore walls. While modeling for nanoporous carbon electrodes is quite challenging due to their complex geometry, simple one-dimensional (1D) and two-dimensional (2D) analytical models can provide valuable insights on the charging behavior in cylindrical and slit-like pores, respectively.^546^

Kornyshev studied the voltage-controlled accumulation of IL ions in single-file cylindrical pores (Figure 43a) using the simple 1D two-state Ising model (Figure 43b), which neglects interactions between next-nearest and higher-order neighbors, leveraging the analogy of the cation/anion species to the spin-up/spin-down configuration that is used in the theory of magnetism.^547^ Decreasing the pore size strengthens the Coulombic screening and increases the ion packing density boosting capacitance, whereas the dependence of the capacitance on voltage has the character of a smeared resonance whose position and intensity are strongly affected by the pore size (Figure 43c,d). Thus, even small pore size variations within the electrode soften this voltage dependence. Besides the impact of ion affinity to the charging behavior of single-file cylindrical pores, Lee et al. investigated the effect of ion affinity to the charging behavior of single-file cylindrical pores, concluding that while ionophilic pores can store more energy at low bias, their charging is slower than that of ionophobic pores.^550^ Zaboronsky extended the analysis of the superionic state to multifile nanopores that can accommodate multiple rows of ions by mapping the arrangement of ions in polarized electrodes on a multirow Ising model in an external field.^551^ While single-file pores elevate energy density at low applied voltage, multifile pores perform better as the voltage is increased. Expanding into 2D slit-like nanopores, Oshanin and colleagues applied a Bethe lattice approach to study confined ILs in the absence^552^ and presence^553,554^ of applied potential, identifying first-ordered and second-ordered transitions between its ordered and disordered state. If the confined ions are in the ordered state (which consists of two oppositely charged sublattices) in the absence of an applied voltage, the system remains in this state up to a certain applied voltage threshold that is required to break ordering, resulting in its disordered state, which is a homogeneous mixture of ions and solvent molecules.^554^

**Figure 43:**
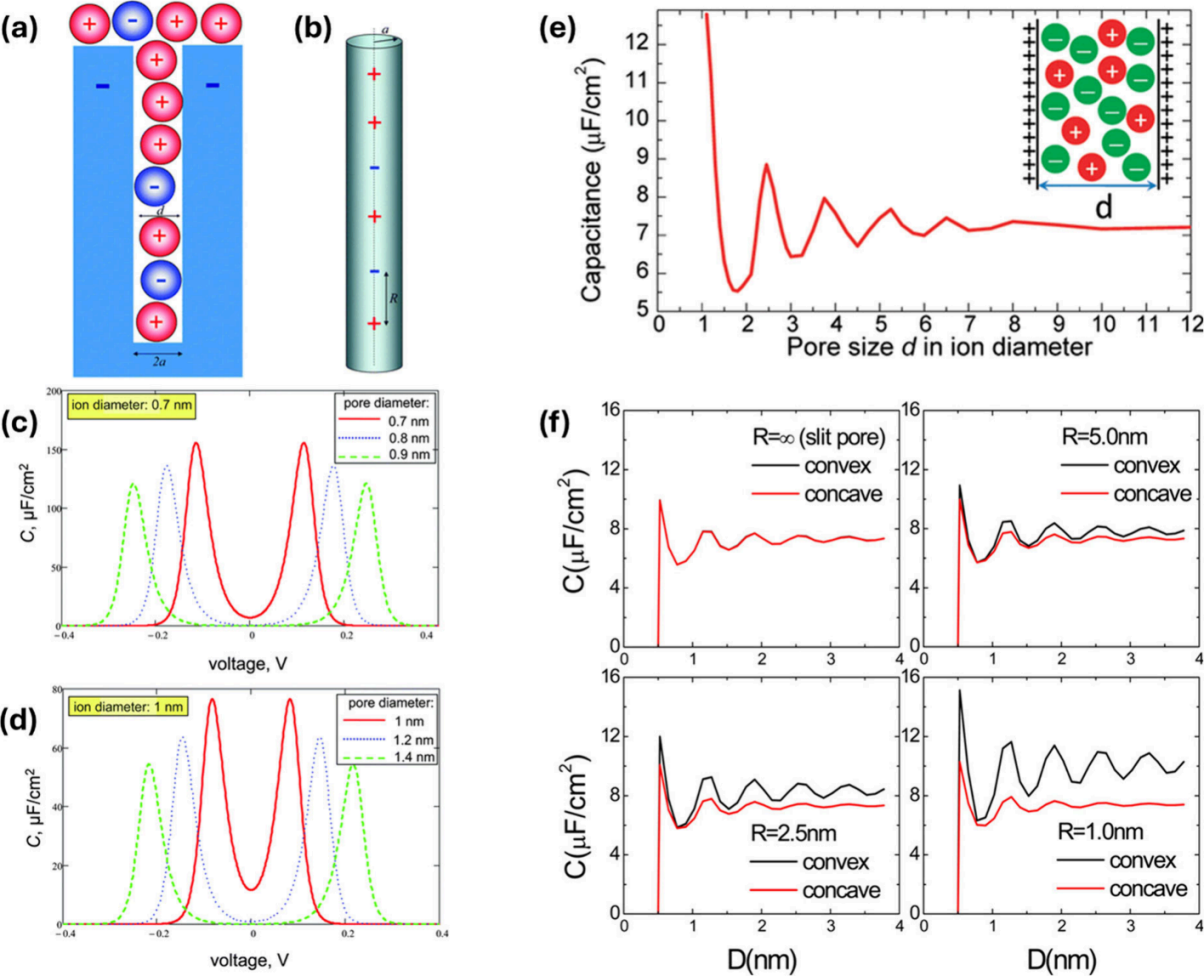
(a) Negatively charged single-filed cylindrical pore, (b) representation of ions in a 1D lattice, (c, d) Ising model-based dependence of capacitance on voltage for variable pore diameter, for an ion size of 0.7 and 1.0 nm, respectively.^547^ Reproduced with permission from ref 547. Copyright 2013 Royal Society of Chemistry. (e) Capacitance dependence on the pore size.^548^ Reproduced with permission from ref 548. Copyright 2011 American Chemical Society. (f) EDL capacitance vs pore size at convex and concave interfaces of a spherical shell, for various curvature radii (R).^549^ Reproduced with permission from ref 549. Copyright 2016 American Chemical Society.

Despite their attractive simplicity, analytical models often rely on several assumptions without considering microscopic details that may be important in highly confined electrolytes. Density functional theory (DFT) can overcome this limitation, being at the same time less computationally strenuous than simulations and thus constitutes a well-established approach for the study of confined electrolytes, including (but not limited to) the anomalous capacitance increase, solvent effects, and charging dynamics.

Jiang et al. used classical DFT to study the dependence of capacitance on pore size for an IL confined in narrow slit-like nanopores, predicting an oscillation of capacitance as a function of the pore size, exhibiting multiple capacitance peaks with a decaying envelope as the pore size increases (Figure 43e).^548^ This oscillatory behavior was assigned to the interference of the overlapping EDLs. Lian et al. investigated the impact of pore ionophobicity on the capacitance and charging processes for nonaqueous electrolytes confined in slit-like pores.^555^ Ionophobic pores exhibited a different charging behavior than that of ionophilic pores. Increasing ionophobicity was shown to change the shape of the capacitance–voltage curve from “bell-like” to a “camel-like” profile, with ionophobic pores providing higher energy density at high voltages. The same research group investigated the combined effect of pore size and curvature on capacitance by introducing a spherical shell model, observing a similar oscillatory behavior of capacitance with pore size, while capacitance was found to increase with pore curvature (Figure 43f).^549^ The impact of curvature on capacitance was more pronounced in convex interfaces, whereas the effect of pore size on capacitance was less significant at concave interfaces.

As showcased in the DFT study of Jiang et al., the presence of a polar solvent results in a considerable drop of the anomalous capacitance increase and in a damping of its oscillatory behavior, which is due to the formation of an EDL that consists of both counterions and highly organized solvent molecules.^556^ Later, the same authors showed that the capacitance as a function of the pore size may exhibit an oscillatory profile when the polarity of the solvent is reduced below a certain threshold.^557^ For a 4 nm wide pore, the dependence of the capacitance on the solvent’s dipole moment displays a volcano-shaped behavior, due to interplay of nanoconfined ions and solvent molecules. As polarity increases from a weak to moderate value, more solvent molecules enter the pores, aligning together with the counterions and decreasing the effective electrode-counterion distance, elevating capacitance. On the contrary, increasing the solvent’s dipole moment above a certain value (∼4 Debye) leads to an expulsion of counterions, decreasing capacitance. Liu et al. explored the effect of polar additives on the layering structure and the capacitance of IL-based supercapacitors with porous electrodes, showing that a highly polar, low-molecular-weight additive can drastically increase the EDL capacitance at low bulk concentration, at the same time dampening the oscillatory capacitance dependence on the pore size.^558^

To study the charging dynamics inside slit-shaped nanopores, Aslyamov et al. relied on the time-dependent DFT approach and described three consequent charging regimes, namely one initial root-square diffusion and two exponential regimes of different time scales, with the most significant part of the full charging inside ultranarrow pores being defined by the slower exponential trend.^559^ Qing and Jiang conducted a systematic study on the contribution of ion-size asymmetry to the charging behavior of a supercapacitor, using time-dependent DFT.^560^ Compared to the case of symmetric ion size, ion-size asymmetry has a “double-edged sword” effect, as it accelerates the charging process but reduces the differential capacitance (*C*_*diff*_.). Moreover, the authors showed that while ion symmetry yields a *C*_*diff*_. ∼ *t* relation, ion asymmetry can yield a highly desirable *C*_*diff*_. ∼ 1/*t* relation, breaking the tradeoff relation between energy and power density. Lian et al. studied the ionic transport through voltage-gated nanopores by combining DFT with the Navier–Stokes equation for the electroosmotic flow and showed that the voltage-dependent conductivity displays a nonmonotonous trend with the pore width, maximizing at a width of 1–2 nm.^561^

While DFT calculations is computationally efficient, this attribute comes at the cost of using basic models for the electrode and the electrolyte,^562^ while they rely on several approximations that often neglect fluctuations. Molecular dynamics (MD) and Monte Carlo (MC) simulations can overcome this limitation, handling more realistic representations of ions and molecules in highly complex systems such as nanoporous carbons.^546^

Following their mean-field theory study, rationalizing the anomalous increase of capacitance in nanoporous carbons through the formation of a “superionic state”,^71^ Kondrat et al. used grand canonical MC simulations to validate that the shielding of the electrostatic interactions in the pore interior can indeed lead to an anomalous capacitance increase.^72^ Wu et al. reproduced the anomalous capacitance enhancement below a critical pore width via MD simulations, revealing that for polarized pores wider than two ion diameters, counterions form two distinct layers whereas coions mainly occupy the pore center.^563^ Interestingly, the electrical potential at the pore center increases as the pore width varies from 0.75 to 1.26 nm while the total potential drop from the electrode surface to the bulk IL electrolyte is similar in all pores, indicating a qualitative breakdown of the transmission line model at narrow pores. Besides nanoconfinement, nanopore surface roughness is equally important for enhancing capacitance, as demonstrated by MD simulations conducted by Vatamanu and colleagues.^115^ Futamura et al. analyzed X-ray scattering data using hybrid reverse MC simulation to recover the structure of [EMIM]­[TFSI] confined in microporous CDC, showcasing a monolayer and bilayer ion arrangement in pores of 0.7 and 1 nm diameter (Figure 44 a-d).^545^

**Figure 44:**
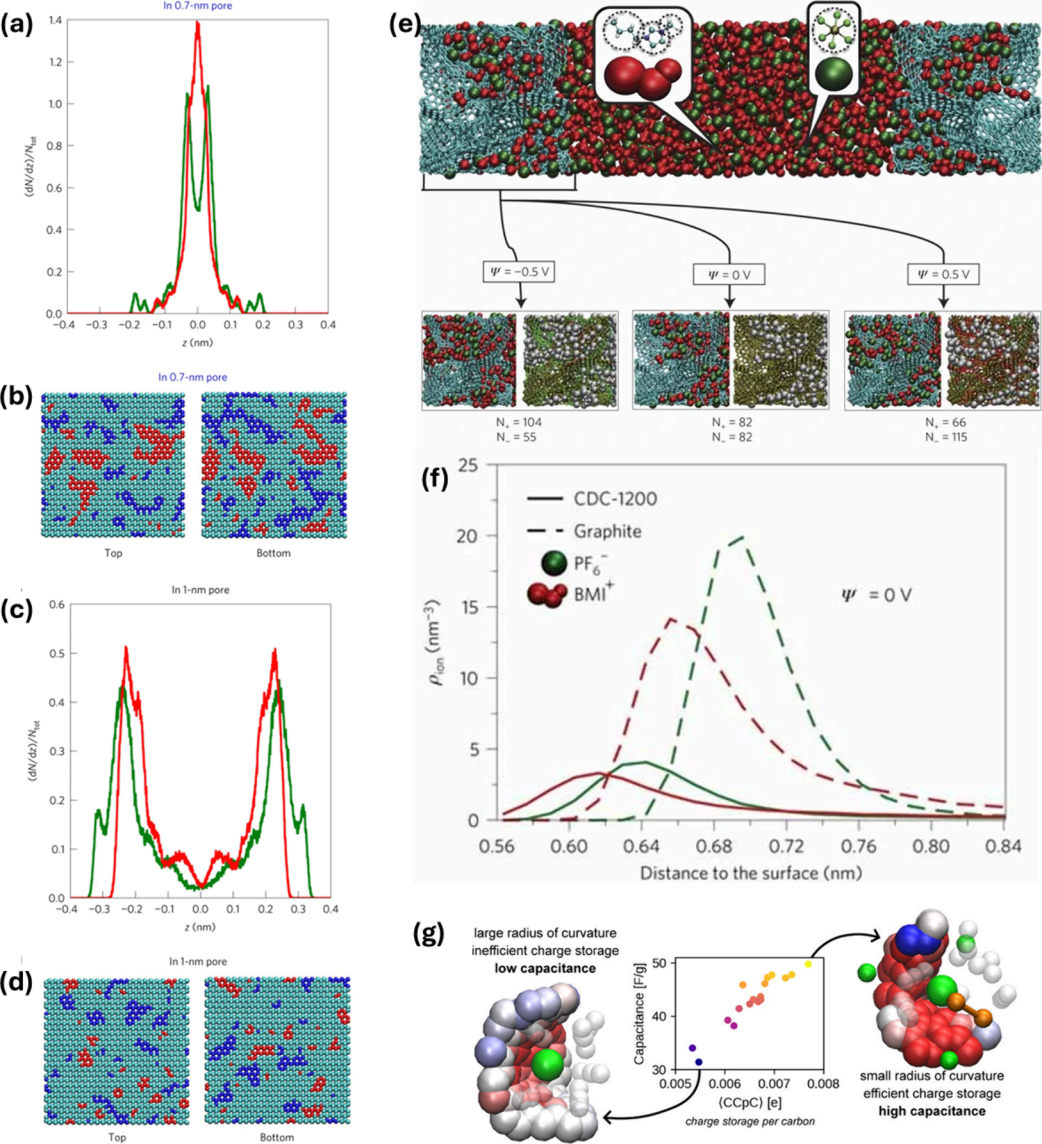
Density profiles of [EMI^+^] (green) and [TFSI^–^] (red) and induced charge distributions on pores walls, for (a, b) 0.7-nm, and (c, d) 1-nm pores (*z* represents the coordinate perpendicular to carbon walls, with *z* = 0 lying at the pore center). Carbon atoms in blue and red have high negative (*q* < −0.01*e*) and positive (*q* > 0.01*e*) charges, respectively, while cyan atoms have intermediate charges between blue and red atoms.^545^ Reproduced with permission from ref 545. Copyright 2017 Springer Nature. (e) Simulation cell of a [BMI]­[PF_6_] IL contained within two CDC electrodes at constant electrical potentials of 0 V, and ± 0.5 V (blue: C atoms, red: BMI^+^, green: PF_6_^–^).^87^ Reproduced with permission from ref 87. Copyright 2012 Springer Nature. (f) Comparison of ionic density profiles for BMI^+^ and PF_6_^–^ near graphite and CDC at 0 V.^87^ Reproduced with permission from ref 87. Copyright 2012 Springer Nature. (g) Correlation of capacitance with charge compensation per carbon atom.^564^ Reproduced with permission from ref 564. Copyright 2019 American Chemical Society.

Recently, Mo et al. studied the charging dynamics of [EMIM]­[BF_4_]-containing subnanopores with variable pore size using MD simulations, revealing that the pores with half-integer times the ion size can enhance the charging performance.^565^ Specifically, as the subnanometer pore width enlarges, there is an EDL structure transition from one layer to two layers with mixing cations and anions, achieving the optimum charge dynamics at 0.75 nm, whereas exceeding a width of 0.85 nm results in cations and anions forming two distinct in-pore layers, decelerating charging dynamics. Velpula and colleagues combined Raman spectroscopy, tight-binding DFT, and MD simulations to study the influence of the alkyl chain length of an IL ([C_n_MIM]­[BF_4_], n = 2,4,6,8,10) on the electronic properties of a graphene adsorbent.^566^ Regardless of the cation size, the ILs attain a layered arrangement, and the surface-dipole-induced electrostatic potential formed at the adsorbent–adsorbate interface was found to behave as a gate resulting in a n-type doped graphene, with the magnitude of n-type doping increasing with the chain length.

Salanne and colleagues studied the structure of [BMI]­[PF_6_] electrolyte confined in realistic CDCs via MD simulations, revealing that the electrode pores are wetted even in the absence of applied potential, with the charging process involving the ion exchange with the bulk electrolyte (accompanied by a partial decrease of the coordination number of ions) without changing the volume of liquid inside the electrode (Figure 44e).^87^ Interestingly, their comparison of CDCs with a planar graphite electrode revealed that the confinement associated with the former case prevents the occurrence of overscreening,^567^ decreasing charge separation (Figure 44f) and rendering charge storage more efficient. Moreover, comparison between different CDCs (CDC-950 and CDC-1200) with similar pore size distribution showcased the influence of local structure, as the substantially lower capacitance of CDC-1200 correlated with the greater occurrence of graphitic zones.^87^ In a follow-up study, the same group demonstrated the key role of the local structure and revealed the role of coordination and solvation, showing that the adsorbed ions can partition themselves among different adsorption sites of CDC (i.e., edge, plane, hollow, and pocket sites) in a manner that depends on the applied potential.^258^ By combining electrochemical experiments with MD simulations, Pean et al. showed that the diffusion of ions inside electrified nanoporous carbon electrodes is 1 order of magnitude slower than in the bulk electrolyte, and is characterized by a hierarchy of time scales arising from ion confinement, solvation, and electrosorption effects.^77^ An applied potential of −1 V slowed down in-pore diffusion by a factor of 4 (compared to 0 V; from ∼1 ns to ∼4 ns), while ion desolvation was identified as the fastest process (∼1 ps). Applied potential was also found to affect the ion adsorption kinetics. In the absence of applied potential, the characteristic adsorption time was ∼150 ps, while in the case of −1 V the adsorption of counterions and the desorption of coions are of much different time scales, namely ∼500 ps and ∼3 ps, respectively.^77^ Merlet and colleagues investigated the effect of surface chemistry on the properties of an IL in contact with different zeolite-templated carbons, namely a structure without any functional groups, a structure with ester, hydroxyl, anhydride acid, and carboxyl functional groups, and a structure where the oxygen and hydrogen atoms are replaced by carbon atoms (to distinguish between steric and specific effects of the functional groups).^568^ Their findings suggest that the presence of surface functional groups (-O and -H) has a limited impact on the structure of the confined electrolyte but improves the ion mobility. Liu et al. conducted MD simulations of EDLCs using a library of zeolite-templated carbons, showing that the capacitance does not correlate with any electrode geometric descriptor (density, void fraction, average pore size, and pore limiting diameter) alone.^564^ Instead, capacitance was found to exhibit a strong linear correlation with the “charge compensation per carbon” (CCpC), a new descriptor that is defined as the average charge of the electrode atoms within the coordination shell of a counterion (Figure 44g). Adsorption sites with high CCpC tend to be within pockets of small radius of curvature that promote counterion charge screening, enabling higher ion loading at a fixed voltage.^564^

Kondrat and colleagues used MD simulations and a mean-field theory model to study the charging dynamics of ultranarrow open slit-like pores with ILs, demonstrating that the self-diffusion coefficient varies nonmonotonically over a few orders of magnitude during the different states of charging, and can exceed the ion diffusion in the bulk for moderate charge.^92^ At the point of zero charge, counterions and coions are interlocked into each other, arranging into a two-dimensional ionic crystal, resulting in a self-diffusion coefficient that is 2 orders of magnitude lower than in the bulk. During charging, this highly ordered state gradually disappears due to the progressive counterion adsorption (accelerating diffusivity), whereas exceeding a certain charge threshold (hence counterion concentration) leads to the formation of a quasi-Wigner crystal with a small number of coions as impurities, decreasing the diffusion coefficient.^92^ Péan et al. conducted constant potential MD simulations to study the charging of electrified nanoporous CDC electrodes impregnated with a [BMI]­[PF_6_] IL, showing that the CDC electrode with the smallest average pore size has the slowest charging, and that sites that are more proximal to large pores can charge faster, regardless of whether they are located at inner regions of the electrode.^569^ Later, the same group studied the effects of solvation and of high potential on charging/discharging dynamics, identifying a stepwise mechanism for the charging process that consists of a very fast first regime and of a slower subsequent regime.^570^ In the case of a neat IL, charging involves ion-exchange, with the intersite reorganization and the desorption of coions being more sluggish than the reorganization and the adsorption of counterions, whereas the addition of solvent promotes the mobility of coions facilitating charging.

## 6 Summary and Outlook

SPCs derived from molecular and polymeric precursors have emerged as a powerful platform for engineering high-performance supercapacitors, offering unprecedented control over pore architecture, surface chemistry, and structural evolution. As detailed throughout this Review, advances in bottom-up synthesis, hierarchical design, and advanced characterization have significantly deepened our understanding of structure–transport–performance relationships.

However, despite these advances, a critical reassessment reveals that the field is approaching a conceptual bottleneck rather than a materials bottleneck. A central issue lies in the persistent disconnect between reported material-level performance and realistic device operation. Gravimetric capacitances exceeding 300–500 F g^–1^ are routinely achieved under low mass loading, thin electrodes, and excess electrolyte conditions. Yet, under practical conditions—high mass loading (>10 mg cm^–2^), thick electrodes (>100 μm), and limited electrolyte—capacitance retention frequently drops by 50–80%. This discrepancy indicates that a substantial fraction of reported “high performance” originates from measurement conditions rather than intrinsic materials design, and underscores the urgent need to redefine performance benchmarks toward volumetric, areal, and high-rate metrics.

Beyond this experimental inconsistency, a deeper limitation arises from the incomplete mechanistic framework guiding materials design. The widely adopted surface-controlled versus diffusion-controlled paradigm provides a useful phenomenological description but fails to capture the coupled processes of ion desolvation, confinement-induced restructuring, and local structural heterogeneity within nanopores. Increasing evidence suggests that pore accessibility, connectivity, and structural disorder—rather than surface area alone—govern charge storage behavior, yet synthetic strategies continue to prioritize surface area maximization. This mismatch highlights the lack of causal structure–property relationships, which remains a fundamental barrier to predictive design.

At a more fundamental level, the reliance on electric double-layer capacitance (EDLC) imposes an inherent limitation on energy density. While heteroatom doping is frequently invoked to introduce pseudocapacitive contributions, its role remains poorly defined. In most cases, performance enhancement cannot be unambiguously attributed to redox activity, wettability improvement, or electronic structure modification. More critically, the electronic structure of carbon frameworks—particularly the density of states near the Fermi level, the work function, and the associated quantum capacitance—remains largely underexplored as a unified design parameter. In this context, emerging concepts such as “reductive carbon” and “noble carbon” in which the electronic structure is deliberately tuned to modulate interfacial potential alignment, suppress self-discharge, and optimize ion–electron coupling, offer a new conceptual framework beyond conventional doping strategies. Such approaches highlight that future SPC design must move beyond geometric optimization toward electronic-structure-driven engineering, where charge storage is governed not only by accessible surface area but also by the energetic landscape at the electrode–electrolyte interface.

These limitations collectively point to several fundamental scientific challenges that define the next stage of SPC research. First, the coupling between ion transport and charge storage across multiple length scales remains unresolved. While hierarchical porosity is widely adopted, it is typically implemented as a statistical distribution of pore sizes rather than as a controlled transport network. Future progress requires shifting from pore size optimization toward pore connectivity and transport pathway engineering, particularly under a practical electrode thickness. Second, the field must move toward more precise control of the surface chemistry and electronic structure. Developing synthetic strategies that enable controlled doping and systematic tuning of electronic properties will be critical for establishing the causal design principles. Third, the challenge of scaling-precision synthesis remains unresolved. Many structurally well-defined carbons rely on complex templating and multistep processing that is incompatible with industrial production. Bridging structural precision with scalable and green synthesis remains essential for practical implementation.

Addressing these challenges requires a transition toward more integrated design strategies. One key direction is the development of data-driven materials design frameworks. Machine learning approaches, including emerging large language model-assisted strategies, offer new opportunities to navigate complex synthesis spaces and identify optimal structure–performance relationships. However, their predictive power depends critically on integration with in situ and operando characterization, which provide direct insight into ion dynamics and interfacial processes under realistic conditions. The combination of ML with operando techniques thus represents a powerful pathway toward establishing predictive and experimentally validated design rules.

Equally important is the need to treat the electrode and electrolyte as coupled systems rather than independent variables. Charge storage in porous carbons is fundamentally governed by the interaction between the pore structure and electrolyte ions, including ion size, solvation structure, and transport behavior. In this context, high-entropy electrolytes, together with ionic-mixture electrolyte systems, provide an expanded design space by introducing chemically diverse ion environments and tunable solvation structures. Matching the pore architecture with such complex electrolytes offers a promising pathway to simultaneously enhance energy density while maintaining fast ion transport.

The development of SPCs for supercapacitors has reached a stage where further progress cannot rely on incremental improvements in the surface area, pore size distribution, or doping content. Instead, it requires confronting the fundamental trade-offs that underpin electrochemical energy storage: between porosity and density, between accessibility and confinement, and between structural precision and scalability. Advancing beyond these limitations demands a transition from empirical optimization to mechanistically informed and integrated design, where pore architecture, electronic structure, and ion transport are considered interconnected systems spanning multiple length scales. By integration of precision synthesis, advanced characterization, and data-driven approaches, it becomes possible to establish predictive design principles that bridge the gap between molecular structure and device-level performance. Such a framework will be essential for transforming SPCs from high-performance model materials into practical energy storage platforms capable of delivering both high energy density and high power capability under realistic operating conditions.
